# AI digital pathology as a key tool providing in-depth understanding of the progression and regression of MASH and fibrosis in male mouse models

**DOI:** 10.1038/s41467-026-73370-z

**Published:** 2026-07-22

**Authors:** Ashmita Saigal, Desiree Abdurrachim, Emily Tso, Christopher Hendra, Charlene Zhi Lin Ong, Sonya Kimball, Wendy Blumenschein, Yang Liu, Xueyuan Jiang, Arun J. Sanyal, Asad Abu Bakar Ali, Saswata Talukdar

**Affiliations:** 1 Cardiometabolic Diseases, Merck & Co., Inc., South San Francisco, CA USA; 2 Quantitative Biosciences, MSD, Singapore, Singapore; 3 Non-clinical Drug Safety, Merck & Co., Inc, West Point, PA USA; 4 Translational Medicine (Profiling and Expression), Merck & Co., Inc, South San Francisco, CA USA; 5 Data, AI, and Genomic Sciences, Merck & Co., Inc, South San Francisco, CA USA; 6 Stravitz-Sanyal Institute for Liver Disease and Metabolic Health, Virginia Commonwealth University School of Medicine, Richmond, VA USA

**Keywords:** Drug discovery, Physiology

## Abstract

Optimizing murine models for studying Metabolic Dysfunction-Associated Steatohepatitis (MASH) and fibrosis is crucial for understanding disease mechanisms and evaluating therapies. In this study, we characterized diet-induced male murine models of MASH and liver fibrosis using an AI-digital pathology (DP) pipeline for zone-specific analysis and spatial (co)-localization of key MASH features within the liver microarchitecture, providing insights beyond standard histology. Model characterization included an integrative omics-based approach, standard blood-chemistry, and traditional histology. AI-DP revealed previously unknown temporal events leading to MASH, emphasizing the interplay between inflammation and dysmetabolism in disease progression. We also noted distinct morphometric characteristics of granulomas and their correlation with fibrosis. In efficacy studies of clinically-validated treatments, Semaglutide (GLP-1RA), Resmetirom (THRβ-agonist), and MK-4074 (Acetyl-CoA-carboxylase inhibitor), AI-DP demonstrated differential effects on macrosteatosis, microsteatosis, and colocalized fibrosis. Overall, integrating AI enables identification of fit-for-purpose disease models for therapeutic testing, and facilitates robust preclinical study designs for advancing effective therapeutic strategies.

## Abstract (web summary)

AI-driven multimodal pathology enables zone-resolved colocalization and objective reproducible scoring of spatial and treatment-specific effects to improve murine MASH model characterization.

## Introduction

MASH is characterized by liver inflammation and damage caused by the accumulation of fat, particularly in individuals with risk factors such as obesity, and impaired glucose metabolism. These factors along with genetic pre-dispositions, ultimately contribute to the progression of liver fibrosis^1^.

Conventional detection of MASH and liver fibrosis leverages imaging modalities, including ultrasound, computed tomography (CT), and magnetic resonance imaging (MRI), however, a definitive diagnosis requires histopathological analysis of liver biopsy which remains a ‘tarnished’ gold standard, owing to inherent variability. Interobserver differences, and conventional ordinal scoring approaches such as NAFLD Activity Score (or NAS) for assessing Metabolic-Associated Steatotic Liver Disease (MASLD) and MASH, and METAVIR for assessing liver fibrosis from biopsies may not be representative^2,3^, and make efficacy comparisons across therapeutic candidates less robust. Such discrepancies can have profound implications on clinical outcomes and therapeutic decision-making. An artificial intelligence (AI)-driven DP offers numerous advantages in mitigating these challenges posed by traditional diagnostic and evaluation methods.

In addition to enhanced diagnostic accuracy and continuous (quantitative) scoring of liver pathology that follows a zonal pattern in progression, AI-driven approaches allow identification of subtle changes within the liver microarchitecture that may be overlooked by the human eye^4^. In a post-hoc analysis of a clinical trial involving tropifexor (TXR), the anti-fibrotic effect of TXR was not evident through conventional pathologist scoring. However, an AI-based evaluation of fibrosis reduction near areas of steatosis and ballooning revealed the anti-fibrotic effect^5^, highlighting the importance of investigating spatial colocalization of the liver microenvironment features. Similarly, AI-driven DP identified F3 patients as the best responders to Rezdiffra in the Phase 2b trial, enabling the Phase 3 trial to include over 51% F3 patients. This highlights the role of AI in enhancing clinical trial design and effectively stratifying patient populations. Rezdiffra was approved by FDA as the first and only treatment for adults with MASH and liver fibrosis (without cirrhosis) exemplifying the valuable insights revealed by AI-DP^6,7^.

Owing to multi-factorial nature of MASH and liver fibrosis^8^, drug discovery efforts involve looking at various pathways that drive disease progression^9^. Hence, utilization of relevant murine disease models^10,11^ that mimic hepatic manifestations (steatosis, inflammation, ballooning, fibrosis) in humans, is key in gaining mechanistic insights and testing therapeutic candidates. There are several rodent models of MASH/liver fibrosis, such as diet induced (e.g., western diet, high fat high cholesterol diet, choline deficient amino acid restricted diet), toxin induced (e.g., carbon tetra chloride (CCl4)), or drug (Streptozotocin) induced. Two of the most commonly used models: Gubra Amylin NASH (GAN) and Choline Deficient Amino Acid-defined High Fat Diet (CDAHFD) have contrasting yet complementary disease manifestation: GAN better models chronic metabolic disease processes relevant to human overnutrition, while CDAHFD is a rapid, fibrosis focused model useful for studying mechanisms of injury and fibrogenesis. The GAN diet (high fat, fructose, and cholesterol) induces steatosis via metabolic overload characterized by lipid accumulation, oxidative stress^12^, and immune activation leading to hepatocyte ballooning, chronic inflammation, stellate cell activation, and gradual fibrogenesis, mirroring dysmetabolism driven human MASH. In contrast, CDAHFD acutely impairs lipid export, phospholipid/membrane synthesis and mitochondrial function, triggering oxidative stress, permeability transition, rapid hepatocellular injury, and brisk fibrosis within weeks^13–15^. Operating via toxin like mechanisms and lacking systemic features (weight gain, insulin resistance), CDAHFD only partially captures the metabolic milieu of human MASH.

While MASH animal models are increasingly available, there remains a critical need for a comprehensive framework to characterize these models. An AI-driven approach that interrogates the spatial localization of liver microenvironment features, zone-dependent disease progression, and underlying mechanistic drivers is essential yet currently absent. Here, we developed and utilized a multi-modal AI-based DP pipeline to quantify zone-specific features of steatosis, inflammation, and fibrosis within the liver microenvironment, as well as the colocalization of these features to better understand the role of steatosis (and its sub-features) and inflammation in the progression of fibrosis. This approach offered novel and unprecedented insights into disease biology, enabling us to identify fit-for-purpose disease models for testing therapeutic interventions aimed at specific mechanisms or disease pathways, thus potentially bridging some translational gap.

In this study, 11 diet induced male murine models (Table 1) were developed at standard ambient (TA) and thermoneutral (TN) housing temperatures, and were thoroughly characterized via conventional histopathology, blood chemistry, and omics (Bulk RNA sequencing, lipidomic and metabolomic) based approaches for initial assessment. Based on the manifestation of metabolically relevant signatures from the initial evaluation, five models were evaluated by the AI-based DP approach: GAN-TA, GAN-TN, CDAHFD-TA, CDAHFD-TN, and CDAHFD-TA in aged mice. The AI-pipeline was then validated in efficacy studies testing clinically relevant compounds such as GLP-1RA (semaglutide)^16^, THRb agonist (resmetirom)^17^, and MK-4074 (ACCi)^18^. Taken together, our approach holds immense potential to improve detection, diagnosis, and management of MASH/fibrosis, effectively addressing many of the limitations inherent in conventional methods.

**Table 1:** Summary of histopathology across different murine models of MASH/MASLD

Housing Temp	Animal Model	NAS	Steatosis	Inflammation	Ballooning	PSR (%Area)
**TA**	*CDAHFD*	6.6 ± 0.84 (wk4)	3 ± 0 (wk4)	2.8 ± 0.42 (wk8)	1.3 ± 0.48 (wk16)	16.39 ± 2.91 (wk16)
	*CDAHFD (Aged)*	7.8 ± 0.42 (wk8)	3 ± 0 (wk2)	2.9 ± 0.32 (wk8)	1.9 ± 0.32 (wk8)	12.42 ± 2.32 (wk12)
	*GAN*	6.17 ± 0.75 (wk32)	3 ± 0 (wk28)	1.83 ± 0.41 (wk32)	1.33 ± 0.52 (wk32)	3.94 ± 1.94 (wk32)
	*GAN + Low dose CCl4*	6.43 ± 0.79 (wk12)	3 ± 0 (wk8)	2.57 ± 0.53 (wk12)	1.86 ± 0.38 (wk12)	12.51 ± 3.87 (wk24)
	*GAN (ob/ob)*	5.13 ± 1.46 (wk2) − 6.25 ± 0.46 (wk31)	3 ± 0 (wk2)	2.13 ± 0.35 (wk16)	1.25 ± 0.46 (wk24)	13.66 ± 2.21 (wk31)
	*FDSW*	6.38 ± 1.06 (wk23)	3 ± 0 (wk16)	1.75 ± 0.71 (wk23)	1.63 ± 0.52 (wk23)	3.79 ± 3.35 (wk23)
	*FDSW + Low dose CCl4*	5.88 ± 0.35 (wk8)	3 ± 0 (wk12)	2 ± 0 (wk18)	1.29 ± 0.49 (wk24)	6.44 ± 3.13 (wk24)
	*Chow (ob/ob)*	3.5 ± 1.22 (wk31)	3 ± 0 (wk12)	0.83 ± 0.41 (wk31)	0 ± 0 (wk31)	2.92 ± 0.68 (wk31)
**TN**	*CDAHFD*	8 ± 0 (wk4)	3 ± 0 (wk4)	3 ± 0 (wk4)	2 ± 0 (wk4)	17.67 ± 2.37 (wk16)
	*GAN*	6.86 ± 0.69 (wk32)	2.88 ± 0.35 (wk12)	2.14 ± 0.38 (wk32)	1.71 ± 0.49 (wk32)	6.02 ± 2.12 (wk32)
	*FDSW*	5.75 ± 1.04 (wk23)	3 ± 0 (wk16)	1.63 ± 0.52 (wk23)	1.25 ± 0.46 (wk23)	3.16 ± 1.28 (wk23)

Data expressed as Mean ±S.D.

*GAN* Gubra Amylin NASH (D09100310).

*FDSW* Friedmann Diet (TD.120528) + Sugar Water (23.1 g/L d-fructose & 18.9 g/L d-glucosa).

*CDA-HFD* Choline Deficient Amino-acid defined – High Fat Diet (A06071302).

*TA* Sub-thermoneutral Housing temp (20-22 °C).

*TN* Thermonuetral Housing temp (28-30 °C).

## Results

### Development of a multi-modal AI-driven DP pipeline

**Fig. 1:**
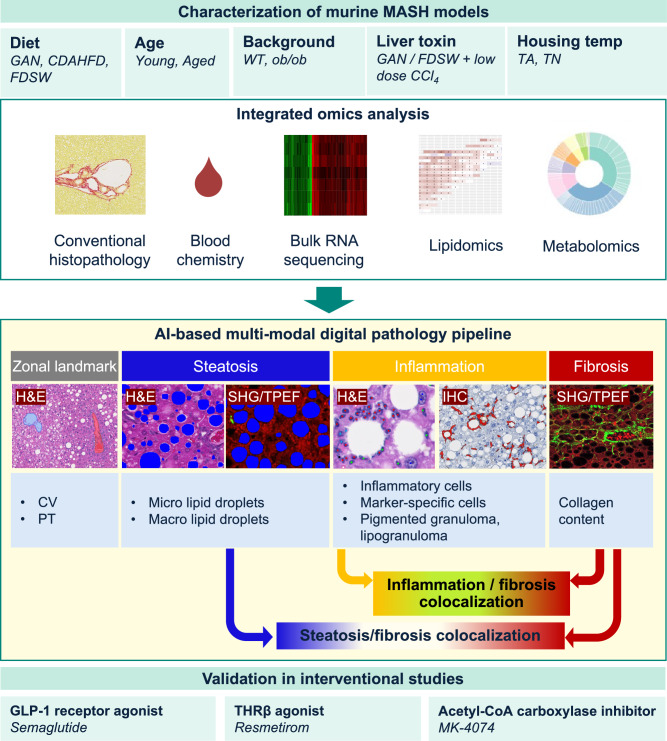
Integrated multi-modal analysis pipeline for characterization of murine MASH models and validation in interventional studies. Integrated omics analysis includes conventional histopathology, blood chemistry, bulk RNA sequencing, lipidomics, and metabolomics. AI-based multi-modal DP pipeline includes the detection of CV and PT as liver zonal landmark on H&E images, detection and quantification of steatosis on H&E and SHG/TPEF images, inflammation on H&E and IHC images, and fibrosis on SHG/TPEF images, with a downstream image co-registration module for feature colocalization analysis.

Our multi-modal AI-driven DP pipeline was developed for the automated detection and quantification of zone-specific steatosis, inflammation, and fibrosis using H&E-stained, IHC-stained, and unstained SHG/TPEF images (Fig. 1). This pipeline features novel parameters for detecting central and portal veins to delineate zones or lobules, and for assessing micro- and macro-steatosis, lipid size and distribution, inflammatory cell density/cluster, and granulomatous structures. It also evaluates aggregated and distributed fibrosis along with their sub-features. To analyse the contributions of steatosis and inflammation to fibrosis development, the pipeline includes co-localization analysis of features (e.g., steatosis/fibrosis and inflammation/fibrosis) and a co-registration module for features from different image types.

The AI-DP was applied across various murine disease models (GAN and CDAHFD, both young and aged) at different housing temperatures (TA or TN) and validated in efficacy studies, as discussed subsequently.

### Key insights from histopathology on environmental influences in MASLD/MASH progression

Histopathology is the gold standard for evaluating MASLD/MASH disease burden and served as the primary readout for the in vivo studies. At TA, conventional histopathology scores indicated the highest steatosis grades across all animal models at relevant time points, with similar levels of inflammation and ballooning (Table 1). Fibrosis assessment revealed the highest burden in CDAHFD-fed wildtype (WT) mice, followed by GAN-fed ob/ob mice and GAN-fed WT mice injected with CCl4. GAN-fed WT and FDSW-fed WT mice exhibited similar fibrosis development, ~3–4.2 times lower than that of CDAHFD-fed WT mice, depending on housing temperature (Table 1). Consistent with literature reports^19^, housing animals at TN exacerbated disease progression and overall burden, as reflected in changes in blood chemistry (Table S1), and histopathology. Bulk RNA sequencing corroborated these observations. GAN diet induced transcriptional changes at TN which worsened from week-16 to week-32, peaked at week-23 with 1305 Differentially expressed (DE) genes at TN vs. 836 DE genes at TA, followed by deceleration in changes thereafter (Supplementary Fig.1A). Additionally, KEGG and Gene Ontology (GO) enrichment analyses highlighted unique signatures for each housing conditions. While cardiac hypertrophy, fatty acid degradation, and inflammatory bowel disease were linked to TA, metabolically relevant signatures for Type II Diabetes, inflammasome activity, and fatty acid elongation were exclusive to TN (Supplementary Fig. 1B). Notably, uniquely enriched pathways at TN are central to MASH pathogenesis suggestive of physiological relevance of housing conditions on disease etiology.

GAN-fed animals at TN reached Grade 3 steatosis in just 4-weeks, while those at TA took 8-weeks. By 16-weeks on GAN, 100% of TN-housed mice achieved the maximal score, compared to 28-weeks for TA-housed mice. Lipidomic analysis revealed that feeding on GAN diet upregulated short-chain, highly saturated TAGs and downregulated long-chain, low-saturation TAGs, particularly at TN (Supplementary Fig. 1D) consistent with increased lipogenesis and reflective of hyperinsulinemia-driven SREBP-1 activation. While differences in housing temperature were reflected modestly in metabolomic analysis, yet notable upregulation of sphingolipid synthesis (Supplementary Fig. 1E), including 1-deoxysphinganine (m18:0) and sphingomyelin, explain dietary impact in contributing to systemic insulin resistance, dysregulated lipid accumulation, obesity-induced endothelial dysfunction, and atherosclerosis^20^. Several amino acid, drug metabolism and vitamin/co-factor related pathways were downregulated in liver (vitamin A, alanine, aspartate, glutamate, histidine, benzoate), and plasma (tyrosine, alanine, aspartate, lysine, phosphatidylglycerol (PG), phosphatidylethanolamine (PE)) - changes that can promote hepatic steatosis, ferroptosis, and disrupted redox homeostasis^21,22^. Top differentially expressed metabolites in the liver and plasma following GAN diet feeding are summarized in (Supplementary Fig. 1F).

Average pathologist scores for inflammation and ballooning were 1.2-fold and 1.3-fold higher (non-significant) at TN than at TA by the end of study. CDAHFD-fed mice reached a maximum NAS score of 8 at TN within 4-weeks. The impact of housing temperature was less significant in this model, likely due to its aggressive characteristics. In the following sections, we will conduct a detailed evaluation of GAN-fed WT mice and CDAHFD-fed WT mice using AI-driven DP approach.

### AI-DP reveals temporal and spatial localization of steatosis development in different diet induced models of MASH and liver fibrosis

**Fig. 2:**
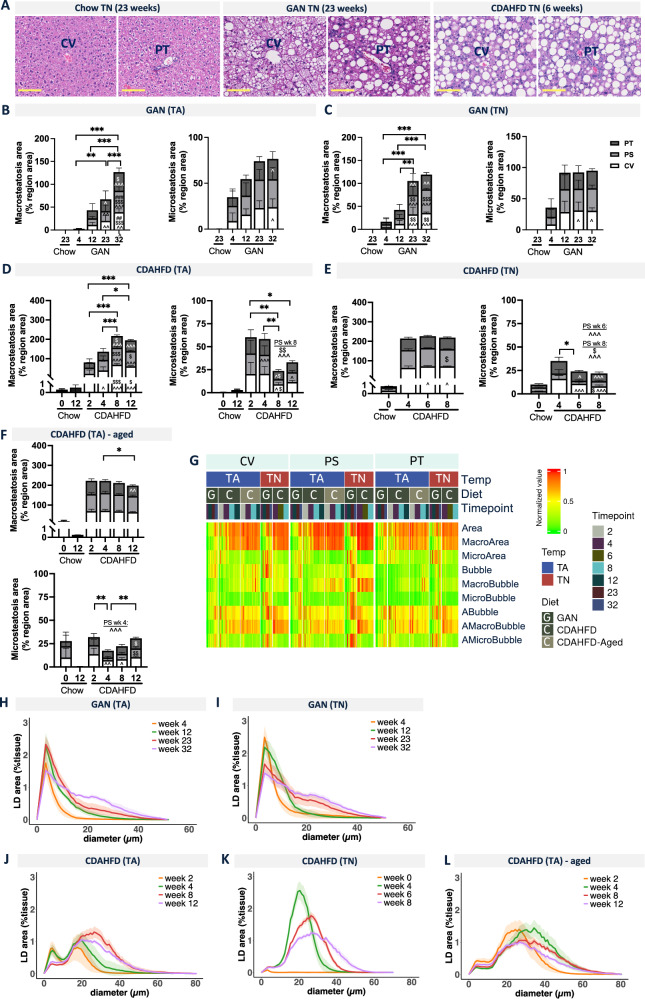
Steatosis quantification using AI-DP. **A** Representative H&E images for the different animal models, showing the development of microsteatosis and macrosteatosis surrounding CV (left panel) and PT (right panel); yellow bars represent 100 µm. **B**–**F** Quantification of macrosteatosis area and microsteatosis area for the different mouse (C57BL/6 J) models at TA and TN based on SHG. **G** Heatmap of steatosis parameters, the parameters are normalized within each zone separately based on SHG. **H**–**L** Lipid droplet distribution for the different animal models (diameter is binned for every 1 µm with a 3-point running average for the *y*-axis; solid colors indicate means, shades indicate confidence interval), based on H&E. Data in barplots is shown as means ± SD; B: GAN *N* = 5-6 per timepoint, Chow *N* = 4; C: GAN *N* = 6–7 per timepoint, Chow *N* = 4; **D** CDAHFD *N* = 10 per timepoint, Chow *N* = 10; E: CDAHFD *N* = 10, Chow *N* = 3 per timepoint;: CDAHFD *N* = 7–10 per timepoint, Chow *N* = 9–10 per timepoint. Statistical significance (one-way ANOVA, Bonferroni post-hoc analysis) was shown for comparisons within MASH diet group only, with symbols on top of the bars (*) indicate the comparisons for total steatosis (in overall liver), while those inside the bars indicate the comparisons for the corresponding zone (^: comparison vs. the first timepoint in the diet group, $: vs. the second timepoint, #: vs. the third timepoint, &: vs. the fourth timepoint), the number of symbols indicates. Age: 7 weeks upon start of GAN diet (TA/TN) and CDAHFD (TN); 8 weeks upon start of CDAHFD (TA); 77 weeks upon start of CDAHFD (TA, Aged).

AI-DP can differentiate between macro- and micro-steatosis, and provide continuous scoring to evaluate steatotic burden across the liver’s zonal microarchitecture. This contrasts with conventional histopathology, which, under the NASH-CRN framework, yields only ordinal, semi-quantitative categorical scores for whole-liver steatosis. We used H&E-stained image to qualitatively illustrates the steatotic burden and evaluate its temporal progression in the two disease models, GAN and CDAHFD, relative to lean, healthy mice (Fig. 2A and Supplementary Figs. 2, 3).

The progression of steatosis is accelerated in GAN-fed mice at TN with macrosteatosis evident at 4-weeks at TN, but not at TA (Fig. 2B, C). Microsteatosis plateaus by week-12 at TN (*P* > 0.99 vs. Week-23, Fig. 2C) suggesting temporal sequence in steatosis development where microsteatosis stabilizes while macrosteatosis continues to rise. This growth in lipid-droplets may be explained by droplet coalescence/fusion, diffusion of neutral lipid between lipid-droplets^23^, or direct lipid synthesis on droplet surface^24^. These mechanisms of lipid growth may show differential zonal susceptibility along liver’s metabolic gradient. Notably, steatosis development peaks in the perisinusoidal space, consistent with clinical data showing greatest efficacy within this zone.

De novo lipogenesis (DNL) favors production of saturated fatty acids with a relative loss of polyunsaturated fatty acids (PUFAs), consistent with our lipidomic and metabolomic data (Supplementary Fig. 1D, F). In GAN-fed mice at weeks-12 and 23, microsteatosis is more advanced at TN than TA (Fig. 2B, C) paralleling stronger fatty acid saturation and shorter chain lengths at TN in lipidomics (Supplementary Fig.1D) and downregulation of long chain PUFAs in metabolomics (Supplementary Fig. 1F). These patterns suggest that regions with prominent microsteatosis show biochemical signatures of enhanced DNL, altered fatty acid remodeling, and metabolic features linked to lipotoxicity and steatohepatitis progression. Such mechanistic claims can be confirmed via spatial co-registration of molecular data with histologic AI segmentation.

Interestingly, the kinetics of steatosis development vary across different models. In the CDAHFD model (Fig. 2D–G), there is a sharp increase in microsteatosis at 4-weeks under TN conditions ( ~ 350% vs. chow, *P* = 0.04), followed by a steep decline starting from week-6 (45% reduction vs. week-4; *P* < 0.05) and a concomitant rise in macrosteatosis that plateaus by week-4. This progression is more advanced compared to the TA model, where progression of macrosteatosis is delayed and attains saturation by week-8 (65% increase at week-8 vs. week-4, *P* < 0.001) instead.

Aged mice fed CDAHFD at TA exhibit exacerbated disease etiology, showing macrosteatosis saturation by 2-weeks on the diet (*P* > 0.99 vs. week-4, Fig. 2F) and larger lipid-droplets starting week 2, sooner than the younger mice (Fig. 2J–K). Furthermore, lipid-droplet area and size are greater in the CDAHFD model than in the GAN model (Fig. 2J–L vs. H–I) indicative of a higher lipotoxic burden. This distribution is also less variable at TN than at TA.

### Inflammation quantification using AI-DP

**Fig. 3:**
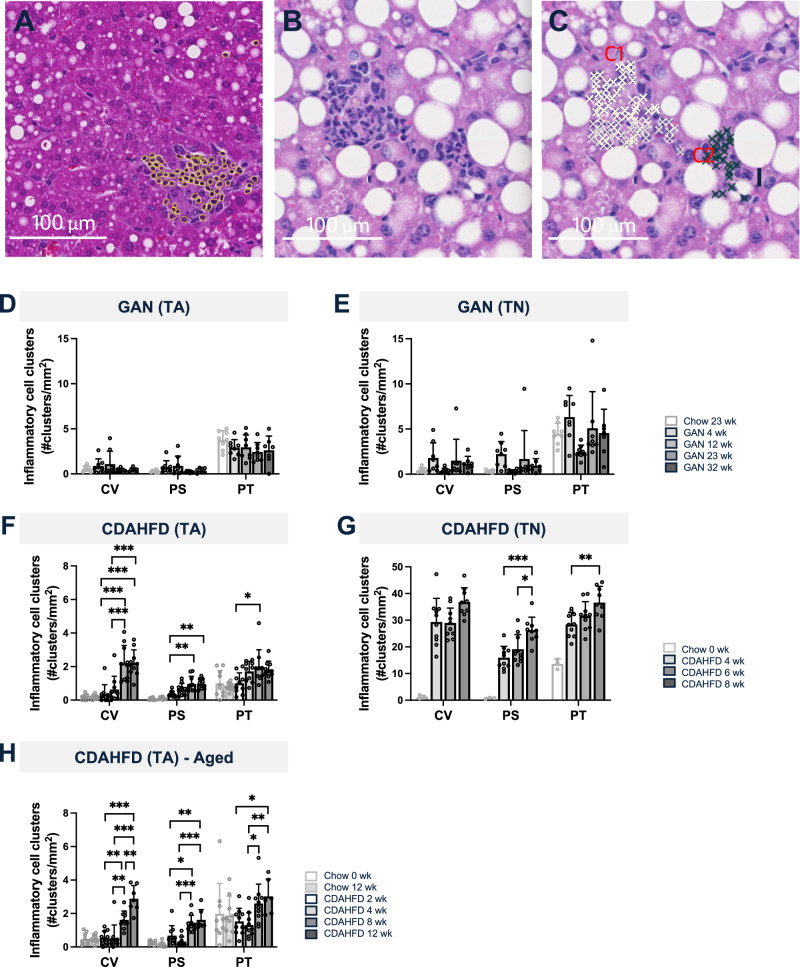
Inflammation quantification using AI-DP. **A** Illustration of detection of the individual inflammatory cells, **B** an example of two clusters of inflammatory cells, with the cells belonging to the same clusters are indicated by cross marks with the same color in (**C**). **D**–**H** The quantification of inflammatory cell clusters for the different mouse (C57BL/6 J) models. Data is shown as means ± SD; **D**–**E** GAN *N* = 7-8 per timepoint, Chow *N* = 8; F: CDAHFD *N* = 10 per timepoint, Chow *N* = 10; G: CDAHFD *N* = 10 per timepoint, Chow TA *N* = 3 per timepoint, H: CDAHFD *N* = 7-10 per timepoint, Chow *N* = 9 per timepoint. Statistical significance (one-way ANOVA, Bonferroni post-hoc analysis) was shown for multiple comparisons within each zone and within MASH diet group, *P < 0.05, **P < 0.01, ***P < 0.001. Age: 7weeks upon start of GAN diet (TA/TN) and CDAHFD (TN); 8weeks upon start of CDAHFD (TA); 77weeks upon start of CDAHFD (TA, Aged).

We developed a deep learning model for detecting inflammatory cells. The models achieved ROC AUC/PR AUC scores of 0.92/0.93. The model reliably detected inflammatory cells in both normal hepatocyte areas (Fig. 3A) and steatotic regions (Fig. 3B). The model quantifies inflammatory clusters, like pathologist evaluation of inflammatory foci for assessing lobular inflammation (Fig. 3C) and shows a strong correlation with pathologist’s inflammation scores (Spearman’s R = 0.82 for inflammatory cell density *P* < 0.0001, Supplementary Fig. 4A; Spearman’s R = 0.84 for PS inflammatory cell clusters *P *< 0.001, Supplementary Fig.4B; and Spearman’s R = 0.83 for inflammatory cells detected using multiplexed-IF for T-cell and myeloid panels *P* < 0.001, Supplementary Fig. 4C).

AI-driven DP indicated a linear increase in inflammatory burden across various zones at 8-weeks on a CDAHFD diet in both young and aged mice ( ~ 2.0–2.6-fold higher at week-8 vs. week-2, *P* < 0.05; Fig. 3F, H). This response was more pronounced in mice housed at TN, with a significant rise in inflammatory cell cluster observed as early as 4-weeks (TN: 22.5-fold, TA: 10.3-fold compared to respective chow, *P* < 0.001; Fig. 3G). In contrast, the GAN model exhibited greater variability at earlier time points, particularly at weeks-4 and 12 for TA (Fig. 3D)and at week-4 for TN (Fig. 3E)and a less dramatic temporal response compared to the aggressive CDAHFD model. This variability observed, especially at TN, was consistently captured by both, AI-DP and conventional histopathology (Supplementary Fig. 5), supporting the AI pipeline’s sensitivity to biologically plausible fluctuation. In the GAN model, inflammation was higher relative to chow when PS and CV regions were combined, areas pertinent to lobular inflammation, at TN (week-32: 2.8-fold vs. chow, *P* = 0.006; Fig. 3E), but not at TA (week-32: *P* = 0.98 vs. chow; Fig. 3D) consistent with the differential effect of housing temperature on disease progression.

### AI-DP reveals temporal and spatial localization of fibrosis development in different diet induced models of MASH and liver fibrosis

**Fig. 4:**
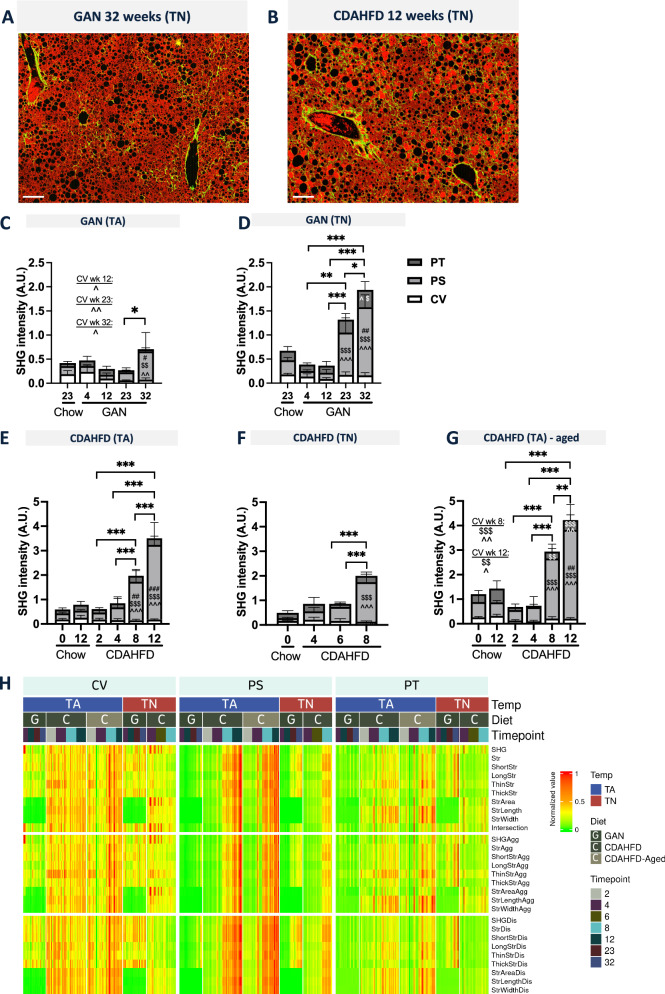
Fibrosis quantification using AI-DP. **A**, **B** Representative SHG/TPEF images. **C**–**G** Quantification of fibrosis in CV, PS, PT for different mouse (C57BL/6 J) models: (**C**, **D**) GAN diet, (**E**, **F**) CDAHFD, or (**G**) CDAHFD-aged, at thermo-ambient (TA; **C**, **E**, **G**) or thermo-neutral condition (TN; D, F) based on SHG images. (**H**) Heatmap of fibrosis parameters, the parameters are normalized within each zone separately. Data is shown as means ± SD; C: GAN *N* = 5-6 per timepoint, Chow *N* = 4; D: CDAHFD *N* = 6–7 per timepoint, Chow TN *N* = 4 per timepoint; E: CDAHFD and Chow *N* = 10 per timepoint, **F** CDAHFD *N* = 10 per timepoint, Chow *N* = 3 per timepoint; G: CDAHFD *N* = 7–10 per timepoint, Chow *N* = 9 per timepoint. Statistical significance (one-way ANOVA, Bonferroni post-hoc analysis) was shown for comparisons within MASH diet group only, with symbols on top of the bars (*) indicate the comparisons for the total bars (overall liver), while those inside the bars indicate the comparisons for the corresponding zone (^: comparison vs. the first timepoint in the diet group, $: vs. the second timepoint, #: vs. the third timepoint, &: vs. the fourth timepoint), the number of symbols indicates significance levels: 1: *P* < 0.05, 2: *P* < 0.01, 3: *P* < 0.001. White bars indicate 150 mm. Age: 7 weeks upon start of GAN diet (TA/TN) and CDAHFD (TN); 8 weeks upon start of CDAHFD (TA); 77 weeks upon start of CDAHFD (TA, Aged).

AI-driven DP can analyse liver tissue fibrosis by assessing various features across different zones in unstained slides using Second Harmonic Generation (SHG) and Two-Photon Excited Fluorescence (TPEF) microscopy (Fig. 4A-B). Distinct patterns of fibrosis and disease progression are observed in various murine models. For example, GAN-fed mice at TA show a significant reduction in fibrosis around the central vein (Zone 3) up to 23-weeks (3.9-fold lower at week-23 vs. week-4, *P* = 0.015; Fig. 4C) but a dramatic increase in fibrosis in the perisinusoidal space (Zone 2) by week-32 (4.7-fold higher at week-32 vs. week-4, *P* = 0.009; Fig. 4C). In contrast, the TN model exhibits accelerated disease progression, with a significant increase in fibrosis in Zone 2 by week-23 (2-fold vs. week-23 chow, *P* = 0.03; 4.9-fold vs. week-23 TA, *P* = 0.002; Fig. 4C, D), highlighting the translational relevance of these findings^4,5,25^.

The CDAHFD model demonstrates pronounced fibrosis, particularly in Zone 2, within week-12, regardless of housing temperature or animal age (11.9-fold at week-12 CDAHFD-young, 14.4-fold at week-12 CDAHFD-Aged vs. week-12 GAN-TA, and 5.5-fold at week-8 CDAHFD-TN vs. week-12 GAN-TN; *P* < 0.001, Fig. 4E–G vs. Figure 4C, D). Advanced fibrosis around the portal tract (Zone 1) further emphasizes the severity of this model. The heatmap (Fig. 4H) shows that advanced fibrosis is exclusive to the CDAHFD model at TA and both models at TN, especially after week-23 for GAN-TN and after week-8 for CDAHFD-TN. Fibrosis in Zone 1 is characterized by an increase in thin collagen fibrils and their aggregation, changes that are often undetectable by conventional histopathology, limiting analysis sensitivity.

### Spatial colocalization of steatosis, inflammation, and fibrosis by AI-DP reveals mechanistic drivers of disease progression in murine models

**Fig. 5:**
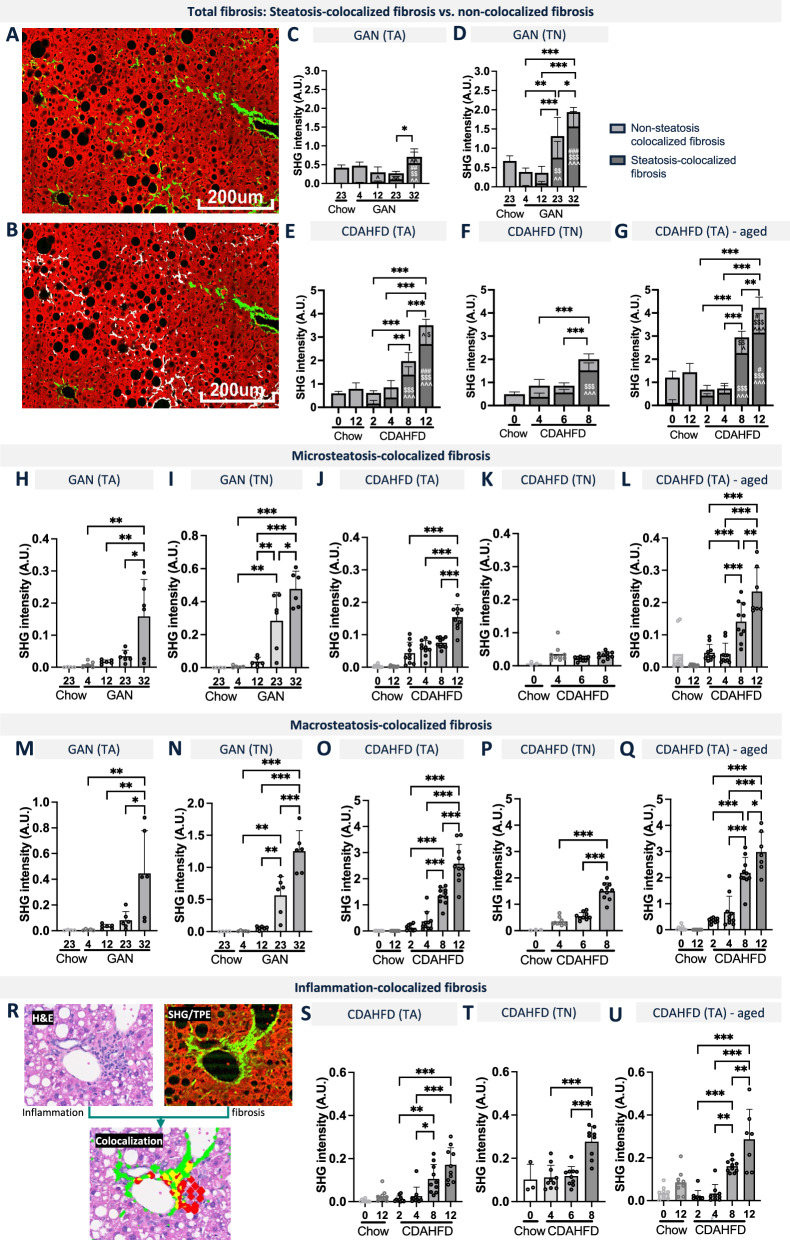
Colocalization analysis using AI-DP. **A** Representative SHG/TPEF image with fibrosis and (**B**) the detection of steatosis-colocalized fibrosis (white) and non-colocalized fibrosis (green). **C**–**G** Quantification of steatosis-colocalized fibrosis and non-colocalized fibrosis for different mouse (C57BL/6 J) models at thermos-ambient (TA) and thermos-neutral (TN) condition, (**H**–**L**) Quantification of fibrosis colocalizing with microsteatosis and (**M**–**Q**) macrosteatosis in different animal models, (**R**) Analysis approach for Inflammation/fibrosis colocalization as quantified using a combination of H&E and SHG image (green: fibrosis, red: inflammation, yellow: inflammation-colocalized fibrosis). **S**–**U** Quantification of inflammation-colocalized fibrosis in (**S**) CDAHFD at TA, (**T**) CDAHFD at TN, (**U**) CDAHFD-aged at TA. Data is shown as means ± SD; **C**, **H**, **M**: GAN *N* = 5-6 per timepoint, Chow *N* = 4; **D**, **I**, **N** CDAHFD *N* = 6–7 per timepoint, Chow TN *N* = 4 per timepoint; **E**, **J**, **O**, **S** CDAHFD, Chow *N* = 10 per timepoint; **F**, **K**, **P**, **T**: CDAHFD *N* = 10 per timepoint, Chow *N* = 3 per timepoint; **G**, **L**, **Q**, **U** CDAHFD *N* = 7–10 per timepoint, Chow *N* = 9–10 per timepoint. Statistical significance (one-way ANOVA, Bonferroni post-hoc analysis) was shown for comparisons within MASH diet group only, with symbols on top of the bars (*) indicate the comparisons for the full bars (overall liver), while those inside the bars indicate the comparisons for the corresponding parameters (^: comparison vs. the first timepoint, $: vs. the second timepoint, #: vs. the third timepoint, &: vs. the fourth timepoint in the diet group), the number of symbols indicates significance levels: 1: *P* < 0.05, 2: *P* < 0.01, 3: *P* < 0.001. Age: 7 weeks upon start of GAN diet (TA/TN) and CDAHFD (TN); 8 weeks upon start of CDAHFD (TA); 77 weeks upon start of CDAHFD (TA, Aged).

To assess whether liver fibrosis development in MASH models is metabolically driven, we quantified the colocalization of steatosis and fibrosis using SHG/TPEF images (Fig. 5A, B). A significant increase in steatosis-colocalized fibrosis was observed across different murine models. In the GAN-TA model, steatosis-associated fibrosis escalated by week-32, showing a 5-fold increase compared to week-23 (*P *= 0.009), while the week-23 level was not significantly different from week-4 (*P* > 0.99, Fig. 5C). Conversely, the GAN-TN model exhibited a notable increase in steatosis-associated fibrosis by week-23, with rises of 50-fold and 8.5-fold compared to week-4 (*P* = 0.003) and week-12 (*P* = 0.005), respectively (Fig. 5D). By week-32, GAN-TN showed 2.9-fold higher fibrosis than GAN-TA (*P* = 0.001). In the CDAHFD model, steatosis-associated fibrosis increased significantly by week-8 (3.3-fold at TA and 4.1-fold at TN vs. week-4, *P* < 0.001; Fig. 5E–G) and remained unaffected by housing temperature (P = 0.43).

The colocalization analysis of fibrosis adjacent to microsteatosis (Fig. 5H–L) or macrosteatosis (Fig. 5M–Q) demonstrates the critical importance of differentiating macrosteatosis and microsteatosis. In GAN model, time-dependent increase of the fibrosis colocalization with microsteatosis and macrosteatosis was accelerated and exacerbated at TN than at TA with the magnitude of colocalization with liver fibrosis being higher with macrosteatosis than with microsteatosis (Fig. 5H, I, M, N). In CDAHFD, temporal severity of disease manifestation remained similar between TA and TN perhaps owing to aggressive nature of the disease model, with more prominent time-dependent increase in fibrosis colocalized with macrosteatosis (Fig. 5J, K, O, P) suggesting macrosteatosis, rather than microsteatosis, being the driver of fibrosis in this model as well. In the more severe model of CDAHFD, aged CDAHFD at TA, there was a more intense time-dependent increase in microsteatosis and macro-steatosis colocalized fibrosis at week-8 (when compared with other models) with fibrosis colocalizing with macrosteatosis being consistently higher than that with microsteatosis (Fig. 5L, Q). These findings are in line with a previous study^26^ that showed qFibrosis co-localized with macrosteatosis generally correlated better with histological scoring and had a higher AUC in six animal models.

To further investigate non-colocalized fibrosis (fibrosis not colocalized with steatosis), we used an orthogonal approach and evaluated the inflammatory cellular environment surrounding collagen fibrils by co-registering H&E images with SHG/TPEF images (Fig. 5R). Inflammation-colocalized fibrosis significantly increased by week-8 (4.2-fold at TA, *P* = 0.02; 2.5-fold at TN, *P* = 0.002) in CDAHFD-fed mice, with a more pronounced response at TN (2.6-fold higher at TN vs. TA at week-8, *P* < 0.001; Fig. 5S-T). Although overall fibrosis development in the CDAHFD model was similar across temperature conditions (Fig. 4E, F), the data suggest that inflammation, plays a critical role in driving fibrosis under TN conditions (Fig. 5S, T).

### AI-DP for evaluation of pigmented granulomas and lipogranulomas in CDAHFD and GAN models of MASH and liver fibrosis

**Fig. 6:**
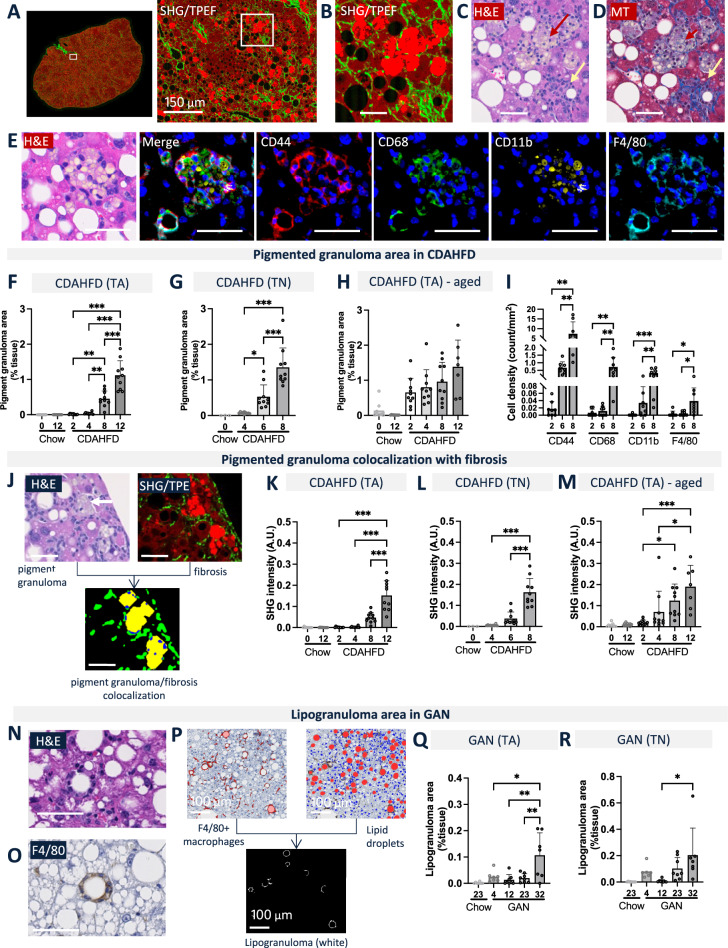
Pigmented granuloma and lipogranuloma evaluation using AI-DP. **A** Representative SHG/TPEF image of a CDAHFD mouse (C57BL/6 J) model, where autofluorescence from pigmented granuloma (foamy pigment laden cells) was observed. Zoomed area of the white box in A shown in (**B**) SHG/TPEF, (**C**) H&E, and (**D**) MT. **E** Multiplexed-IF for macrophage panel (CD44,CD68, CD11b, F4/80), along with the H&E on the same section, further demonstrates the presence of macrophages in the pigmented granuloma. Classical crown-like structure, indicating lipogranuloma, could also be observed (yellow arrows in **C**, **D**). **F**–**H** Quantification of the pigmented granuloma area in CDAHFD models, **I** Contribution of the different macrophages to the pigmented granuloma. **J** Pigment granuloma/fibrosis colocalization as quantified using a combination of H&E and SHG. **K**–**M** Quantification of pigment granuloma-colocalized fibrosis in the different animal models. **N**, **O** Classical lipogranuloma in GAN as shown in (**N**) H&E and (**O**) F4/80 (not on the same section), (**P**) quantification method for lipogranuloma, (**Q**, **R**) lipogranuloma quantification in GAN models. White bars on images indicate 50 µm unless indicated otherwise. Data is shown as means ± SD; **F**, **K**: CDAHFD *N* = 10 per timepoint, Chow *N* = 10 per timepoint; **G**, **L**: CDAHFD *N* = 10 per timepoint, Chow *N* = 3 per timepoint; **H**, **M** CDAHFD *N* = 7–10 per timepoint, Chow *N* = 9-10 per timepoint; I: *N* = 8 per timepoint; Q-R: GAN *N* = 7–8 per timepoint, Chow *N* = 8 per timepoint. Multiplexed-IF data: *N* = 8 per timepoint). Statistical significance (one-way ANOVA, Bonferroni post-hoc analysis) was shown for comparisons within MASH diet group only, with the level of significance: *P < 0.05, *P < 0.01, ***P < 0.001. Age: 7 weeks upon start of GAN diet (TA/TN) and CDAHFD (TN); 8 weeks upon start of CDAHFD (TA); 77 weeks upon start of CDAHFD (TA, Aged).

While imaging unstained liver tissue sections from CDAHFD-fed mice using SHG/TPEF, we detected tiny auto-fluorescent spots (Fig. 6A, B). Staining these sections with H&E (Fig. 6C) and Masson’s Trichrome (MT; Fig. 6D) revealed that the auto-fluorescent spots corresponded to foamy cells identified as macrophages in granulomatous lesions (red arrows in Fig. 6C, D), confirmed by 4-plex immunofluorescence for macrophage panel (Fig. 6E). A high correlation (Spearman’s R = 0.89, Supplementary Fig. 6A) was found between AI-based quantification of pigmented granuloma area and pathologist scoring of foamy cells.

We temporally evaluated pigmented granulomas using AI-DP (Fig. 6F, H), noting an increase in lesion area over time. In the CDAHFD model, factors such as age (2.1-fold higher in aged vs. young mice at week-8, *P* = 0.015; Fig. 6F, H) and housing conditions (3-fold higher at TN vs. TA at week-8 in age-matched young mice, *P* < 0.001; Fig. 6F, G) influenced granuloma growth. Pigment granuloma starts to correlate with inflammation after week-4 (Pearson’s R = 0.51, *P* = 0.016, Supplementary Fig. 6B), supporting the association with chronic inflammation^27^. We observed an increasing time-dependent prevalence of CD44+ cells in the pigmented granuloma, which was about 8-fold at week-8 compared to CD68+ cells as the second highest population within the pigmented granuloma (Fig. 6E, I). This supports previous reports that granulomas reflect ongoing lipid handling/stress and macrophage activation (specifically CD44+ and CD68 + ) within the liver^28,29^. Notably, pigmented granuloma prevalence correlated strongly with liver fibrosis (Pearson’s R = 0.75, *P* < 0.001, Supplementary Fig. 6C), which is supported by the colocalization analysis between pigmented granuloma and fibrosis (Fig. 6J–M).

In the GAN model, granulomatous lesions, or lipogranulomas, appeared morphologically distinct from those in the CDAHFD model. Lipogranulomas are characterized by steatotic hepatocytes or fat-droplets surrounded by mononuclear cells and macrophages^28^, resembling a crown-like structure (Fig. 6N, O). Quantification showed an overall increase in lipogranulomas over time with substantial variability, particularly at later time points (Fig. 6Q, R) likely reflecting lower fibrotic burden in the GAN model, and distinct biological-associations of lipogranulomas vs pigmented granulomas with fibrosis. These findings underscore the need to further optimize the machine-learning algorithm to improve detection sensitivity.

### Validation of AI-DP using clinically validated assets

**Fig. 7:**
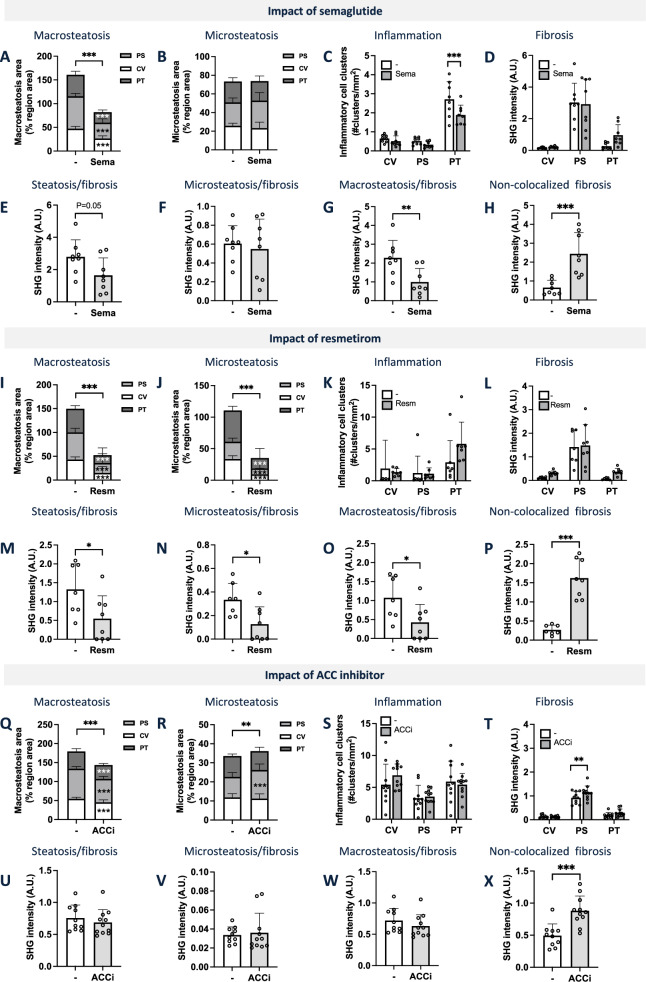
Understanding the impact of clinically relevant interventions on histological features using AI-DP. Impact of (**A**–**H**) Semaglutide, (**I**–**P**) Resmetirom, (**Q**–**X**) ACCi, on (**A**, **I**, **Q**) macrosteatosis, (**B**, **J**, **R**) microsteatosis, (**C**, **K**, **S**) inflammation, (**D**, **L**, **T**) fibrosis, (**E**, **M**, **U**) steatosis-colocalized fibrosis, (**F**, **N**, **V**) microsteatosis-colocalized fibrosis, (**G**, **O**, **W**) macrosteatosis-colocalized fibrosis, and (**H**, **P**, **X**) non-colocalized fibrosis. Data is shown as means ± SD; A-H:±Sema *n* = 8, GAN fed C57BL/6 J (Diet started at 7weeks of age); **I**, **J**, **L**, **M**–**P** Untreated (-) *N* = 7, Resm=8, **K** ±Resm *N* = 7 per timepoint, GAN fed C57BL/6 J (Diet started at 7 weeks of age); **Q**–**R**, **T**–**X**: Untreated (-) *N* = 10, ACCi=11; S: Untreated (-) *N* = 9, ACCi=11,CDAHFD fed C57BL/6 N (Diet started at 9-12 weeks of age). Statistics: **A**–**D**, **I**–**L**, **Q**–**T** within each zone comparison using uncorrected Fisher’s LSD; **E**–**H**, M-P, U-X: 2-sided Student’s T-test. Levels of statistical significance: *P < 0.05, *P < 0.01, ***P < 0.001.

To validate AI-DP-based detection, several clinically relevant pathways were assessed. Semaglutide, a GLP-1 receptor agonist, effectively reduced hepatic lipotoxic burden and improved MASH resolution^16,30^, as evidenced by significant improvements in NAS, steatosis, inflammation, and ballooning scores (Fig. 7). AI-DP showed a notable reduction in macrosteatosis (1.7- to 2-fold lower vs. vehicle, *P* < 0.001; Fig. 7A) with Semaglutide while no significant reduction in fibrosis across different zones with the treatment (Fig. 7D). Partitioning total fibrosis into regions colocalized with steatosis and non-colocalized regions showed semaglutide reduced steatosis-colocalized fibrosis (by 1.7-fold *P* = 0.05, Fig. 7E), which is mostly attributed to the reduction the colocalization with macrosteatosis rather than microsteatosis (Fig. 7F, G). This was supported by mRNA profiling of inflammatory and fibrotic genes, histological staining for fibrosis markers (α-SMA, galectin-3, Col1a1), and improvements in liver enzymes (ALT, AST), plasma lipid profiles, and body weight (Supplementary Fig. 7). These findings highlight the impact of macrosteatosis on liver fibrosis and treatment-mediated changes in disease etiology.

Resmetirom, a liver-directed thyroid hormone receptor beta agonist, also demonstrated significant reductions in steatosis and circulating lipid profiles (Supplementary Fig. 8) consistent with published reports^17^. AI-DP further revealed a ~ 3-fold reduction in macrosteatosis and 3.0- to 3.6-fold reduction in microsteatosis (*P* < 0.001; Fig. 7I, J). While there was no significant impact on total fibrosis (*P* = 0.221; Fig. 7L), partitioning total fibrosis into regions colocalized with steatosis and non colocalized regions reveals that resmetirom predominantly reduced fibrosis adjacent to steatotic areas by 2.4-fold (*P* = 0.036; Fig. 7M), for both macrosteatosis and microsteatosis (Fig. 7N, O). The apparent increase in the non colocalized fraction (*P* = 0.003; Fig. 7P) reflects the residual–an artifact of partitioning distribution within the unchanged total fibrosis signal rather than an absolute rise in collagen burden. This interpretation is supported by our cytokine profiling and mRNA analyses, which show no evidence of increased inflammatory or fibrotic activity in treated animals (Supplementary Fig. 8).

MK4074, a liver-specific Acetyl CoA Carboxylase (ACC1 and ACC2) inhibitor (ACCi) blocks malonyl-CoA mediated fatty acid synthesis and hence, reduces hepatic steatosis^18^. Treatment with ACCi significantly reduced macrosteatosis ( ~ 1.2-fold lower vs. untreated; Fig. 7Q) while increasing microsteatosis in PS (1.4-fold higher; Fig. 7R), suggesting a breakdown of macrosteatosis. AI-DP findings are aligned with conventional histopathology (Supplementary Fig. 9A) and previous reports^18,31^. ACCi did not show efficacy in reducing fibrosis, steatosis colocalized fibrosis or micro-/macro-steatosis colocalized fibrosis (Fig. 7T,U,V,W) in the CDAHFD model. Similarly, the apparent relative increase in the non-colocalized compartment with ACCi treatment (Fig. 7X) reflects a partitioning artifact rather than true induction of fibrogenesis, a conclusion supported by mRNA profiling of fibrogenic and inflammatory markers (Supplementary Fig. 9A). Improvement in steatosis while no effect on fibrosis (PSR) reduction with ACCi treatment was confirmed in the GAN model (Supplementary Fig.9B).While the use of a different mouse strain (C57BL6/N from Taconic) could affect model progression and drug responsiveness (Fig. 7, and Supplementary Fig. 9A), and based on the data presented, 10-weeks on the GAN diet (Supplementary Fig. 9B) is premature for robust fibrosis development. So, the apparent increase in fibrosis in PS (Fig. 7T) or lack of fibrosis reduction (Supplementary Fig. 9B) likely reflects a suboptimal timepoint for fibrosis assessment. Mechanistically, ACC inhibition may be more relevant to earlier stages of disease. In addition, prolonged ACCi treatment may have deleterious effects, including plasma hypertriglyceridemia^18^ or elevated PNPLA3 mRNA^32^. For these reasons, the present study was designed to evaluate the impact of ACCi on steatosis, which was robustly captured using AI-driven DP.

## Discussion

Identifying the optimal murine model for MASH is crucial for understanding disease mechanisms, evaluating therapies, and translating research into clinical applications. An effective model should closely mimic human MASH pathophysiology, including liver steatosis, inflammation, and fibrosis, to facilitate studies on disease progression and the multifactorial influences of genetics and environment. Accurate models also provide insights into the efficacy and safety of potential therapies, helping to identify biomarkers, predict treatment outcomes, and reduce clinical trial failures. In summary, selecting a “fit-for-purpose” murine model is essential for advancing MASH research and developing effective prevention and treatment strategies.

In this study, we thoroughly characterized diet-induced male murine models of MASH and liver fibrosis using AI-driven DP to analyze macro- and micro-steatosis features, inflammatory cell density/clusters, and fibrosis morphometrics across the liver zones, supported by initial assessment via bulk RNA sequencing, lipidomics, metabolomics, standard blood chemistry, and conventional histopathology. Male mice develop diet-induced MASLD/MASH and liver fibrosis more rapidly and severely than females, which are partially protected by estrogen^33,34^. As the primary aim was to demonstrate AI-enabled DP rather than examine sexual dimorphism, we prioritized the sex with the most robust disease phenotype.

The specific novelty of our approach is the integration of the multi-modal AI-DP, enabling colocalization analysis between different histopathological features in MASH, such as steatosis (macro and micro)-fibrosis, inflammation-fibrosis, or lipogranuloma-fibrosis colocalization. This enables detailed assessment of inflammation and dysmetabolism in liver fibrosis progression across different zones, thereby deepening our understanding of the disease’s etiology. GAN and CDAHFD emerged as primary models for metabolically induced liver injury and fibrosis, respectively, aligning with existing literature. In the GAN model, colocalization of steatosis and fibrosis indicated dysmetabolism-driven fibrogenesis, consistent with clinical observations^5^. On the other hand, inflammation peaked at week-8 and coincided with fibrotic regions, implicating inflammation as a key driver in the CDAHFD model. Overall, AI-based analysis pinpoints critical time windows to evaluate anti-steatotic versus anti-inflammatory therapies in pharmacological studies.

Histopathology in murine MASH models showed accelerated disease progression under TN conditions (vs TA), consistent with prior reports^35^. Mice fed on GAN diet reached Grade-3 steatosis by 4-weeks at TN versus 8-weeks at TA, with higher inflammation and ballooning scores at TN consistent with the upregulation of genes associated with hepatic fibrosis and inflammation, increase in hepatic CE and short-chain, highly saturated TAGs, and reduction in phospholipid levels.

AI-enabled DP clarified zonal patterns of steatosis. In human liver, triglyceride synthesis pathways are enriched pericentrally (Zone-3) and least periportally (Zone-1)^36^. Further, in human MASH, accumulation of fat in hepatocytes follows four distribution patterns: panacinar, zone-1 (periportal), zone-3 (centrilobular or pericentral), and azonal^37^. In both GAN and CDAHFD models, the distribution appears to be panacinar with distinct distribution of microsteatosis vs. macrosteatosis in GAN and CDAHFD. In GAN, microsteatosis emerged earlier and predominated pericentrally and macrosteatosis periportally, a pattern evident early and maintained through 32-weeks (Supplementary Fig. 2), consistent with metabolic zonation^36^. In CDAHFD, macrosteatosis dominated all zones as early as 4-weeks, likely due to choline deficiency reducing phosphocholine related lipid-droplet surface-tension leading to lipid-droplet coalescence to form macrosteatosis^23^. These zone-resolved differences show how model-specific drivers shape steatosis and may diverge from human disease. Our data provides biologically coherent AI-derived micro/macro classifications: microsteatotic regions aligned with lipidomic/metabolomic signatures of de novo lipogenesis and fatty-acid remodeling, whereas macrosteatosis colocalized more strongly with fibrosis, indicating pathologically relevant heterogeneity.

Further, AI-enabled analysis revealed temporal changes in fibrosis severity and features, showing how escalating dysmetabolism and inflammation drive progression, an insight beyond conventional histopathology. A notable morphometric feature was the differential manifestation of granulomas: in GAN model, crown-shaped lipogranulomas with macrophages encircling lipid-droplets were present, while in the CDAHFD model, foamy, pigmented lesions known as pigmented granulomas were observed. These reflect lipid-trafficking dysfunction and macrophage activation (predominantly CD44 + /CD68 + ), and their emergence correlated with fibrosis progression. Prior reports suggest lipogranulomas may promote liver cancer even without cirrhosis^28^, but their prognostic significance is context-dependent—shaped by diet, injury-duration, iron-load or oxidative-stress, and the fibrogenic response—and granulomas can regress if the insult is removed. Given their temporal heterogeneity, granulomas are unsuitable as standalone staging metrics. To strengthen causal inference, therapeutic relevance, and prognostic utility, future work should integrate AI-based segmentation with spatial omics or laser-capture microdissection, and conduct longitudinal intervention studies to define the role of granulomas in tissue remodeling and disease etiology, and determine whether interventions differentially reverse micro- versus macrosteatosis and their associated inflammatory and fibrogenic pathways.

With a solid understanding of disease progression established, we evaluated several clinically relevant pathways. All three drugs—Semaglutide, Resmetirom, and ACCi—significantly reduced macrosteatosis across all tested zones. Resmetirom was most effective in reducing microsteatosis, followed by ACCi, underscoring the importance of selective thyroid hormone β-receptor agonism in alleviating lipotoxicity. Both Semaglutide and Resmetirom significantly reduced steatosis-colocalized fibrosis, while ACCi showed no efficacy. This lack of effect may be due to testing at a suboptimal time point, as fibrosis may not have developed sufficiently to observe meaningful changes. The efficacy of these treatments was supported by conventional histopathology, plasma lipid-profile assessments, and improvements in inflammation and fibrosis through mRNA-profiling.

AI-enabled DP offers several major advantages that complement expert evaluation. First, it enables objective, quantitative, and fully reproducible scoring, thereby reducing inter- and intra-observer variability. Second, once trained, the algorithm can analyze whole-slide images within minutes, allowing efficient, high-throughput evaluation of large experimental cohorts that would otherwise require extensive pathologist time. Third, AI models can detect subtle and spatially heterogeneous histological changes that may escape semi-quantitative manual scoring, thus improving sensitivity for early or partial treatment effects.

AI-enabled DP has also increasingly been applied to human MASH, where liver biopsy remains the diagnostic and treatment evaluation gold standard. Platforms such as AIM-MASH^38^, FibroNest^39^, and HistoIndex^40^ provide continuous, quantitative scores of histopathologic features, addressing limitations of conventional semiquantitative scoring, with each platform having distinct capabilities^4^. In our workflow, we combined HistoIndex SHG-based AI for sensitive fibrosis detection with an in-house H&E-based AI pipeline for features better resolved on H&E (e.g., inflammation, granulomas, and liver zonation). We then registered SHG and H&E modalities to enable zone-specific and colocalization analyses. Although optimized for mouse models, our approach is translatable to human-specimens or other experimental-models, with appropriate finetuning to account for species-specific histopathologic differences.

Integrating AI-enabled DP into MASH murine studies is promising but has limitations. Small cohorts risk overfitting and spurious detections when pathology distributions shift across models. Threshold selection and thus, true/false positive trade-offs depend on the validation set, underscoring the need for careful dataset-curation, alignment between development and target model distributions, and ongoing finetuning for deployment across diverse samples. Although AI reliably detects salient histologic landmarks, biomarker choice remains critical for clinical translatability, necessitating cautious interpretation and orthogonal validation. Additionally, it would certainly be valuable to include data from both males and females in future studies to broaden the applicability of the findings.

In conclusion, AI-driven DP enhances MASH research by improving diagnostic precision and efficiency, enabling timely monitoring of steatosis and fibrosis, and facilitating selection of fit-for-purpose murine models to probe mechanisms and test therapies. Collectively, these advances position AI to accelerate translational research and with appropriate human validation, inform clinical decision-making in liver disease, with the ultimate goal of improving patient outcomes.

## Methods

### Animals

#### Ethics statement

All animal experiments were approved and were performed in compliance with the Institutional Animal Care and Use Committee (IACUC) of Merck & Co., Inc., Rahway, NJ, USA (APS400180) and conducted in accordance with institutional and national guidelines for animal welfare.

C57BL/6 J mice (7–8 weeks old; aged cohort, 77 weeks) and ob/ob mice (B6.Cg-Lepob/J) were obtained from The Jackson Laboratory. For the ACC inhibitor intervention study in the CDAHFD model, C57BL/6 N wild-type mice (9–12 weeks old) were obtained from Taconic. Animals were housed under a 12-h light/12-h dark cycle (lights on at 06:00) in a temperature-controlled environment maintained at either thermoneutrality (29 ± 1 °C) or standard ambient temperature (21 ± 1 °C). Male mice were used for all the experiments because they develop diet-induced MASLD/MASH and liver fibrosis faster than estrogen-protected females, maximizing the disease window for AI-enabled DP development. Each animal was identified by an ear punch that was performed after the animal had acclimated for a week. Mice had ad libitum access to water and food (normal chow or NC LabDiet5001; GAN D09100310, Research Diets; CDAHFD A06071302, Research Diets; FDSW **F**riedmann **D**iet TD.120528 + **S**ugar **W**ater (23.1 g/L d-fructose & 18.9 g/L d-glucose). For the murine model with Carbon Tetra Chloride (CCl4), CCl4 (cat#289116-100 ml; Sigma) was dosed 0.2ul (0.32ug / g BW) once a week intraperitoneally. To evaluate the effect of treatment, a cohort of GAN and CDAHFD-fed animals were treated with Semaglutide at 30nmol/kg s.c. QD, Resmetirom at 3mpk ad-lib (in-feed), or ACCi (MK4074) at 30mpk (in-feed). Animals were terminated by cardiac puncture under isoflurane anaesthesia.

Animals (*N* = 8-10 per condition) were randomized based on body weight (BW) and baseline blood chemistry; and sampling for this purpose was performed via tail snip and/or submandibular bleeds after a 5 hour fast in compliance with the IACUC regulations. Mice were kept on different diets for different durations (up to 32-weeks on GAN, up to 23-weeks on FDSW, up to 16-weeks on CDAHFD) and were necropsied after a 5 hour fast to collect tissues including liver and blood. After 5 hours of fasting, intra‑hepatocyte glycogen was unremarkable to only mild/moderate level, minimizing ambiguity of cytoplasmic clearing due to glycogen vs. lipid-droplets.

### Histopathology

Livers from the euthanized mice were placed in 10% Neutral-Buffered Formalin for 24 hours^41^ followed by transferring them to 70% EtOH at room temperature. The left lateral lobes of the livers were embedded in paraffin blocks and 5 μm thickness liver slides per sample were prepared. For pathologist evaluation, two slides were prepared: one slide was stained with Hematoxylin and Eosin (H&E) using the standard protocol, and the other slide was stained with picrosirius red stain (PSR) using the staining kit purchased from American MasterTech (StatLab). All stained slides were examined by light microscopy and digitally scanned at 20× using NanoZoomer 2.0HT slide scanner (Hamamatsu Photonics) for conventional histological scoring by pathologist. PSR was analysed using ORBIT image analysis software at 20x.

Another slide was prepared for Second Harmonic Generation/Two Photon Excitation (SHG-TPE) scanning (HistoIndex, Singapore), followed by H&E staining, on which the multi-modal integrated AI-DP pipeline was built. The H&E slides were then scanned at 40x using Vectra Polaris (Akoya Biosciences) to obtain whole slide images (WSIs). For the evaluation of pigment granuloma and lipogranuloma, F4/80 immunohistochemistry (IHC) was performed using rabbit anti-mouse F4/80 (ab6640, Abcam) using a standard protocol.

Histopathological evaluation including assessment of cellular and sub-cellular features from tissue sections was performed by board-certified pathologists Y.Z. (from Histobridge) and R.R. (from AMPL).

### Multiplexed-Immunofluorescence (mIF)

In a subset of animals, mIF was performed using Mouse U-VUE customized 4-plex (Ultivue) on two sections: one section for a T-cell panel (CD3, CD4, CD8, and FoxP3; Catalogue #ULT30425), and one section for a macrophage panel (F4/80, CD11b, CD68, and CD44; Catalogue #ULT30427-M). All samples underwent an antigen retrieval and dewax protocol prior to a blocking stop. Following this, the pre-optimized DNA-barcoded primary antibodies, diluted 1:100 following the manufacturer’s protocol, co-incubated on the tissue. Signal amplification was then performed simultaneously for all targets to increase assay sensitivity. Spectrally distinct fluorescent oligonucleotides complementary to the barcode were then added to label each antibody conjugate prior to imaging. Following the mIF, the slides were then scanned for WSI using VectraPolaris (Akoya Biosciences) at 40x, 5-channels (FITC, TRITC, CY5, CY7, including DAPI). Subsequently, the slides were washed and re-stained for H&E and scanned for WSI at 40x brightfield using VectraPolaris (Akoya Biosciences). Each marker in the mIF images were analyzed using Nuclei Seg and HighPlex FL v4.2.14 modules in HALO-AI (IndicaLabs).

### Pathologist scoring

Pathologists evaluated steatosis (score: 0-3), hepatocyte ballooning (score: 0-2), lobular inflammation (score: 0-3), and fibrosis (score: 0-4), using H&E and PSR liver slides or WSIs, following the NASH-CRN scoring system, in the same paradigm as how the system is applied in human^42^. NAFLD activity score (NAS) was calculated as a sum of steatosis, hepatocyte ballooning, and lobular inflammation score. In addition, foamy pigment laden cells were also evaluated and assigned score 0-3 (0: no foci, 1: mild, 2: moderate, 3: severe). For validation of AI model, steatosis, inflammation, and fibrosis score were correlated with AI-DP-derived steatosis area, inflammatory cell density, and collagen intensity.

### Blood chemistry

Blood chemistry involved evaluating glucose from whole blood, and liver enzymes, lipid profile and insulin from plasma after a 5 hour fast. Glucose was measured via tail snip using a glucometer (AlphaTrak2), insulin was measured using the Mercodia Insulin ELISA kit (cat#10-1247-10), and remaining parameters were measured using the COBAS C311 Clinical Analyzer.

### RNA isolation and quantitative real-time PCR (RT–qPCR)

mRNA profiling for inflammatory and fibro genic genes upon treatment with clinically validated therapeutics involved Total RNA isolation from mouse liver tissue using the RNeasy Mini Kit (Qiagen; cat. no. 74116) on a QIAcube Connect automated platform (Qiagen), following the manufacturer’s instructions. Briefly, liver tissue was homogenized in QIAzol lysis reagent (Qiagen; cat. no. 79306) using Lysing Matrix tubes (Next Advance, NAVYR1-RNA), followed by phase separation with chloroform. The aqueous phase was processed on the QIAcube Connect for RNA purification. Genomic DNA contamination was removed using an RNase-free DNase kit (Qiagen; cat. no. 79256). RNA concentration and purity were assessed using a NanoDrop 8000 spectrophotometer (Thermo Fisher Scientific). Complementary DNA (cDNA) was synthesized from 500 ng total RNA in a 20 µL reaction volume using the SuperScript™ VILO™ cDNA Synthesis Kit (Thermo Fisher Scientific; cat. no. 11754050) according to the manufacturer’s protocol, using a thermal cycler. Quantitative PCR was performed using TaqMan™ Gene Expression Assays from Thermofisher Scientific (summarized in the table below) on a ViiA™ 7 Real-Time PCR System (Applied Biosystems). Reactions were set up using TaqMan Universal PCR Master Mix and gene-specific hydrolysis probes. Fluorescence signals were acquired during amplification, and threshold cycle (Ct) values were determined using instrument software.

Relative mRNA expression levels were calculated using the comparative Ct (ΔΔCt) method and normalized to mouse TBP housekeeping gene.

**Table:** 

Gene	Taqman assay ID	Dye
TBP	Mm01277042_m1	VIC-MGB_PL
ACTA2	Mm00725412_s1	FAM-MGB
ADGRE1	Mm00802529_m1	FAM-MGB
CCL2	Mm00441242_m1	FAM-MGB
CCR2	Mm99999051_Gh	FAM-MGB
CCR5	Mm01963251_s1	FAM-MGB
CD68	Mm03047343_m1	FAM-MGB
COL1A2	Mm00483888_m1	FAM-MGB
COL3A1	Mm00802300_m1	FAM-MGB
COL4A2	Mm00802386_m1	FAM-MGB
IL1B	Mm00434228_m1	FAM-MGB
LGALS1	Mm00839408_g1	FAM-MGB
SAA1	Mm00656927_g1	FAM-MGB
TGFB2	mm00436955_m1	FAM-MGB
TIMP1	mm01341361_m1	FAM-MGB
TIMP2	Mm00441825_m1	FAM-MGB
TNF	Mm00443258_m1	FAM-MGB
VIM	Mm01333430_m1	FAM-MGB

### Multiplex cytokine analysis (MSD platform)

Cytokine concentrations in plasma of mouse treated with Resmetirom were measured using the MSD Proinflammatory Panel (cat. no. C4048-1) and MSD Cytokine Panel (cat. no. C4245-1) (Meso Scale Discovery) according to the manufacturer’s instructions. Electrochemiluminescence signals were acquired on a MESO SECTOR S 600 instrument and cytokine concentrations were determined from standard curves using a 4-parameter logistic fit.

### Murine transcriptomics datasets

*RNA isolation* – Liver tissues were homogenized into RNA STAT-60 (Tel-Test Inc., Friendswood, TX) using a polytron homogenizer, followed by total RNA isolation using the MagMAX mirVana Total RNA isolation kit (Thermo Fisher Scientific Inc., Foster City, CA) according to the manufacturer’s instructions. Total RNA samples were qualified and quantified on the Fragment Analyzer (Agilent Technologies, Santa Clara, CA) per manufacturer’s instructions.

*NGS Library Preparation and Enrichment –*For the TruSeq Stranded mRNA protocol, total RNA was utilized as the input for Illumina’s TruSeq Stranded mRNA library preparation kit (Platform: Illumina NovaSeq6000). This method purified polyA-containing mRNA molecules using oligo-dT attached magnetic beads and involved two rounds of purification. During the second elution of the polyA RNA, the RNA was fragmented and primed for complementary DNA (cDNA) synthesis. Subsequently, the cleaved RNA fragments, primed with random hexamers, were reverse transcribed into first-strand cDNA. The RNA template was then removed, and a replacement strand was synthesized, incorporating dUTP in place of dTTP to generate double-stranded cDNA (ds cDNA). An adenine (A) nucleotide was added to the 3ʹ ends of the blunt fragments to prevent them from ligating to one another during the adapter ligation reaction. Multiple indexing adapters were ligated to the ends of the ds cDNA fragments in preparation for hybridization. Polymerase chain reaction (PCR) was employed to selectively enrich those DNA fragments that possessed adapter molecules on both ends and to amplify the quantity of DNA in the library. After amplification, the targeted libraries were quantified using quantitative PCR (qPCR) and pooled into a single lane. The pooled libraries were subsequently prepared for cluster generation and sequencing on high-throughput Illumina NovaSeq instruments. At least 4 gigabases of 50 base pairs of paired-end data were generated by the sequencing machine, in accordance with the project’s raw data requirements.

All Omics data was analyzed using R. For transcriptomics data, differential express analysis was performed with DESeq2, using the cutoff of log2(FC) > 1 or log2(FC) < -1, and Padj<0.05. For lipidomics data, differential altered lipids were identified with R package of lipidr, using the cutoff of log2(FC) > 2 or log2(FC) < -2, and Padj<0.05. For metabolomics data, differential altered metabolites were identified with R packages of omu and MetaboAnalystR, using the cutoff of log2(FC) > 2 or log2(FC) < -2, and Padj<0.05. All the 3 multiple test corrections were using Benjamini-Hochberg (BH) approach.

### AI-enabled multi-modal DP

AI-driven AI-DP pipeline was developed for detection of liver zones as well as quantification of steatosis, inflammation, and fibrosis, on unstained SHG/TPEF images, H&E-stained images, or IHC-stained images, which also includes co-registration modules for colocalization analysis between features (Fig. 1).

*SHG-TPE scanning & AI analysis of steatosis and fibrosis –*Unstained formalin-fixed paraffin-embedded (FFPE) tissue slides (section thickness: 5 µm) were scanned using the Genesis® 200 system (HistoIndex), which uses an ultrafast femtosecond laser, emitting photons to excite the unstained tissue sample at 780 nm. Second Harmonic Generation (SHG) signals at 390 nm and Two-Photon Excited Fluorescence (TPEF) signals at 550 nm were then collected at two photomultiplier tubes (PMT)^43,44^ signals were colorized and subsequently merged to form SHG/TPEF image, giving a resolution of 0.39 µm/pixel. TPEF signals (red in color) provide visualization of background liver architecture while SHG signals (green color) identify collagen fibres. AI/ML algorithm as implemented and validated in human samples^40^ was applied for mouse samples for identification of liver zone (Zone 3/CV, Zone 2/PS, Zone 1/PT) based on CV and PT landmarks, along with steatosis and fibrosis. Image analysis was then applied to quantify zone-specific features comprising a total of 100 fibrosis features and 45 steatosis features (Table S3–S4)^26^, as well as the colocalization between fibrosis and steatosis defined by fibrosis present within 14 μm around the fat vacuoles^5^. For mouse application, the CV/PT classification model was performed on 1081 lumen structures from 9 animal liver slides, resulted an accuracy of 0.81 (CV-Precision 0.82, CV-Recall 0.92, PT-Precision 0.81 and PT-Recall 0.61). The validation using the animal models in the current study (*N* = 256 liver slides) showed that fibrosis signal generated by SHG correlate with the standard PSR area quantification (Pearson’s R = 0.92, Supplementary Fig. 10G) and pathologists’ NASH-CRN fibrosis scores (Spearman’s R = 0.72, Supplementary Fig. 10H), while steatosis detection from the TPEF correlate with the pathologists’ steatosis score (Spearman’s R = 0.80; Supplementary Fig. 10I).

*Liver zone detection –*Liver zone (Zone 3/CV, Zone 2/PS, Zone 1/PT) on the H&E image was determined via AI-enabled detection of CV and portal vein (PV). The classification of CV and PV was performed in two stages: liver vein segmentation, followed by classification of each detected vein into portal vein or central vein. In the first stage we divided the tissue area of each H&E-stained WSIs into patches of size 512 ×512 with 50% overlap at 10x resolution and normalized them using Macenko method^45^. We then deployed a U-Net model^46^ with ResNet-50^47^ backbone to segment all veins found in these patches and combined overlapping veins together. In the second stage, we extracted the region within the bounding box of each vein dilated by 100 µm and deployed an Attention-based Deep Multiple Instance Learning^48^ approach with ResNet-18 backbone to classify each vein. The AI model was trained on 32,993 annotated veins, covering almost 175,000 training patches. The model was validated on annotated set of 670 patches from 67 independent samples covering 2 out of the 4 MASH animal models. The vein segmentation model achieves an average Intersection over Union (IoU) of 0.84 (Supplementary Fig. 11A, B), while the vein classification model achieves a balanced accuracy of 90.3% with an area under the curve of the Receiver-Operating curve (ROC AUC) of 0.95 and Precision-Recall curve (PR AUC) of 0.92 (Supplementary Fig. 11C, D). For zone-specific quantification, the detected zone served as a region of interest and was colocalized with the features of interest.

*Lipid-droplet quantification –* Individual lipid-droplet on the H&E images was detected using Vacuole 2.2 algorithm within HALO software (Indica Labs). The algorithm was fine-tuned separately for lipid-droplets with diameter smaller than 15 µm (classified as microsteatosis) and larger or equal to 15 µm (classified as macrosteatosis), with a circularity factor of 0.8. The 15 µm threshold was determined based on previous report^49^, as well as empirical and data-driven observations (Figure S12).

*Inflammatory cell detection –* Inflammatory cells were detected and quantified on the H&E images using a detection pipeline that we developed inhouse. Firstly, to train the inflammatory cell classification model, we collected inflammatory cell annotations from an expert veterinary pathologist. To minimize the time taken to annotate each individual cell, we only collected centroid annotations and use them as prompts to the Segment Anything Model (SAM)^50^ where we froze the model backbone and finetuned a linear classification head on the extracted features. In total, we trained our model on 161 patches of size 526 × 526 at 40x magnification comprising 10176 cells, for which 4398 are inflammatory cells, on both steatotic as well as non-steatotic areas. We trained the model on 42 independent patches containing 1123 non-inflammatory cells and 1084 inflammatory cells. The models achieve ROC AUC value of 0.92 and PR AUC value of 0.93. In the inference step, we split input H&E images into patches of size 526 × 526 for images that were acquired at 40x resolution and 256 × 256 for images that were acquired at 20x resolution. Firstly, we ran cell detection using StarDist^51^ (probability threshold at 0.8 and Non-Maximum Suppression (NMS) threshold at 0.1), extracted the centroid of each detected nucleus, and afterwards, we use them as prompts for the SAM model that will classify each given nucleus as inflammatory cell or non-inflammatory cell (probability threshold at 0.3). The StarDist and SAM thresholds were selected to balance the number of cells detected and false positive rate (Table S5). We then calculated the density of the inflammatory cells (i.e., the number of inflammatory cells per unit area) by counting the number of detected inflammatory cells divided by the regions of interest they colocalize to. We also quantified the number of inflammatory cluster normalized to the tissue area via DBscan clustering (minimum number of cells = 5, maximum distance between neighbouring cells = 15 µm), similar to pathologist’s evaluation of lobular inflammation. Inflammatory cell density and clusters showed a strong correlation with pathologist inflammation scores (Spearman’s R = 0.82 for inflammatory cell density and Spearman’s R = 0.84 for inflammatory cell foci, *P* < 0.001; Supplementary Fig. 4A, B). To assess the colocalization between inflammatory cells and fibrosis, the H&E images were registered to the corresponding SHG/TPEF images. The amount of fibrosis in the vicinity of inflammatory cells (10 µm) was quantified. The model should be interpreted as a relative, not absolute, quantification tool.

*Pigment granuloma quantification –*The detection^52,53^ for pigment granuloma (“foamy pigment laden cells”) on the H&E images was developed using Random Forest classifier within HALO software (Indica Lab), finetuned to detect pigment granuloma from the liver WSIs. The AI detection was highly correlated with pathologists’ scoring of foamy pigment laden cells (Spearman’s R = 0.89, *P* < 0.001, Supplementary Fig. 6).

*Lipogranuloma quantification* – We first detected F4/80-positive area from the F4/80 IHC images using HALO (Indica Labs), followed by lipid-droplet detection from the same IHC images using a similar approach described above for lipid-droplet detection on H&E images. We then colocalized the F4/80-positive area with the detected lipid-droplets followed by binary erosion to clean up smaller F4/80-positive surrounding the lipid-droplets and normalized the detected area by the total tissue area.

### Use of AI-generated content

A background illustration included in the graphical abstract was generated using Microsoft M365 Co-pilot (GPT-5 based large language and image generation model) based on text prompts designed by the authors and subsequently refined manually. This element is purely illustrative and does not represent experimental data. All scientific content and data shown in the figure are derived from experimental results generated and validated by the authors.

### Statistical analysis

For the evaluation of animal model development at multiple time points, statistical analysis was performed on the corresponding MASH diet group using one-way ANOVA, with Bonferroni post-hoc analysis. For the intervention study, statistical analysis was performed using Student’s T-test. Level of significance was set at *P* < 0.05. Data was presented as mean ± standard deviation (SD).

### Reporting summary

Further information on research design is available in the Nature Portfolio Reporting Summary linked to this article.

## Supplementary information

Supplementary Information

Reporting summary

Transparent Peer Review file

## Source data

Source Data

## Data Availability

Source data underlying all main and Supplementary Figs. and tables, including processed metabolite and lipid abundance values, are provided in the Source Data Files as Microsoft Excel files. All statistical results are provided in Microsoft Excel format in the Supplementary Information. Representative whole-slide images (WSIs) used to develop and evaluate the algorithms, as well as the intermediate files, are available via a Zenodo repository (10.5281/zenodo.18208001). The full set of WSIs underlying this study comprises histological data from 256 animals across multiple experimental conditions, with paired H&E and SHG images totally approximately 3–4 GB per animal (overall ~750 GB–1 TB). Owing to the size of this dataset and the associated storage and transfer constrains of general-purpose public repositories, the complete WSI dataset is not hosted online. The full dataset will be made available upon reasonable request to the corresponding author(s). Bulk RNA-seq data discussed in this publication have been deposited in NCBI’s Gene Expression Omnibus (GEO) and are accessible through GEO Series accession number GSE316023. Lipidomic and metabolomic profiling was performed by a third-party facility (Metabolon Inc.) under a commercial service agreement. All processed lipidomic and metabolomic data underlying the analyses and figures reported in the Supplementary Information are provided as Microsoft Excel files. Source data are provided with this paper.

## Code availability

All code for AI model deployment, downstream image processing, and analysis used in this manuscript is openly available at (10.5281/zenodo.18208001). The deposited materials include the code for zone detection, inflammatory cell detection, collagen and steatosis/inflammation/zonation mask extraction, co-localization analysis, representative input SHG and H&E images, and intermediate outputs for each processing step so readers can reproduce the full analytical workflow and apply the pipeline presented here. Processed output data for all samples are included in the Supplementary Materials. HistoIndex Pte. Ltd. proprietary software was used only to generate certain SHG-derived steatosis and fibrosis features and therefore those specific derived-feature implementations cannot be fully disclosed; all other components of the pipeline were developed in-house. The full raw image datasets and trained model checkpoints can be made available upon reasonable request.
