# Supplementary material for: AI digital pathology as a key tool providing in-depth understanding of the progression and regression of MASH and fibrosis in male mouse models
Source: Nat Commun. 2026 Jul 22;17:6964. doi: 10.1038/s41467-026-73370-z (PMC13392390; doi:10.1038/s41467-026-73370-z)
Supplement: Supplementary file 1 — Supplementary Information [file 41467_2026_73370_MOESM1_ESM.pdf]

AI digital pathology as a key tool providing in-depth understanding of the progression and regression of MASH and fibrosis in male mouse models

SUPPLEMENTARY INFORMATION

INVENTORY OF SUPPLEMENTARY INFORMATION

1. SUPPLEMENTARY FIGURES

a. Supplementary Fig. 1. | Multi-omics analysis of GAN models.
   i. Supplementary Fig. 1 A. | Comparison of NASH progression between GAN diet and normal chow (NC)–fed mice (C57BL/6J) using differentially expressed (DE) genes identified by bulk RNA sequencing.
   ii. Supplementary Fig. 1 B. | Pathway Enrichment analysis evaluating the effect of housing temperature in GAN fed mice (C57BL/6J).
   iii. Supplementary Fig. 1 C. | Enrichment analysis of lipidomic data in GAN and normal chow diet fed mice (C57BL/6J).
   iv. Supplementary Fig. 1 D. | TAG Fatty Acid Chain Saturation analysis in GAN and normal chow diet fed mice (C57BL/6J) at TA and TN.
   v. Supplementary Fig. 1 E. | Heatmap of metabolite abundance by subclass in GAN and normal chow diet fed mice (C57BL/6J).
   vi. Supplementary Fig. 1 F. | Summary of metabolomic changes by class in GAN and normal chow diet fed mice (C57BL/6J).

b. Supplementary Fig. 2. | Representative H&E images for progression in GAN (C57BL/6J).
c. Supplementary Fig. 3. | Representative H&E images for progression in CDAHFD model (C57BL/6N) at TN.
d. Supplementary Fig. 4. | Validation of AI model for inflammatory cell detection.
e. Supplementary Fig. 5. NASH-CRN inflammation scores as evaluated by pathologists for different animal models (C57BL/6J).
f. Supplementary Fig. 6. | Pigmented granuloma.
g. Supplementary Fig. 7. | The effects of GLP1 agonism (Semaglutide) on GAN diet induced steatosis, inflammation and fibrosis in male mice (C57BL/6J).
h. Supplementary Fig. 8. | The effects of THRβ agonism (Resmetirom) on GAN diet induced steatosis, inflammation and fibrosis in male mice (C57BL/6J).
i. Supplementary Fig. 9A. | The effects of ACC1/2 inhibition on CDAHFD induced steatosis, inflammation and fibrosis in male mice (C57BL/6N)
j. Supplementary Fig. 9B. | The effects of ACC1/2 inhibition on GAN diet induced steatosis, inflammation and fibrosis in male mice (C57BL/6J).
k. Supplementary Fig. 10. | Comparisons SHG/TPEF with conventional histopathology for fibrosis and steatosis.
l. Supplementary Fig. 11. | Performance of the deep learning model for vein segmentation and classification.
m. Supplementary Fig. 12. Determination of macrosteatosis and microsteatosis on H&E image.

2. SUPPLEMENTARY TABLES

a. Supplementary Table 1. | Summary of blood chemistry across different murine models of MASH/MASLD
b. Supplementary Table 2. | Differential gene expression analysis between GAN with normal diet under each temperature separately
c. Supplementary Table 3. | Categorization of steatosis features derived from SHG/TPEF images
d. Supplementary Table 4. | Categorization of fibrosis features derived from SHG/TPEF images
e. Supplementary Table 5. | The relationship between the stringency of the parameter set for the inflammatory cell detection model, number of cells detected as well as false positive rate (FPR).

3. STATISTICAL ANALYSIS RESULTS : Detailed statistical outputs, including test statistics, and exact p-values for all reported analysis

Tables

Supplementary Table 1. Summary of blood chemistry across different murine models of MASH/MASLD

Housing Temp	Animal Model	ALT (U/L)	AST (U/L)	tCHOL (mg/dL)
TA	*CDAHFD*	257.17 ± 62.42 (wk16)	280.93 ± 63.65 (wk16)	30.61 ± 3.29 (wk16)
	CDAHFD (Aged)	742.49 ± 222.32 (wk12)	469.03 ± 72.59 (wk12)	80.77 ± 15.45 (wk12)
	GAN	390.9 ± 160.54 (wk32)	296.6 ± 120.76 (wk32)	337.43 ± 70.51 (wk32)
	GAN + Low dose CCl4	156.51 ± 124.58 (wk24)	162.9 ± 175.7 (wk24)	113.04 ± 19.56 (wk24)
	GAN (ob/ob)	987.98 ± 219.26 (wk31)	823.3 ± 177.07 (wk31)	538.65 ± 85.63 (wk31)
	FDSW	415.24 ± 210.18 (wk23)	266.71 ± 94.9 (wk23)	311.59 ± 104.31 (wk23)
	FDSW + Low dose CCl4	363.06 ± 125.28 (wk24)	302.55 ± 92.21 (wk24)	164.42 ± 50.91 (wk24)
	Chow (ob/ob)	727.33 ± 309.98 (wk31)	709.77 ± 446.89 (wk31)	293.43 ± 43.33 (wk31)
TN	*CDAHFD*	372.38 ± 71.17 (wk16)		
	GAN	491.17 ± 186.16 (wk32)	317.23 ± 87.68 (wk32)	381.7 ± 54.41 (wk32)
	FDSW	308.14 ± 124.65 (wk23)	191.75 ± 69.62 (wk23)	309.25 ± 54.64 (wk23)

Proprietary

Supplementary Table 2. Differential gene expression analysis between GAN with normal diet under each temperature separately

89 pathways exclusive in Ambient:

- Natural killer cell mediated cytotoxicity
- Rheumatoid arthritis
- Toll-like receptor signaling pathway
- Fc epsilon RI signaling pathway
- Viral protein interaction with cytokine and cytokine receptor
- Yersinia infection
- Th17 cell differentiation
- Inflammatory bowel disease
- NOD-like receptor signaling pathway
- TNF signaling pathway
- Triacylglycerol Degradation
- IL-10 Signaling
- Factors Promoting Cardiogenesis in Vertebrates
- Acyl-CoA Hydrolysis
- MIF Regulation of Innate Immunity
- Creatine-phosphate Biosynthesis
- Semaphorin Signaling in Neurons
- Salvage Pathways of Pyrimidine Ribonucleotides
- iNOS Signaling
- Neuropathic Pain Signaling In Dorsal Horn Neurons
- Nur77 Signaling in T Lymphocytes
- Choline metabolism in cancer
- Pertussis
- Pentose and glucuronate interconversions
- Pyridoxal 5'-phosphate Salvage Pathway
- Adrenomedullin signaling pathway
- Serotonin Degradation
- Cardiac Hypertrophy Signaling (Enhanced)
- Melatonin Signaling
- Melatonin Degradation II
- Neurotrophin/TRK Signaling
- Retinoate Biosynthesis I
- ErbB Signaling
- Thyroid Cancer Signaling
- CD40 Signaling
- Thyroid Hormone Metabolism II (via Conjugation and/or Degradation)
- Lymphotoxin Œ≤ Receptor Signaling
- Role of IL-17A in Arthritis
- Aldosterone Signaling in Epithelial Cells
- Mechanisms of Viral Exit from Host Cells
- Alanine, aspartate and glutamate metabolism
- Arginine biosynthesis
- Coronavirus disease - COVID-19
- Phenylalanine, tyrosine and tryptophan biosynthesis
- Fatty acid degradation
- Primary bile acid biosynthesis
- Glycolysis/ Gluconeogenesis
- Urea Cycle
- Superpathway of Citrulline Metabolism
- IL-17A Signaling in Gastric Cells
- Phenylalanine Degradation IV (Mammalian, via Side Chain)
- Citrulline-Nitric Oxide Cycle
- JAK-STAT signaling pathway
- Fatty acid biosynthesis
- ERK/MAPK Signaling
- Antiproliferative Role of Somatostatin Receptor 2
- ErbB4 Signaling
- Glutamine Degradation I
- TNFR2 Signaling
- VEGF Family Ligand-Receptor Interactions
- IL-2 Signaling
- G-Protein Coupled Receptor Signaling
- Regulation of the Epithelial-Mesenchymal Transition Pathway
- FcŒ≥RIIB Signaling in B Lymphocytes
- Ovarian Cancer Signaling
- Apelin Endothelial Signaling Pathway
- Cardiac Hypertrophy Signaling
- IL-17 Signaling
- FLT3 Signaling in Hematopoietic Progenitor Cells
- GNRH Signaling
- Citrulline Biosynthesis
- Agrin Interactions at Neuromuscular Junction
- Mitochondrial L-carnitine Shuttle Pathway
- Antioxidant Action of Vitamin C
- Relaxin Signaling
- HMGB1 Signaling
- TNFR1 Signaling
- UVC-Induced MAPK Signaling
- April Mediated Signaling
- SAPK/JNK Signaling
- CTLA4 Signaling in Cytotoxic T Lymphocytes
- RANK Signaling in Osteoclasts
- Role of PI3K/AKT Signaling in the Pathogenesis of Influenza
- Mouse Embryonic Stem Cell Pluripotency
- Regulation of IL-2 Expression in Activated and Anergic T Lymphocytes
- B Cell Activating Factor Signaling
- Role of Osteoblasts, Osteoclasts and Chondrocytes in Rheumatoid Arthritis
- Oncostatin M Signaling
- Cell Cycle Control of Chromosomal Replication

29 pathways exclusive in Thermoneutrality:

- Fat digestion and absorption
- Autoimmune thyroid disease
- Fatty acid elongation
- Neomycin, kanamycin and gentamicin biosynthesis
- Type II diabetes mellitus
- ABC transporters
- Galactose metabolism
- Phagosome Maturation
- Glutathione Redox Reactions I
- Glycolysis I
- Circadian rhythm
- Kinetochore Metaphase Signaling Pathway
- Bladder Cancer Signaling
- Mitotic Roles of Polo-Like Kinase
- p53 Signaling
- Circadian Rhythm Signaling
- Vascular smooth muscle contraction
- Hippo signaling pathway - multiple species
- Inflammasome pathway
- Human Embryonic Stem Cell Pluripotency
- Protein Kinase A Signaling
- Tight junction
- Starch and sucrose metabolism
- Hepatocellular carcinoma
- Docosahexaenoic Acid (DHA) Signaling
- Glycogen Biosynthesis II (from UDP-D-Glucose)
- RhoA Signaling
- Chronic Myeloid Leukemia Signaling
- Sumoylation Pathway

Supplementary Table 3. Categorization of steatosis features derived from SHG/TPEF images

Categories	Measurements	Regions
Steatosis	Percentage of steatosis area	Overall, CV, PS, PT
Macrosteatosis	Percentage of macrosteatosis area	
Fat vacuoles (FV)	Number of FV<br>Percentage of hepatocytes with FV<br>Average distance between one FV and the closest FV<br>Variance distance between one FV and the closest FV<br>Average number of FVs within 100 µm of a FV<br>Variance number of FVs within 100 µm of a FV	
Macro FV	Number of macro FV	

Supplementary Table 4. Categorization of fibrosis features derived from SHG/TPEF images

Categories	Measurements	Regions
Total collagen<br>Aggregated collagen<br>Distributed collagen	Percentage of total collagen<br>Percentage of aggregated collagen<br>Percentage of distributed collagen	Overall, CV, PS, PT
Total strings, aggregated strings, distributed strings	Number of strings<br>Number of short strings<br>Number of long strings<br>Number of thin strings<br>Number of thick strings<br>String area<br>String length<br>String width	Overall, CV, PS, PT
Total strings	String eccentricity<br>String solidity<br>String perimeter<br>String orientation	Overall
	Number of intersections	Overall, CV, PS, PT

Supplementary Table 5. The relationship between the stringency of the parameter set for the inflammatory cell detection model, number of cells detected as well as false positive rate (FPR). The model was run on 60 test patches (526 pixel x 526 pixel) with pathologist ground truth, while varying StarDist & SAM probability thresholds; NMS set at 0.1.

AI parameter threshold set		Average number of inflammatory cells detected per patch	Increase in the number of inflammatory cells detected*	FPR	Increase in FPR*
StarDist	SAM				
0.5	0.3	29.3	4.1	13.4%	7.2
0.7	0.3	24.0	3.3	10.7%	5.8
0.8	0.3	13.7	1.9	5.5%	3.0
0.5	0.5	14.9	2.1	4.6%	2.5
0.7	0.5	12.3	1.7	3.6%	1.9
0.8	0.5	7.2	1	1.9%	1

**The increase is calculated with respect to the value for the most stringent parameter set. Grey highlight indicates the current model parameter set.*

Figures

Supplementary Fig. 1. Multi-omics analysis of GAN models.

Supplementary Fig. 1 A. Comparison of NASH progression between GAN diet and normal chow (NC)–fed mice (C57BL/6J) using differentially expressed (DE) genes identified by bulk RNA sequencing. Shown are pathway-level summaries of DE genes that implicate cholesterol biosynthesis, immune response, fibrosis, and tumor-related processes, indicating convergent functional changes associated with disease progression. Sample sizes: GAN (TA or TN) wk12, wk16, wk23, n = 8 per group; GAN TA wk32, n = 7; GAN TN wk32, n = 6, NC (TA or TN) wk23, n=8 per group. Age : 7weeks upon start of GAN diet (TA/TN).

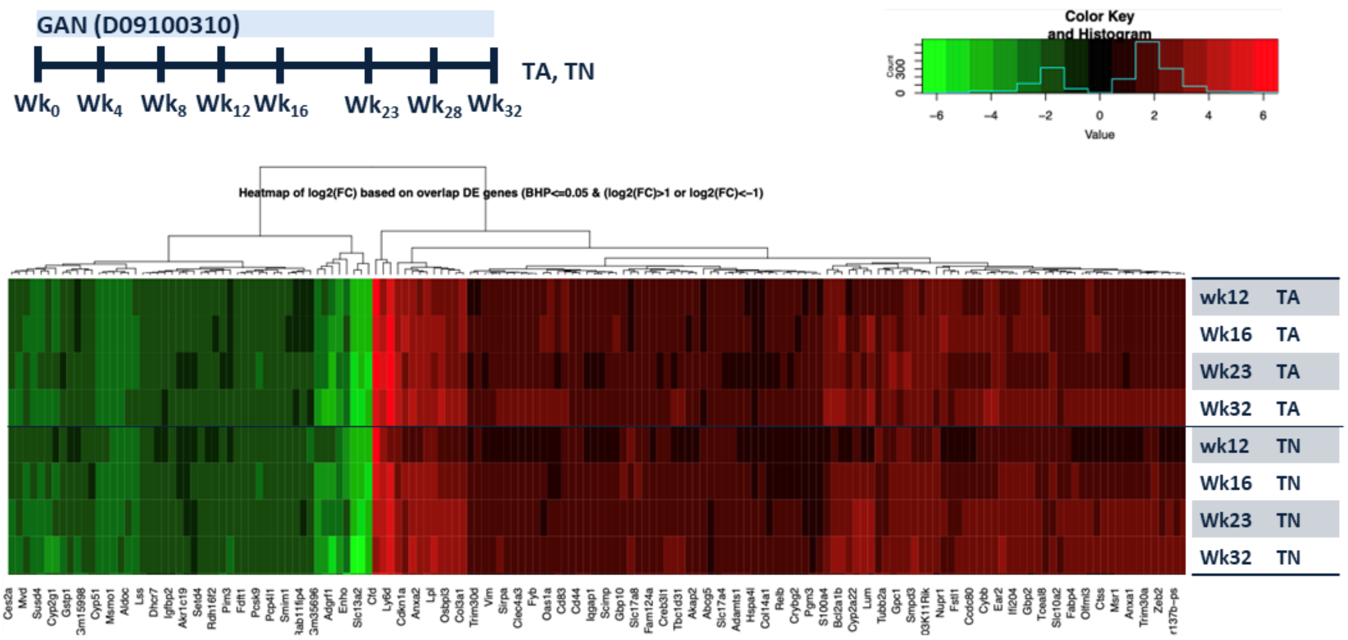

Supplementary Fig. 1 B. Pathway Enrichment analysis evaluating the effect of housing temperature in GAN fed mice (C57BL/6J). TN housing upregulates expression of genes and pathways associated with MASH including hepatic fibrosis/hepatic stellate cell activation, protein digestion and absorption, leukocyte migration involved in inflammatory response, TGF-β signaling pathway, regulation of macrophage migration, neutrophil migration, collagen binding. TN housing downregulates genes associated with kidney development and rhythmic process. Sample sizes: GAN (TA or TN) wk12, wk16, wk23, n = 8 per group; GAN TA wk32, n = 7; GAN TN wk32, n = 6, NC (TA or TN) wk23, n=8 per group. Age : 7weeks upon start of GAN diet (TA/TN).

UP-REGULATED

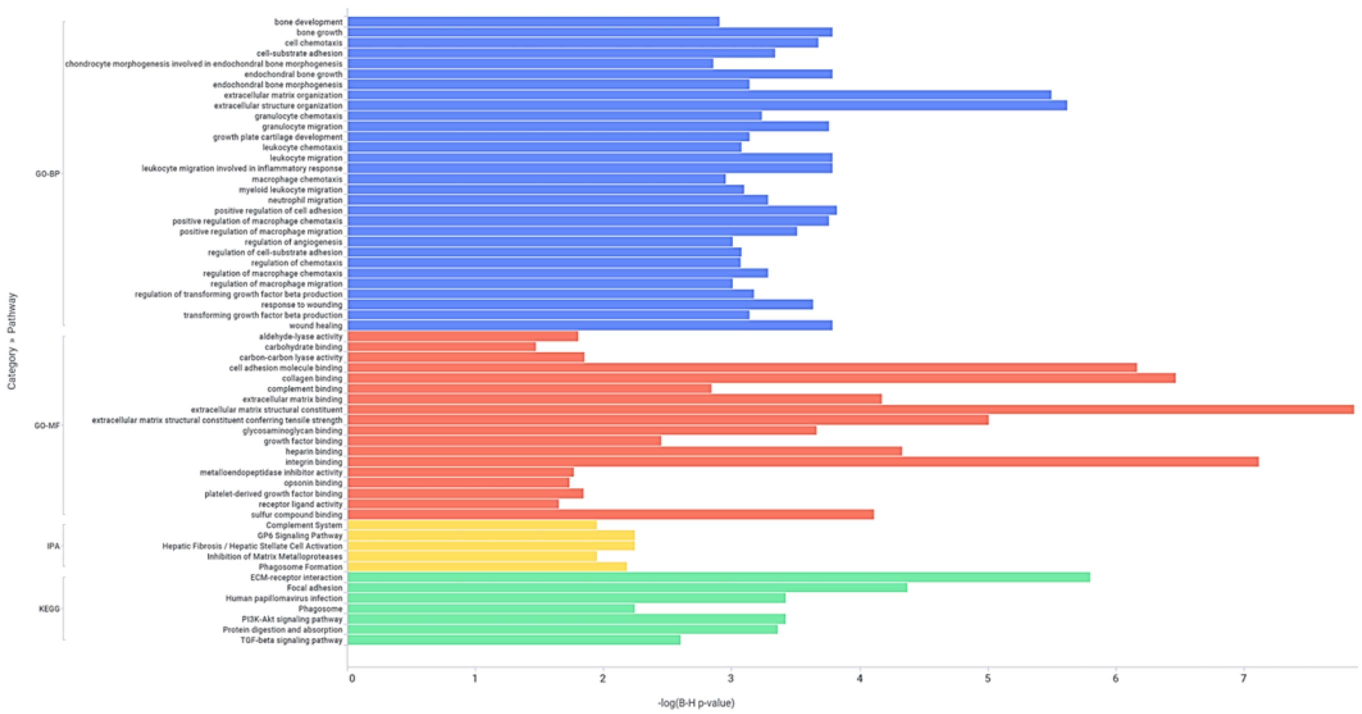

DOWN-REGULATED

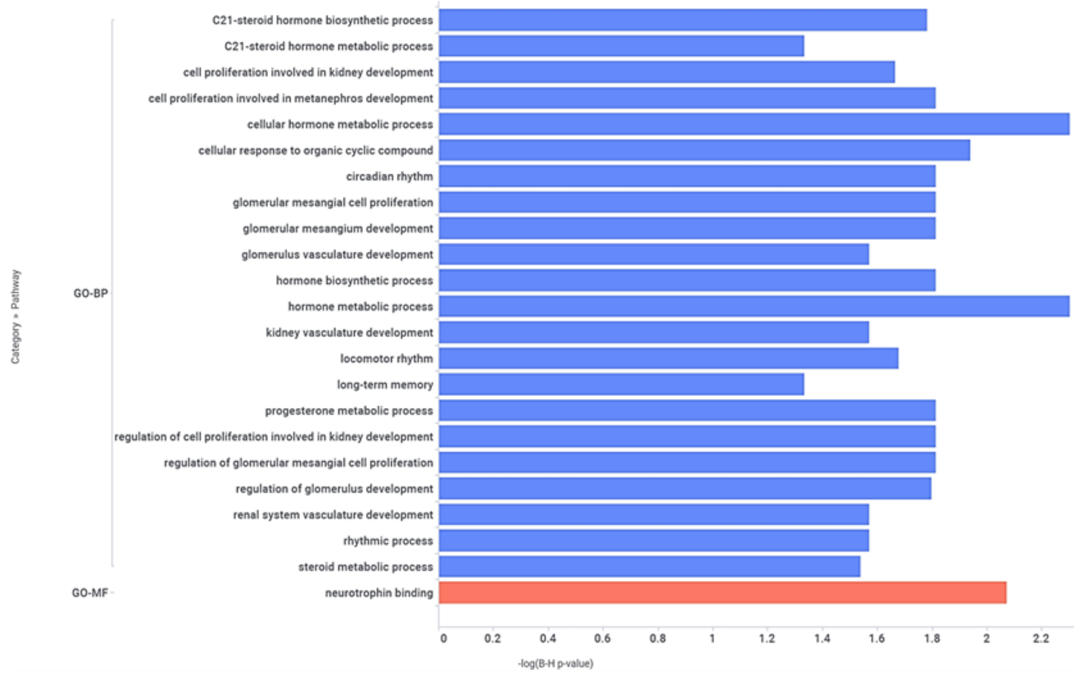

Supplementary Fig. 1 C. Enrichment analysis of lipidomic data in GAN and normal chow diet fed mice (C57BL/6J). In Liver, GAN activates cholesteryl ester (CE) and triacylglycerol (TAG) level while decreases phosphatidylcholine (PC) and phosphatidylethanolamine (PE) level. In Plasma, GAN increases CE and PC level yet decreases TAG level. Sample sizes: GAN (TA or TN) wk23, n = 5 per group. Age : 7weeks upon start of GAN diet (TA/TN).

C.

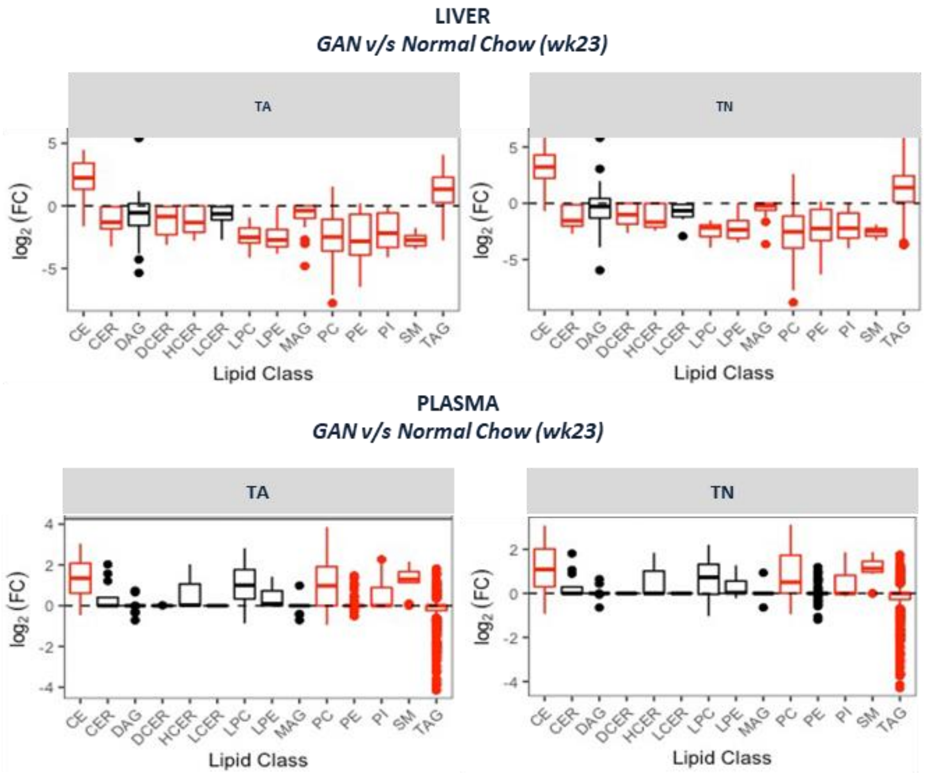

Supplementary Fig. 1 D. TAG Fatty Acid Chain Saturation analysis in GAN and normal chow diet fed mice (C57BL/6J) at TA and TN. TN up-regulates TAGs with short chain and high saturation level. High fat diet up-regulate TAGs1 with short chain and high saturation level while down-regulate TAGs with long chain and low saturation level. Sample sizes: GAN (TA or TN) wk23, n = 5 per group. Age : 7weeks upon start of GAN diet (TA/TN).

D.

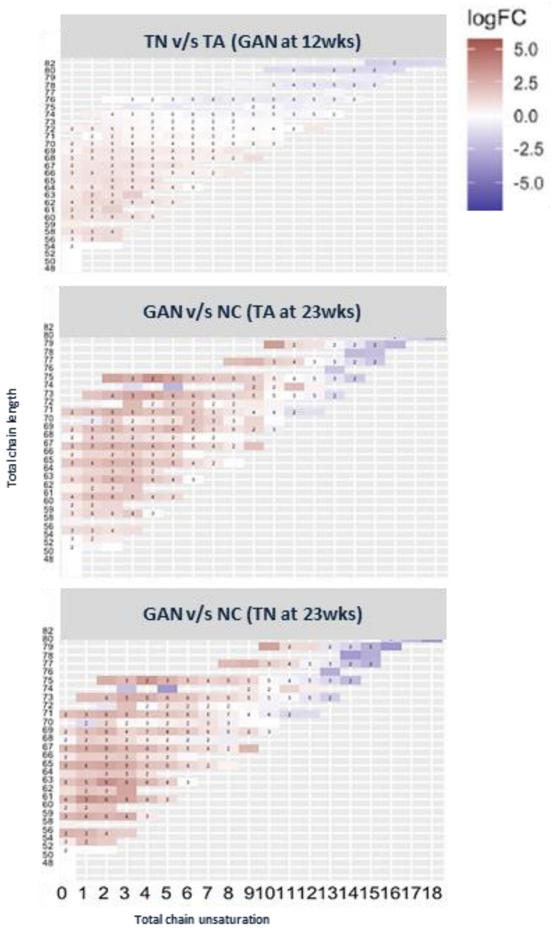

Proprietary

Supplementary Fig. 1 E. Heatmap of metabolite abundance by subclass in GAN and normal chow diet fed mice (C57BL/6J). GAN diet upregulates Sphingolipid synthesis in liver and plasma. In Liver, GAN diet down-regulates metabolites associated with vitamin A, Alanine, Aspartate, Glutamate, Histidine, Benzoate and drug metabolism. In Plasma, GAN down-regulates metabolites associated with Tyrosine, Alanine, Aspartate, Lysine, PG, PE, and drug metabolism. SD = Steinberg/GAN diet. Sample sizes: Normal Chow (NC), GAN TA or TN, n = 5 per group. Age : 7weeks upon start of GAN diet (TA/TN).

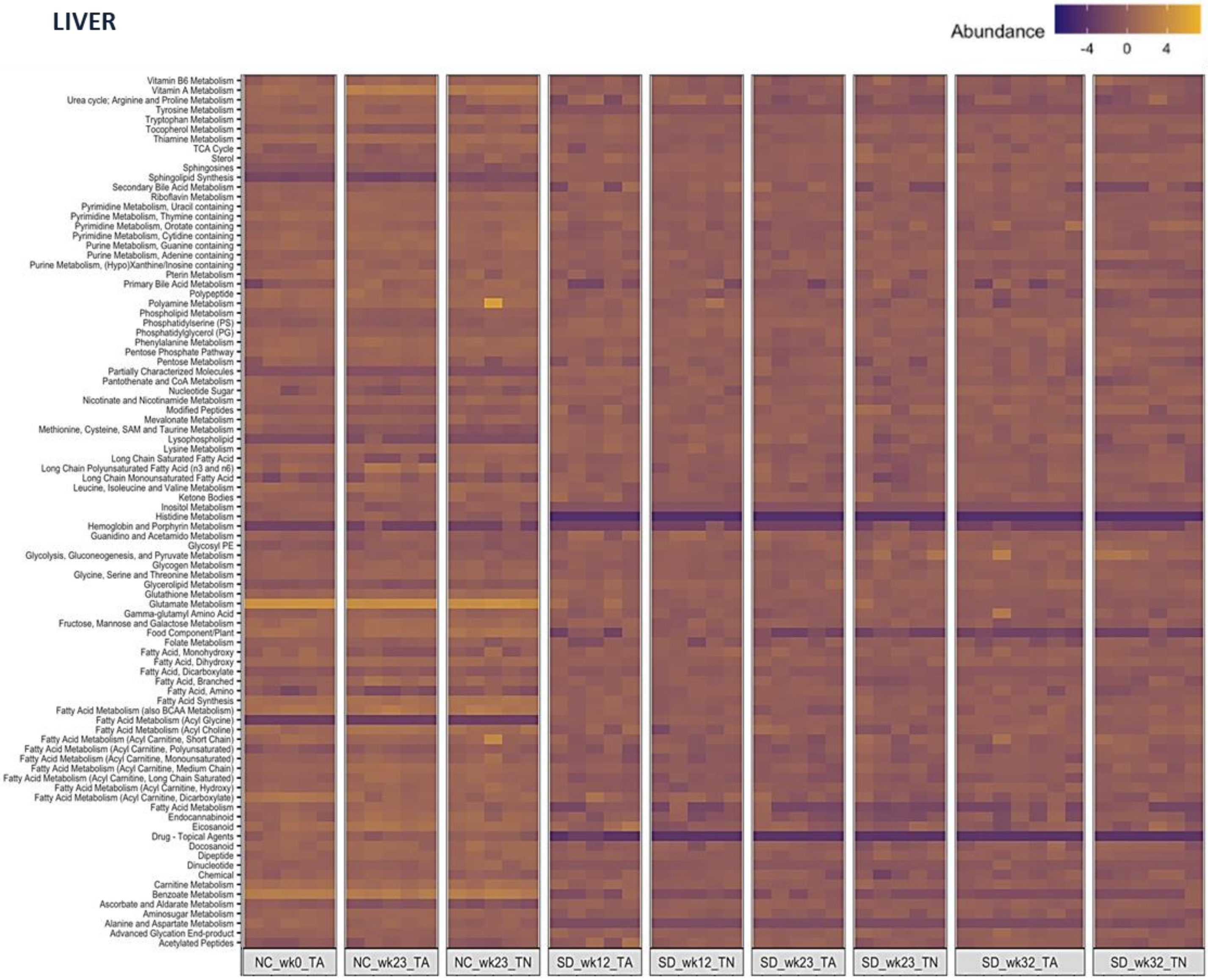

PLASMA

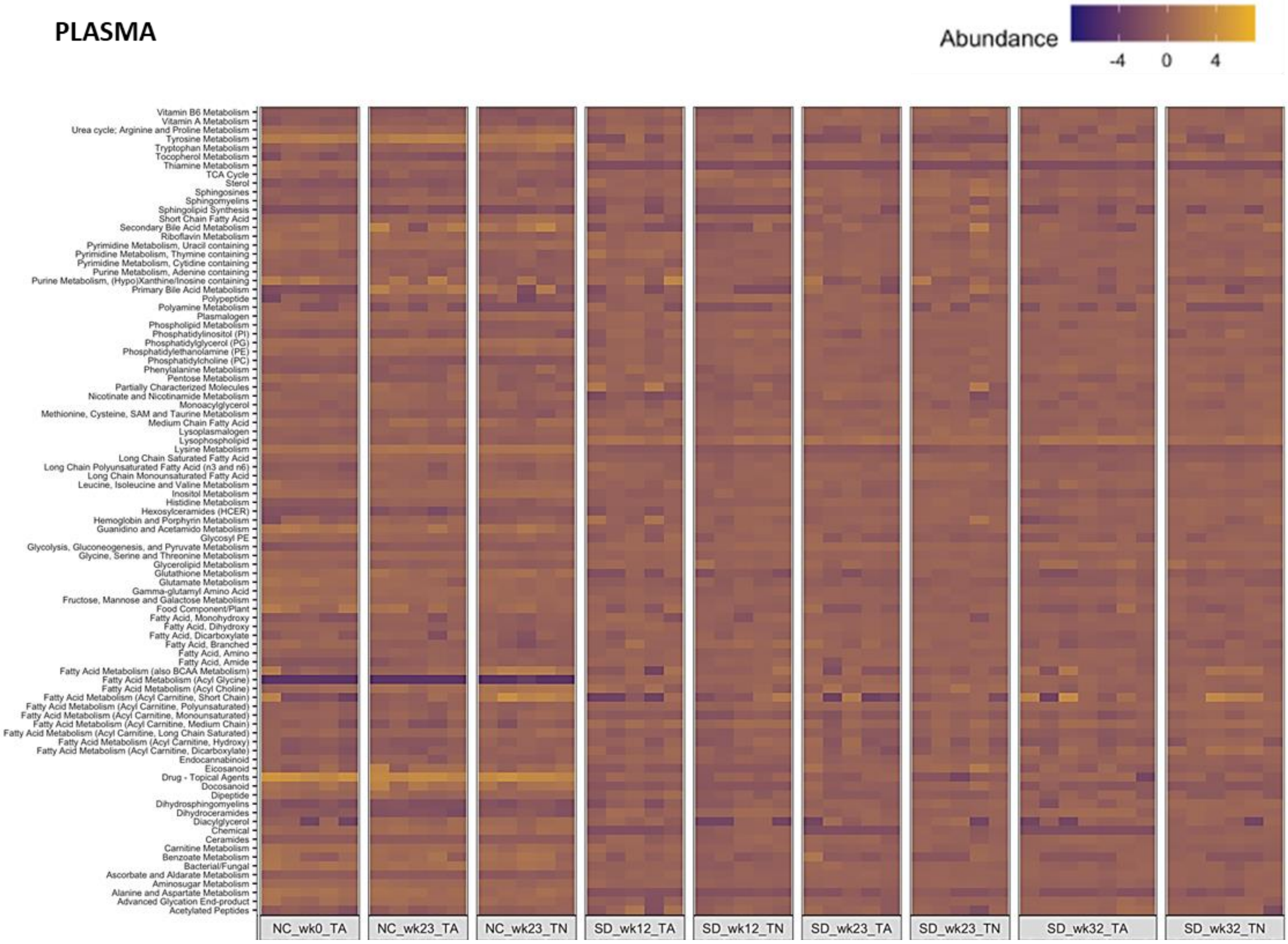
Abundance
-4
0
4
Vitamin B6 Metabolism
Vitamin A Metabolism
Urea cycle; Arginine and Proline Metabolism
Tyrosine Metabolism
Tryptophan Metabolism
Tocopherol Metabolism
Thiamine Metabolism
TCA Cycle
Sterol
Sphingosines
Sphingomyelins
Sphingolipid Synthesis
Short Chain Fatty Acid
Secondary Bile Acid Metabolism
Riboflavin Metabolism
Pyrimidine Metabolism, Uracil containing
Pyrimidine Metabolism, Thymine containing
Pyrimidine Metabolism, Cytidine containing
Purine Metabolism, Adenine containing
Purine Metabolism, (Hypo)Xanthine/Inosine containing
Primary Bile Acid Metabolism
Polypeptide
Polyamine Metabolism
Plasmalogen
Phospholipid Metabolism
Phosphatidylinositol (PI)
Phosphatidylglycerol (PG)
Phosphatidylethanolamine (PE)
Phosphatidylcholine (PC)
Phenylalanine Metabolism
Pentose Metabolism
Partially Characterized Molecules
Nicotinate and Nicotinamide Metabolism
Monoacylglycerol
Methionine, Cysteine, SAM and Taurine Metabolism
Medium Chain Fatty Acid
Lysoplasmalogen
Lysophospholipid
Lysine Metabolism
Long Chain Saturated Fatty Acid
Long Chain Polyunsaturated Fatty Acid (n3 and n6)
Long Chain Monounsaturated Fatty Acid
Leucine, Isoleucine and Valine Metabolism
Inositol Metabolism
Histidine Metabolism
Hexosylceramides (HCER)
Hemoglobin and Porphyrin Metabolism
Guanidino and Acetamido Metabolism
Glycosyl PE
Glycolysis, Gluconeogenesis, and Pyruvate Metabolism
Glycine, Serine and Threonine Metabolism
Glycerolipid Metabolism
Glutathione Metabolism
Glutamate Metabolism
Gamma-glutamyl Amino Acid
Fructose, Mannose and Galactose Metabolism
Food Component/Plant
Fatty Acid, Monohydroxy
Fatty Acid, Dihydroxy
Fatty Acid, Dicarboxylate
Fatty Acid, Branched
Fatty Acid, Amino
Fatty Acid, Amide
Fatty Acid Metabolism (also BCAA Metabolism)
Fatty Acid Metabolism (Acyl Glycine)
Fatty Acid Metabolism (Acyl Choline)
Fatty Acid Metabolism (Acyl Carnitine, Short Chain)
Fatty Acid Metabolism (Acyl Carnitine, Polyunsaturated)
Fatty Acid Metabolism (Acyl Carnitine, Monounsaturated)
Fatty Acid Metabolism (Acyl Carnitine, Medium Chain)
Fatty Acid Metabolism (Acyl Carnitine, Long Chain Saturated)
Fatty Acid Metabolism (Acyl Carnitine, Hydroxy)
Fatty Acid Metabolism (Acyl Carnitine, Dicarboxylate)
Endocannabinoid
Eicosanoid
Drug - Topical Agents
Docosanoid
Dipeptide
Dihydrosphingomyelins
Dihydroceramides
Diacylglycerol
Chemical
Ceramides
Carnitine Metabolism
Benzoate Metabolism
Bacterial/Fungal
Ascorbate and Aldarate Metabolism
Aminosugar Metabolism
Alanine and Aspartate Metabolism
Advanced Glycation End-product
Acetylated Peptides
NC_wk0_TA
NC_wk23_TA
NC_wk23_TN
SD_wk12_TA
SD_wk12_TN
SD_wk23_TA
SD_wk23_TN
SD_wk32_TA
SD_wk32_TN

Proprietary

Supplementary Fig. 1 F. Summary of metabolomic changes by class in GAN and normal chow diet fed mice (C57BL/6J). In the liver and plasma, GAN diet affects lipid and animo acid level the most as the disease progresses. Sample sizes: Normal Chow (NC), GAN TA or TN, n = 5

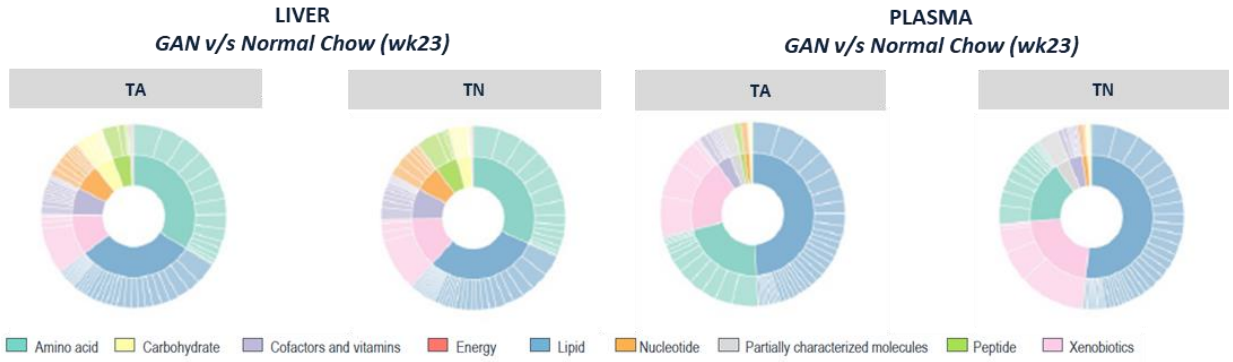

per group. Age : 7weeks upon start of GAN diet (TA/TN).

TOP DIFFERENTIALLY EXPRESSED METABOLITES UPREGULATED (LIVER)

Lipids	Amino Acids	Nucleotides	Cofactors and Vitamins	Xenobiotics
cis-3,4-methyleneheptanoylglycine, fatty acid metabolism<br>1-oleoyl-GPG(18:1)*, Lysophospholipid<br>Trimethylamine N-oxide, Phospholipid Metabolism<br>1-deoxysphinganine (m18:0), Sphingolipid	S-methylcysteine, Methionine, Cysteine, SAM and Taurine Metabolism<br>Putrescine, Polyamine Metabolism<br>Glutamine, Glutamate Metabolism<br>p-cresol glucuronide*, Tyrosine Metabolism	3-ureidopropionate | Pyrimidine Metabolism<br>UDP-glucose |Nucleotide Sugar	Heme | Hemoglobin and Porphyrin Metabolism<br>Ascorbate (Vitamin C) | Ascorbate and Aldarate Metabolism	4-methylbenzenesulfonate p-cresol sulfate | Benzoate Metabolism

TOP DIFFERENTIALLY EXPRESSED METABOLITES DOWNREGULATED (LIVER)

Lipids	Amino Acids	Cofactors and Vitamins	Xenobiotics
Heneicosapentaenoate (21:5n3), Long Chain PUFA<br>(12 or 13)-methylmyristate (a15:0 or i15:0), Branched Fatty Acid	N-acetylhistamine | Histidine Metabolism<br>N-methyl-GABA, Glutamate Metabolism<br>Spermine | Polyamine Metabolism	Trigonelline (N'-methylnicotinate) | Nicotinate and Nicotinamide Metabolism	Equol sulfate<br>Histidine betaine (hercynine)<br>Perfluorooctanesulfonate<br>2,6-dihydroxybenzoic acid<br>Ergothioneine<br>3-(3-hydroxyphenyl) propionate sulfate|Benzoate Metabolism<br>Catechol sulfate|Benzoate Metabolism<br>2,6-dihydroxybenzoic acid<br>Stachydrine<br>Enterolactone sulphate

TOP DIFFERENTIALLY EXPRESSED METABOLITES UPREGULATED (PLASMA)

Lipids	Amino Acids	Nucleotides	Xenobiotics
Cis-3,4-methyleneheptanoylglycine|Fatty Acid Metabolism<br>Taurocholate|Primary Bile Acid Metabolism<br>Tauro-beta-muricholate|Primary Bile Acid Metabolism<br>Tauroursodeoxycholate|Secondary Bile Acid Metabolism<br>Glycocholate|Primary Bile Acid Metabolism<br>Trans-3,4-methyleneheptanoylglycine|Fatty Acid Metabolism<br>Docosatrienoate (22:3n6)*|Long Chain Polyunsaturated Fatty Acid<br>Ceramide (d16:1/24:1, d18:1/22:1)*<br>N-palmitoyl-sphingosine (d18:1/16:0)|Ceramides	p-cresol glucuronide*|Tyrosine Metabolism<br>S-methylcysteine sulfoxide|Methionine, Cysteine, SAM and Taurine Metabolism	3-ureidoisobutyrate|Pyrimidine Metabolism	p-cresol sulfate|Benzoate Metabolism

TOP DIFFERENTIALLY EXPRESSED METABOLITES DOWNREGULATED (PLASMA)

Lipids	Amino Acids	Xenobiotics
Heneicosapentaenoate (21:5n3)|Long Chain Polyunsaturated	Indolepropionate|Tryptophan Metabolism	Equol sulfate<br>Homostachydrine<br>Ergothioneine<br>2,6-dihydroxybenzoic acid<br>2-aminophenol sulfate<br>Ectoine<br>2,8-quinolinediol sulfate<br>3-hydroxycinnamate sulfate

Supplementary Fig. 2. Representative H&E images for progression in GAN (C57BL/6J). H&E images show lipid-droplet formation in CV and PT for GAN at (A) TA and (B) TN conditions. At TN, microsteatosis start to be well-defined at 12 weeks, compared to TA, which was only prominent at 23 weeks. Similar observation can be appreciated for macrosteatosis. Following the zone-specific metabolic gradient, microsteatosis development is very clearly seen surrounding CV, while macrosteatosis is present surrounding PT. White bar represents 100 µm. Age : 7weeks upon start of GAN diet (TA/TN).

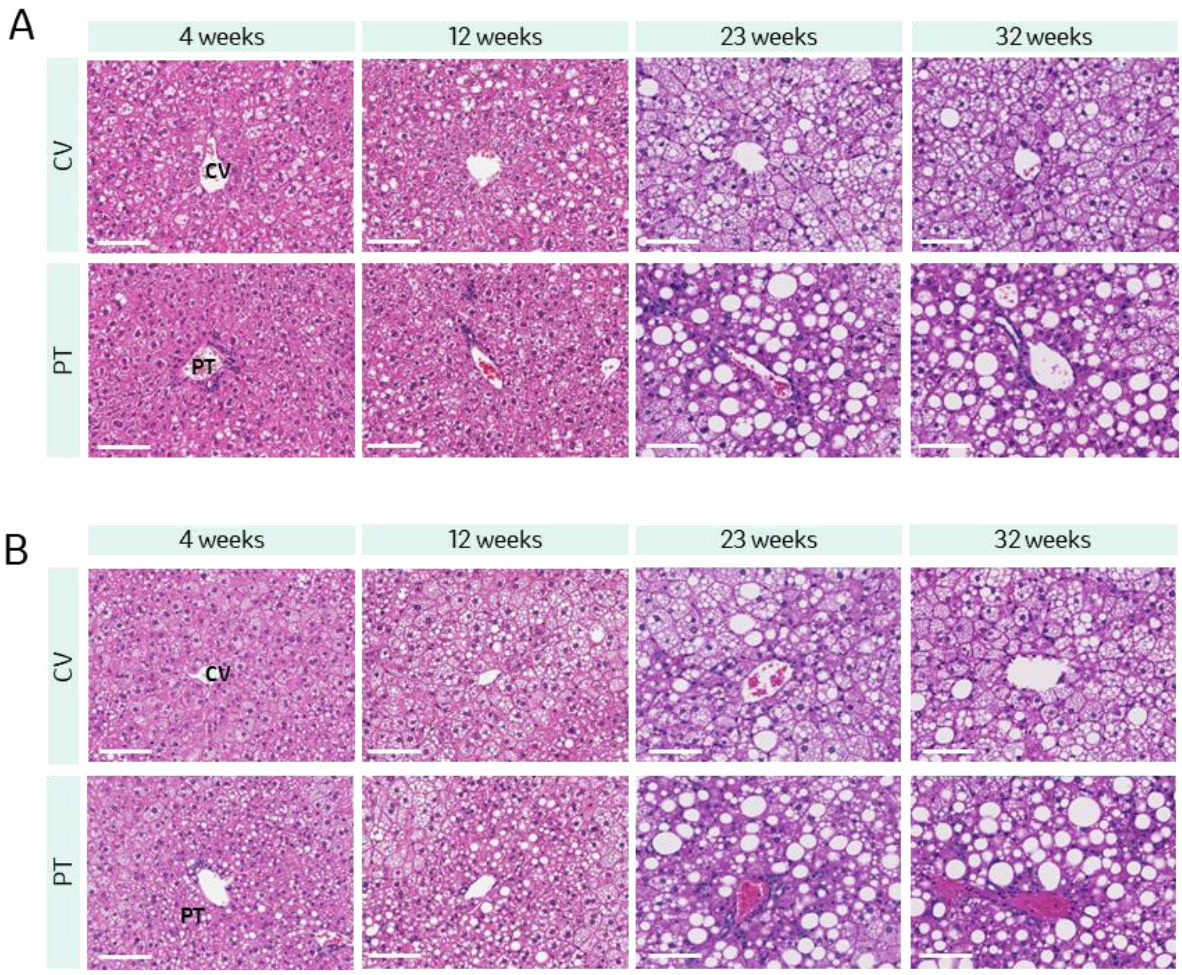

Supplementary Fig. 3. Representative H&E images for progression in CDAHFD model (C57BL/6N) at TN. (A) H&E images show lipid-droplet formation in CV and PT. From 4 weeks, lipid-droplet profile is saturated with macrosteatosis. Zone differences that could be appreciated in the GAN model (Supplementary Fig.5) cannot be appreciated here, as macrosteatosis are present surrounding CV as well as PT. The trend for reduction in macrosteatosis at a later timepoint can be appreciated as well, which is seen with the increasing presence of inflammation. (B) A representative image for inflammation in CDAHFD model (data from week 8). The presence of foamy pigment laden cells (pigment granuloma, red arrows; refer to main document Fig. 7), lipogranuloma (blue arrows), inflammatory foci (yellow arrows) can also be seen. White bar represents 100 µm. Age : 7weeks upon start of CDAHFD.

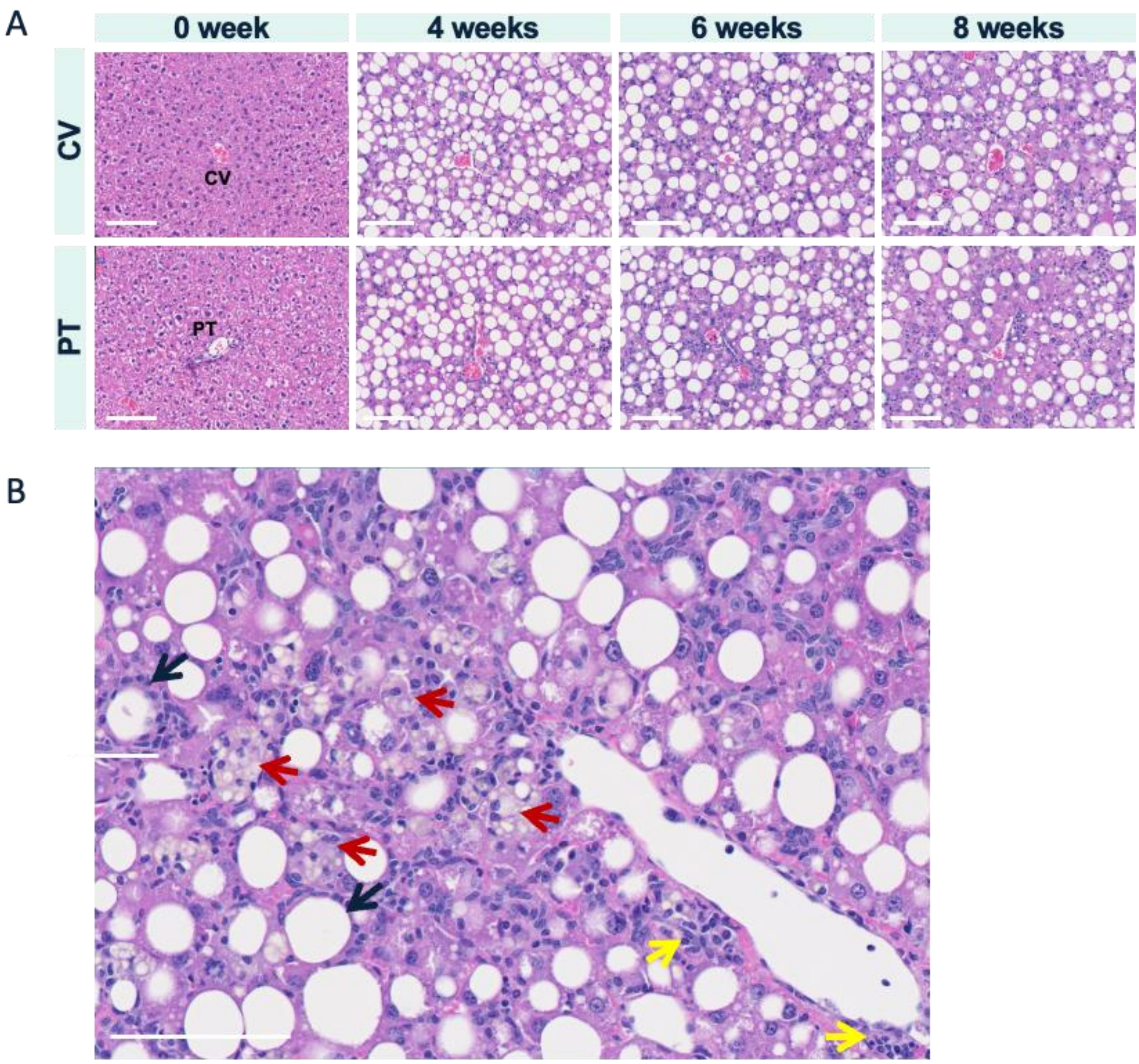

Supplementary Fig. 4. Validation of AI model for inflammatory cell detection. (A-B) Correlation between AI-derived inflammatory cell density (A) or inflammatory cell clusters (B) with MASH-CRN lobular inflammation score. High correlation between inflammatory cell density and pathologist score was shown. (C) Correlation between AI-derived inflammatory cell density on H&E sections and the density of all T-cells (CD3+, CD4+, or CD8+) and macrophages (F4/80+, CD11b+, CD68+, or CD44+) as detected on multiplexed-IF. The T-cell panel and macrophage panel were performed on a different section; H&E was performed on the same section post-mIF. The H&E-based inflammatory cell density (x-axis) was the average of the inflammatory cells detected on the two corresponding H&E sections. Data from CDAHFD model at TA (young cohort): (A-B) N=108 (I0: N=15, I1: N=18, I2: N=28, I3: N=9), (C) N=79. Statistics: (A-B) Spearman's correlation, (C) Pearson's correlation. Boxplot indicates median ± first quartile or third quartile. Age : 8weeks upon start of CDAHFD.

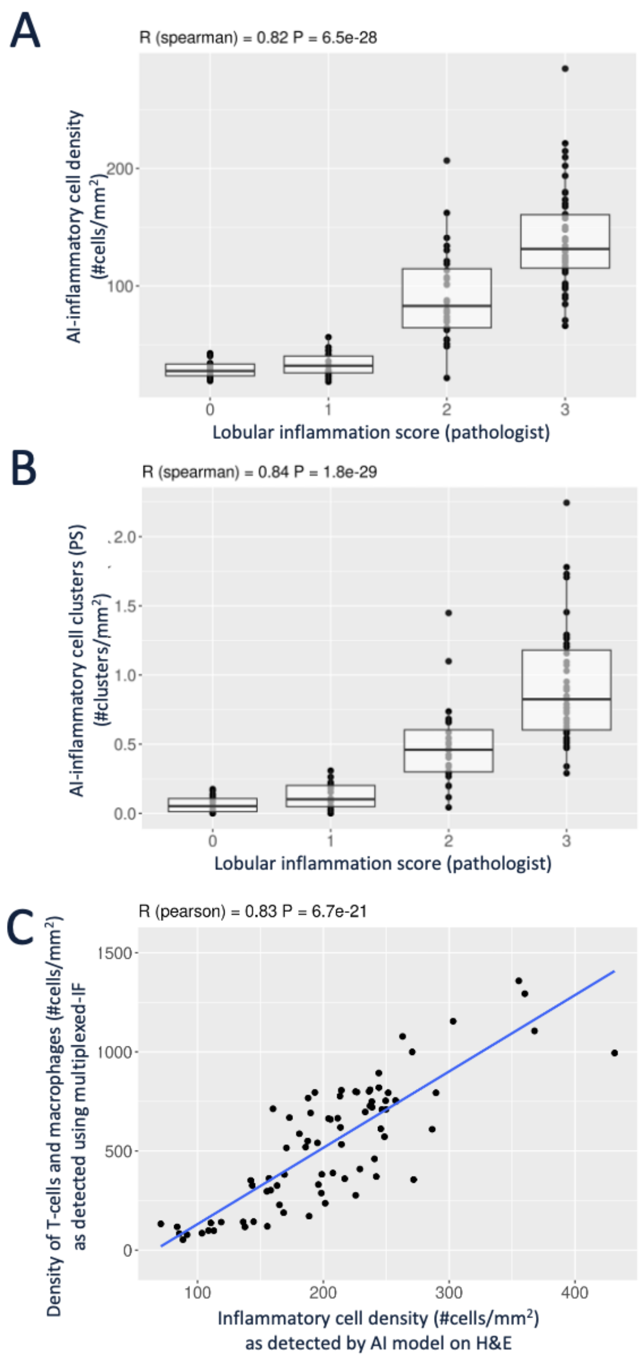

Supplementary Fig. 5. NASH-CRN inflammation scores as evaluated by pathologists for different animal models (C57BL/6J). Representative NASH‑CRN inflammation scores, as evaluated by pathologists, for mice maintained on either GAN diet or chow for 4, 12, 23 or 32 weeks under two housing conditions: (A) TA and (B) TN. Bars show group means ± SD. Sample sizes: (A) chow, n = 8; GAN, n = 6–8 per timepoint; (B) chow, n = 8; GAN, n = 7–8 per timepoint. Statistical analysis: one‑way ANOVA with Bonferroni's multiple comparisons test. Reported pairwise comparisons for GAN under TN housing: week 4 versus week 12, p = 0.0176; week 12 versus week 23, p = 0.0176; week 12 versus week 32, p = 0.0062. Age : 7weeks upon start of GAN diet (TA/TN).

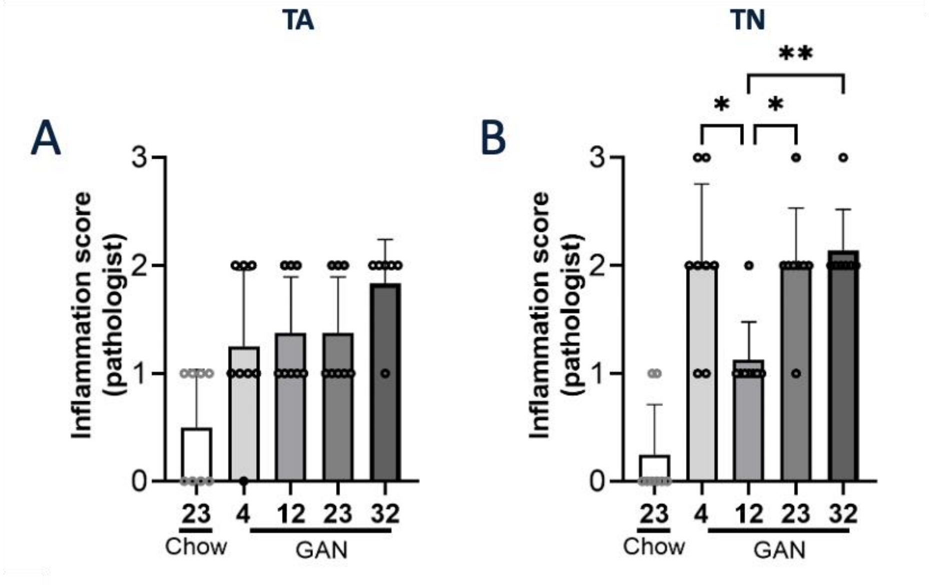

Supplementary Fig. 6. Pigmented granuloma. (A) Correlation between AI model of pigmented granuloma and pathologist score. The area of pigment granuloma detected using AI model is strongly correlated with the pathologist's scores of foamy pigment laden cells (Spearman's R = 0.89, P<0.001). (B) Correlation between AI-derived pigment granuloma area and inflammation, (C) Correlation between AI-derived pigment granuloma area and fibrosis. Dataset: (A) CDAHFD at TN, N=113 (pathologist score 0: N=42, score 1: N=19, score 2: N=28, score 3: N=24), (B-C) CDAHFD-TA young N=40. Statistics: (A) Spearman's correlation, (B-C) Pearson's correlation, performed for data points with pigmented granuloma area larger than 0.05% tissue (generally data point after week 4). Boxplot indicates median ± first quartile or third quartile. Age : 7weeks upon start of CDAHFD (TN), 8 weeks upon start of CDAHFD (TA).

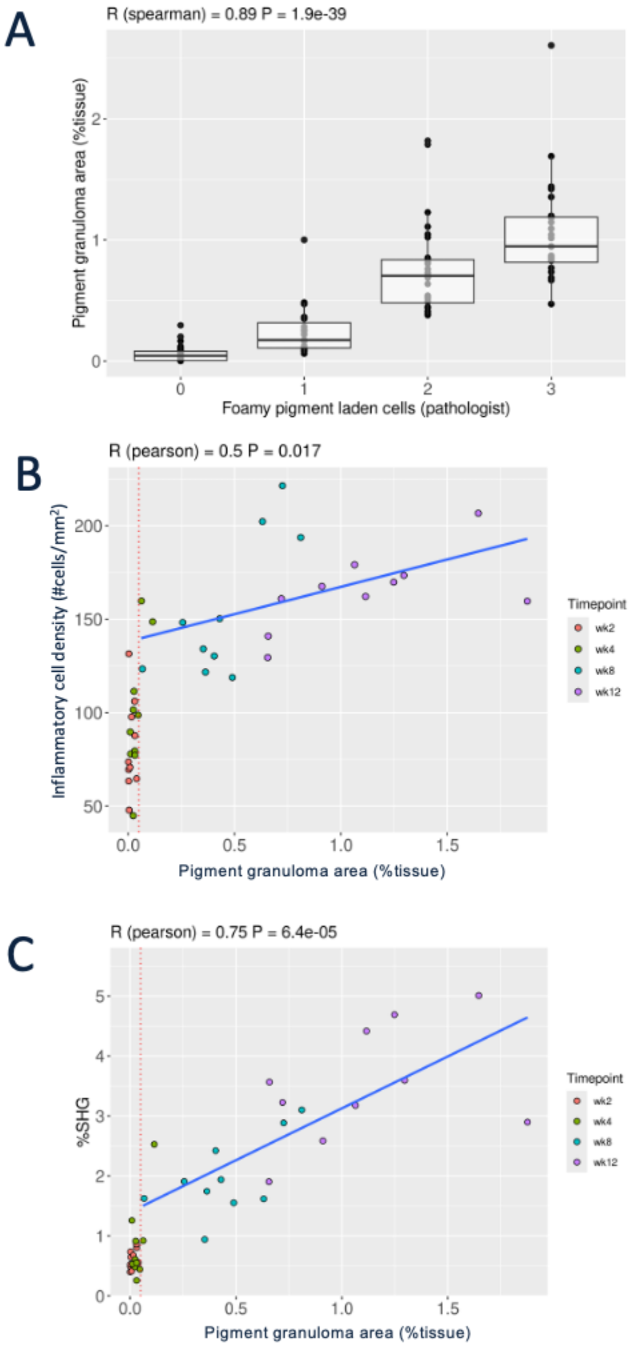

Supplementary Fig. 7. The effects of GLP1 agonism (Semaglutide) on GAN diet induced steatosis, inflammation and fibrosis in male mice (C57BL/6J). Representative histology and quantitative assessment (Blood chemistry, mRNA expression profiling) showing that pharmacological GLP1 agonism reduces hepatic steatosis, inflammation, and fibrosis in mice with diet-induced MASH. Groups: Veh, Sema. Sample sizes: n = 8 per treatment. Statistical testing: Mann–Whitney Unpaired test : ASMA, p = 0.0011; Galectin-3 p=0.0354, Col1a1 p=0.0207, ALT p=0.0002, AST p=0.0011, total cholesterol (Chol) p=0.0006; mRNA expression (*Col1a1* p=0.0140, *Col3a1* p=0.0022, *Timp1* p=0.0022, *Adgre1* p=0.0011, *Ccl2* p=0.0008, *Ccr2* p=0.0006, *Ccr5* p=0.0093). Age : 7weeks upon start of GAN diet.

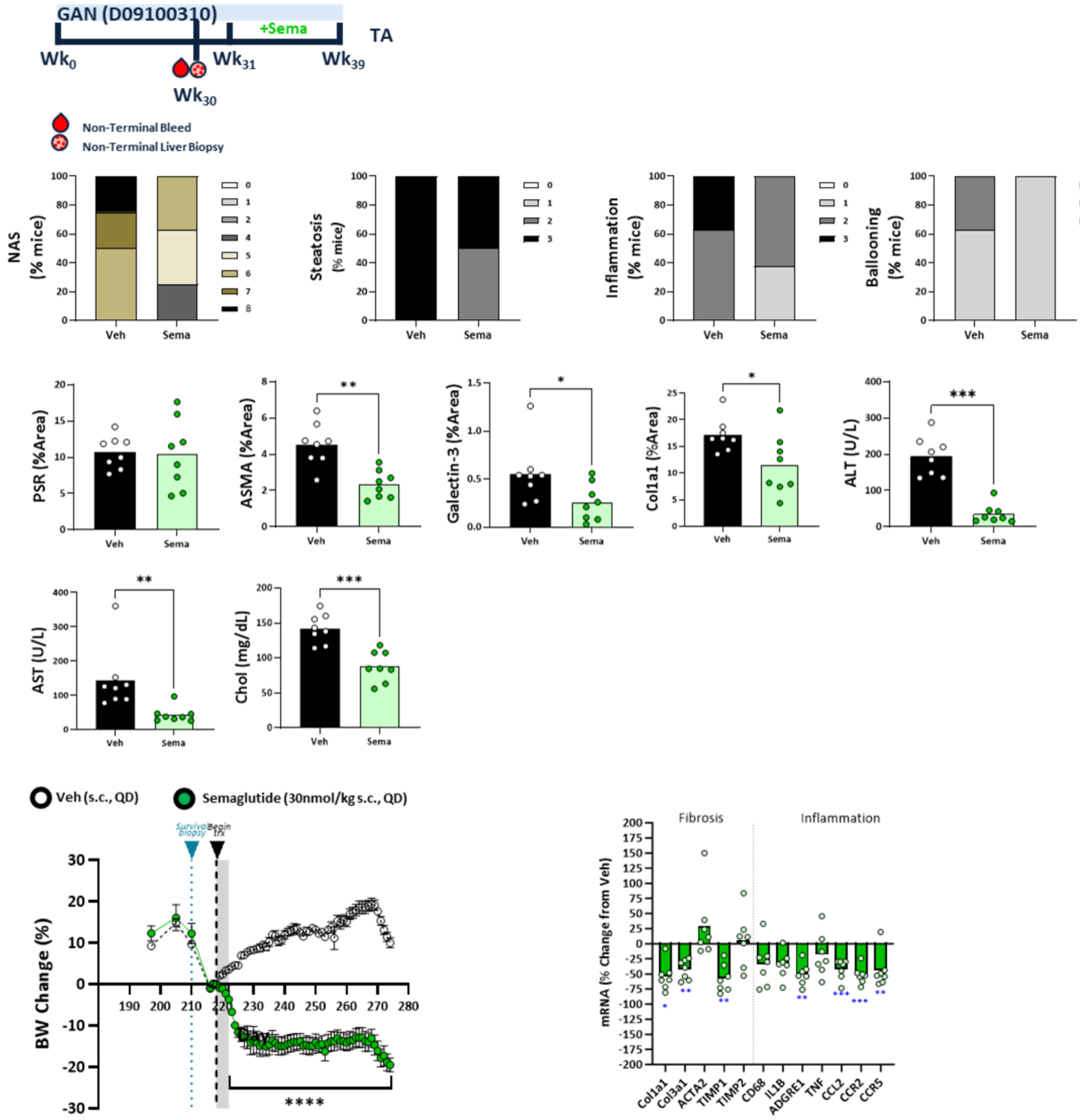

Supplementary Fig. 8. The effects of THRβ agonism (Resmetirom) on GAN diet induced steatosis, inflammation and fibrosis in male mice (C57BL/6J). (A) THRβ agonism improves steatosis, plasma cholesterol, and body weight . (B) Cytokine profiling and (C) mRNA profiling of liver of the mice treated with Resmetirom. Groups: -, Resm. Sample sizes: n = 8 per treatment. Statistical testing: Mann–Whitney Unpaired test : total cholesterol (Chol) p=0.0006; Cytokine profiling (KC/GRO p=0.0148); mRNA expression (*Acta2* p=0.0003, *Col1a2* p=0.0207, *Col4a2* p=0.0281, *Lgals1* p=0.0002, *Tgfb2* p=0.0104, *Saa1* p=0.0019, *Ccl2* p=0.0499). Age : 7weeks upon start of GAN diet.

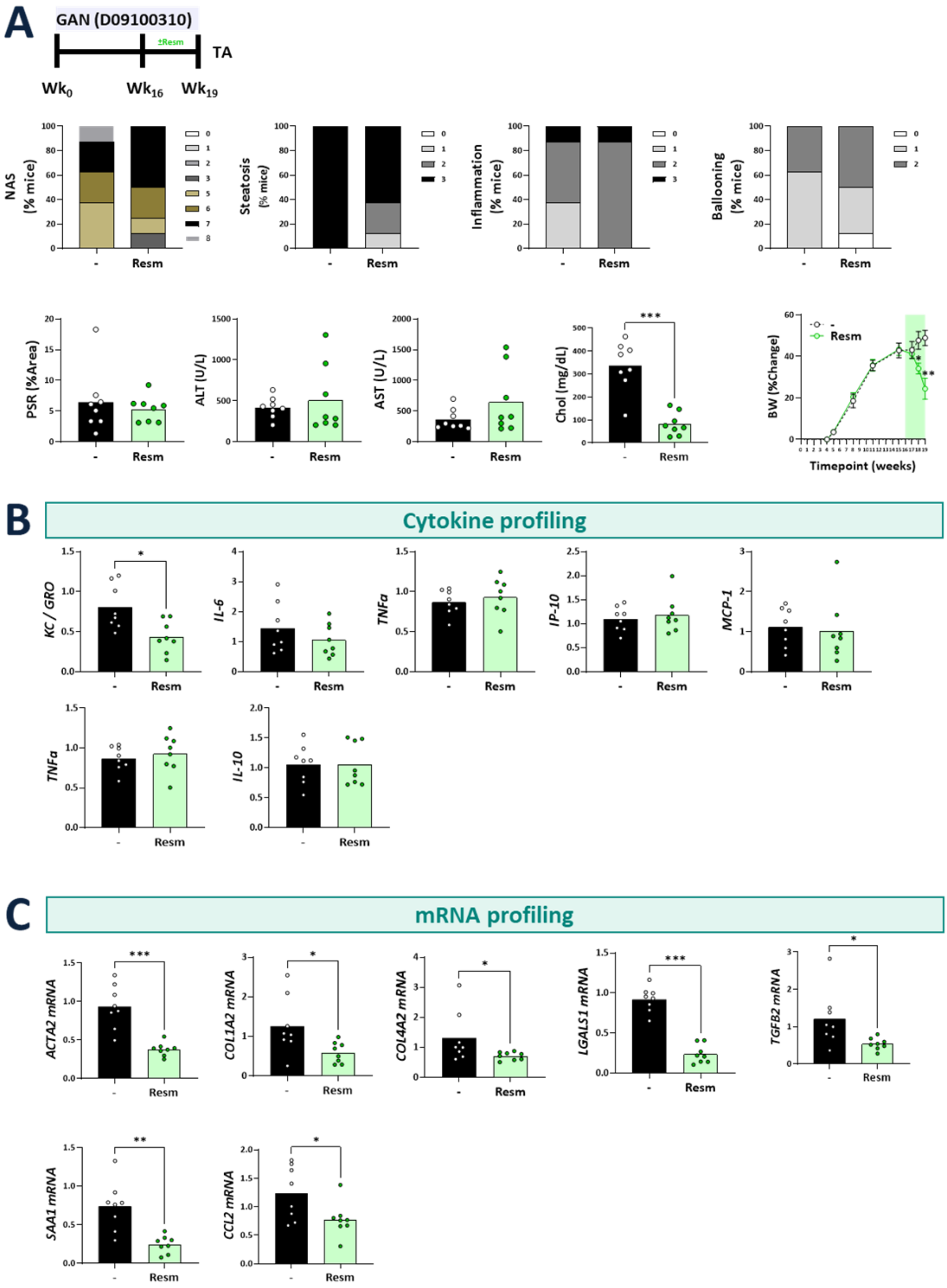

Supplementary Fig. 9A. The effects of ACC1/2 inhibition on CDAHFD induced steatosis, inflammation and fibrosis in male mice (C57BL/6N). (a) Representative histology and quantitative assessment showing that pharmacological inhibition of ACC1/2 (ACCi) reduces hepatic steatosis and ballooning in mice with diet-induced MASH. (b) Hepatic mRNA expression profiling of key fibrogenic and inflammatory markers in mice treated with ACCi. Groups: Untreated (-), Treated with ACC inhibitor (ACCi). Sample sizes: -, n = 10; ACCi, n = 11. Statistical testing: Mann–Whitney Unpaired test : ALT, p=0.0004; AST, p=0.0006; total cholesterol (Chol), mRNA expression (*Col3a1* p=0.0037, *Timp1* p=0.0048, *Timp2* p=0.0254, *Ccl2* p=0.0079). Age : 9-12weeks upon start of CDAHFD.

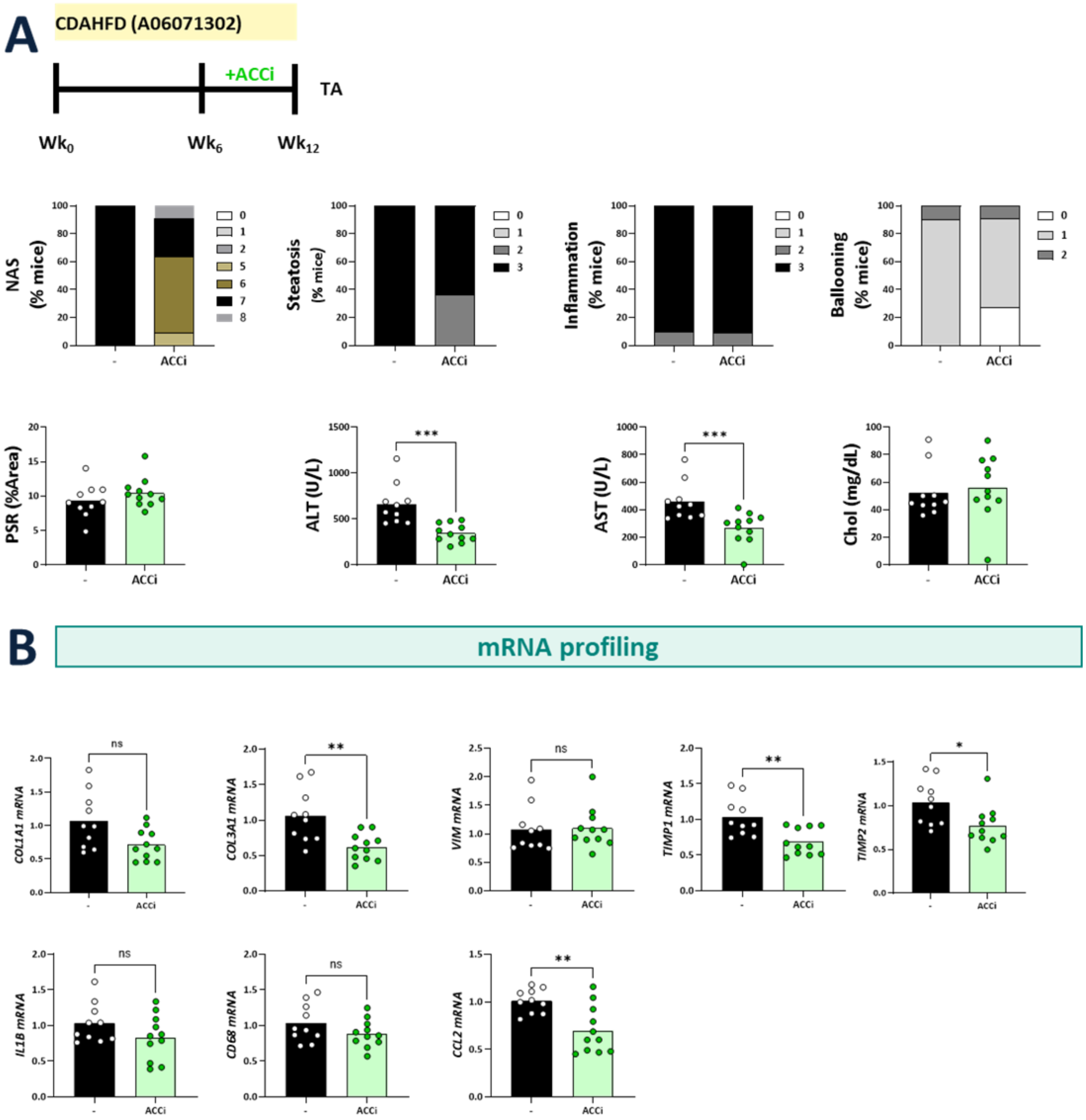

Supplementary Fig. 9B. The effects of ACC1/2 inhibition on GAN diet induced steatosis, inflammation and fibrosis in male mice (C57BL/6J). (a) Representative histology and quantitative assessment showing that pharmacological inhibition of ACC1/2 (ACCi) reduces hepatic steatosis and inflammation in mice with diet-induced MASH. (b) Hepatic mRNA expression profiling of key fibrogenic and inflammatory markers in mice treated with ACCi. Groups: Normal chow (NC), GAN diet (GAN), GAN diet plus ACC inhibitor (GAN + ACCi). Sample sizes: NC, n = 4; GAN, n = 6; GAN + ACCi, n = 7. Statistical testing: Mann–Whitney Unpaired test : AST, NC versus GAN, p=0.0381; total cholesterol (Chol), NC versus GAN, p=0.0095. Age : 8weeks upon start of GAN diet.

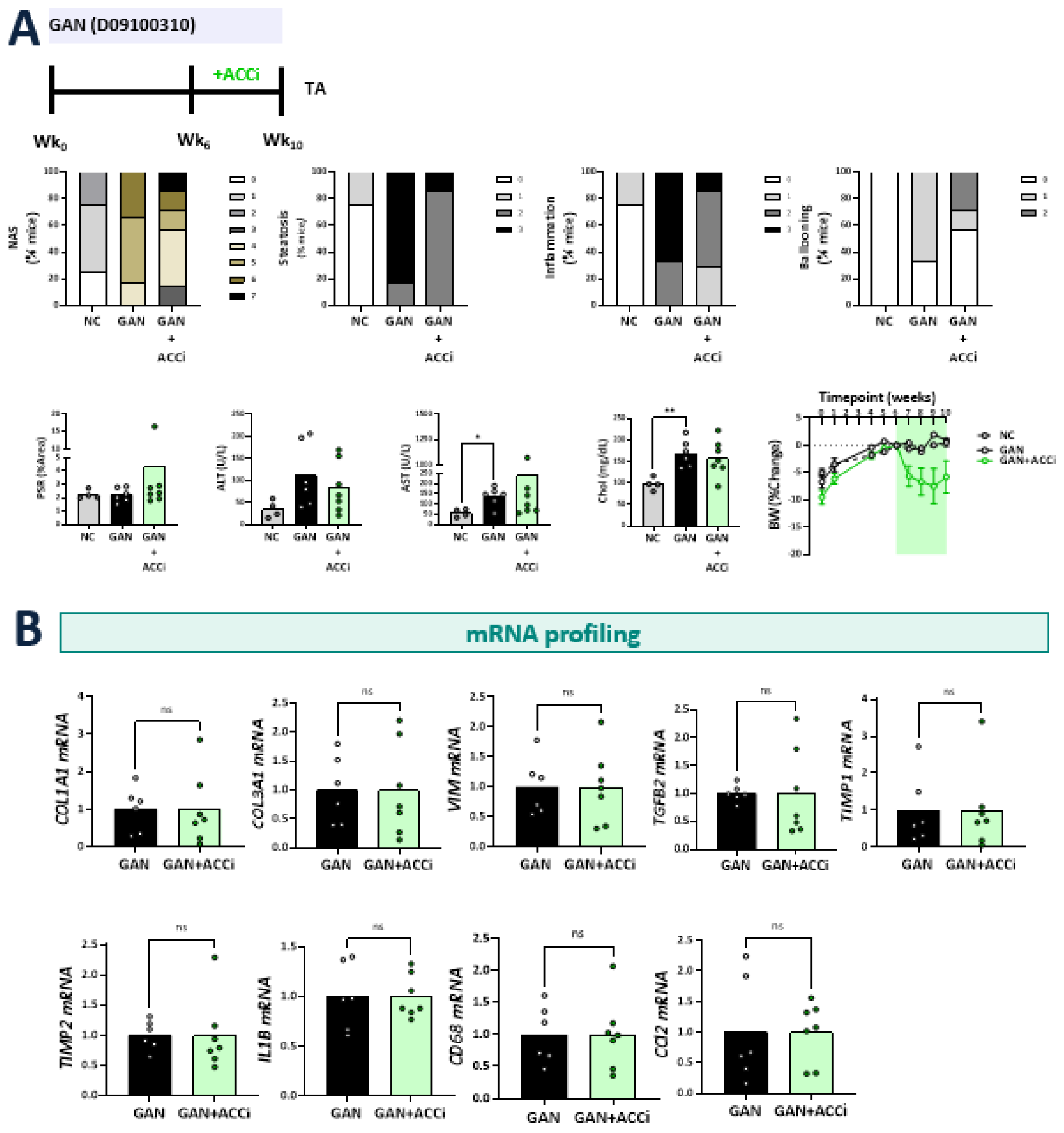

Supplementary Fig. 10. Comparisons SHG/TPEF with conventional histopathology for fibrosis and steatosis. (A-D) Comparisons of PSR with adjacent SHG, showing similar impression of fibrosis burden, (E-F) comparisons of MT and SHG on the same section shows enhanced visibility in SHG. (G) SHG signal has high correlation with collagen proportionate area (Pearson's R=0.92), calculated from the PSR-positive area normalized to tissue area). (H) SHG signal has a good correlation with the pathologist NASH-CRN fibrosis scores (Spearman's R=0.72). A high overlap between NASH CRN F2 and F3 can be appreciated, which highlight the needs for a more granular quantification compared to a categorical score. Furthermore, we observed that in animal models (e.g., CDAHFD model), fibrosis burden can continue increasing without a bridging being formed, preventing a definition of NASH-CRN fibrosis stage 3 to be achieved. (I) Good correlation between steatosis area quantified using AI and pathologist's steatosis score (Spearman's R-0.80). The high variability for AI-derived steatosis area quantification for steatosis score S3 highlights the challenges for pathologists to estimate steatosis area accurately (Steatosis score S3 is defined by steatosis area > 66%). Dataset: (G) N=210 (chow N=57, CDAHFD N=153 from week 2, 4, 8, 12, mixed of young and aged, TA), (H-I) N=265 (chow N=65, GAN N=47, CDAHFD N=153, mixed of young and aged, TA and TN; fibrosis F0: N=3, F1: N=100, F2: N=97, F3: N=63, F4: N=2; steatosis S0: N=54, S1: N=10. S2: N=20, S3: N=181). Statistics: (G) Pearson's correlation, (H-I) Spearman's correlation. Boxplot indicates median ± first quartile or third quartile. Age : 7weeks upon start of GAN diet (TA/TN) and CDAHFD (TN); 8weeks upon start of CDAHFD (TA); 77weeks upon start of CDAHFD (TA, Aged).

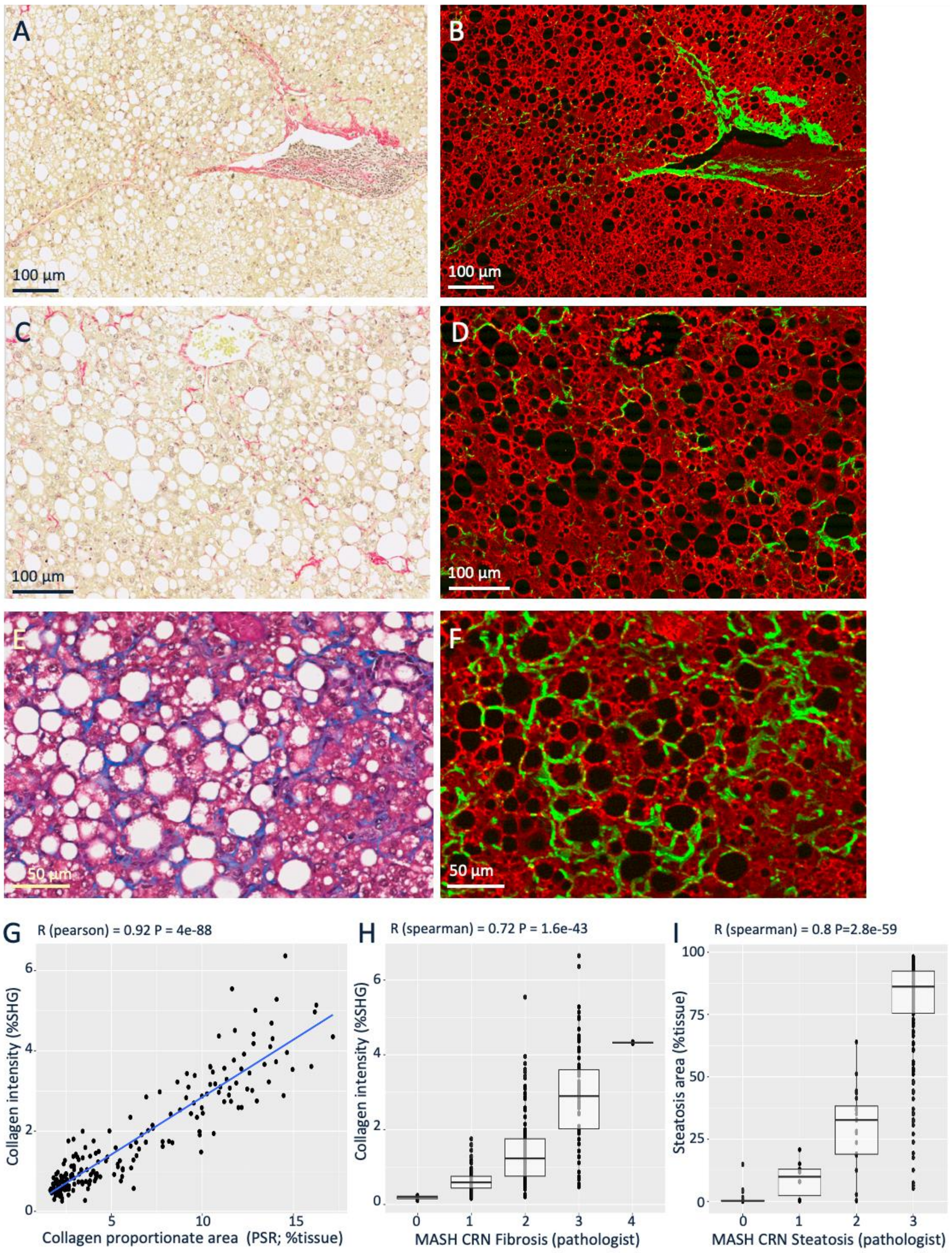

Supplementary Fig. 11. Performance of the deep learning model for vein segmentation and classification. (A) Example of the vein segmentation results, with (B) model performance achieving an average Intersection over Union (IoU) of 0.84. The subsequent classification model achieves a balanced accuracy of 90.3% with (C) an area under the curve of the Receiver-Operating curve (ROC AUC) of 0.95 and (D) Precision-Recall curve (PR AUC) of 0.92.

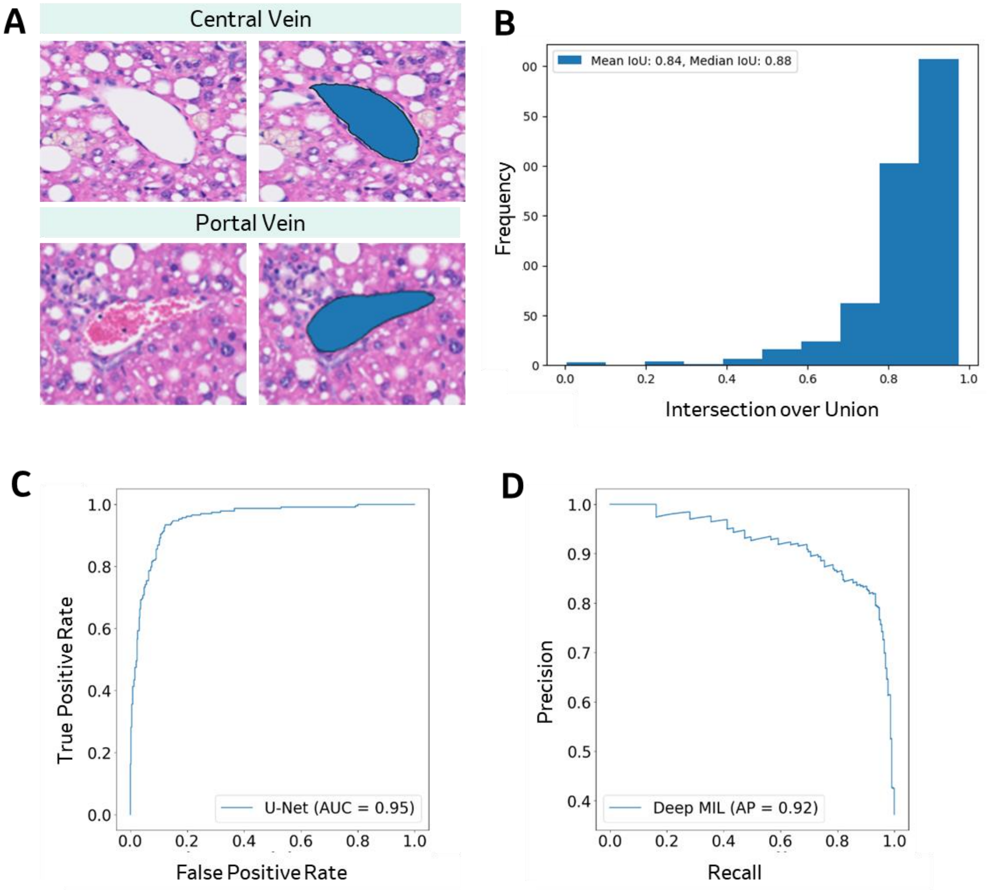

Supplementary Fig. 12. Determination of macrosteatosis and microsteatosis on H&E image. Macrovesicular steatosis are categorized as small droplet macrovesicular steatosis ("microsteatosis"), defined as lipid-droplets smaller than half of the cell size, and large-droplet macrovesicular steatosis ("macrosteatosis"), defined as lipid-droplets bigger than the half of the cell size and which displace the nucleus to the cell boundary [52,53]. The critical threshold of 15 µm was based on previous report [49] where a lipid-droplet size of 200 µm2 was used as a cutoff to determine macrosteatosis vs microsteatosis. [52,53]. The critical threshold of 15 µm was based on previous report [49] where a lipid-droplet size of 200 µm2 was used as a cutoff to determine macrosteatosis vs microsteatosis. The 200 µm2 corresponds to a diameter of 16 µm (assuming a perfect circle) or axis lengths of 15-17 µm (assuming an ellipse – a circle with a circularity index of 0.8). This data is supported in our observation: (A) randomly selected lipid-droplets with a diameter ranging from 5 µm to 30 µm from a WSI of a CDAHFD model. The detected lipid-droplets at 10 µm and below are smaller than half of a cell, wihle those above 16 µm are bigger than half the cell size and definitely macrosteatosis. While it is challenging to deduce visually for 14 µm, we can conclude that the threshold would lie between 14 µm and 16 µm. (B) The distributions of the lipid-droplets across different models showed two predominant clusters, corresponding to microsteatosis and macrosteatosis, separated around the diameter of 15 µm. Age : 7weeks upon start of GAN diet (TA/TN) and CDAHFD (TN); 8weeks upon start of CDAHFD (TA).

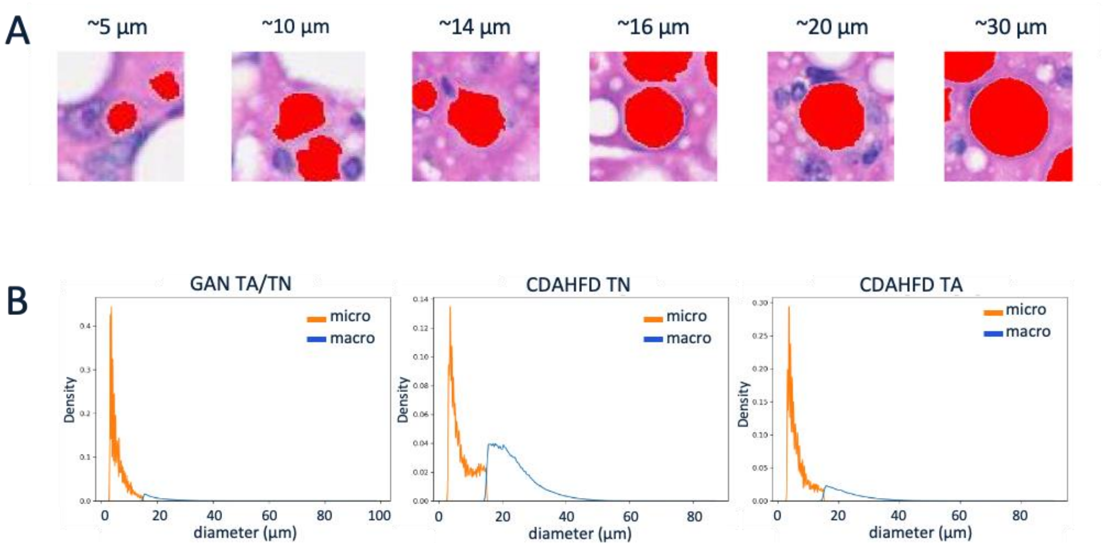

STATISTICAL ANALYSIS RESULTS

Figure 2B macrosteatosis area (GAN TA)

Bonferroni's multiple c omparisons test	Overall		CV		PS		PT	
	Summary	Adjusted P V alue	Summary	Adjusted P Value	Summary	Adjusted P Value	Summary	Adjusted P Value
4 vs. 12	ns	0.1249	ns	0.1934	ns	0.1114	ns	0.2598
4 vs. 23	**	0.0039	**	0.0029	**	0.004	*	0.0301
4 vs. 32	***	<0.0001	***	<0.0001	***	<0.0001	***	0.0004
12 vs. 23	ns	0.722	ns	0.3728	ns	0.8151	ns	>0.9999
12 vs. 32	***	<0.0001	***	<0.0001	***	<0.0001	*	0.0388
23 vs. 32	***	0.0003	**	0.002	***	0.0002	ns	0.3576

Figure 2C macrosteatosis area (GAN TN)

Bonferroni's multiple c omparisons test	Overall		CV		PS		PT	
	Summary	Adjusted P V alue	Summary	Adjusted P Value	Summary	Adjusted P Value	Summary	Adjusted P Value
4 vs. 12	ns	>0.9999	ns	>0.9999	ns	>0.9999	ns	0.4947
4 vs. 23	***	0.0006	***	0.0004	***	0.0002	**	0.0055
4 vs. 32	***	<0.0001	***	0.0002	***	<0.0001	**	0.0046
12 vs. 23	**	0.0062	**	0.0089	**	0.0019	ns	0.3804
12 vs. 32	***	0.0005	**	0.0033	***	0.0001	ns	0.2816
23 vs. 32	ns	>0.9999	ns	>0.9999	ns	>0.9999	ns	>0.9999

Figure 2D macrosteatosis area (CDAHFD TA)

Bonferroni's multiple c omparisons test	Overall		CV		PS		PT	
	Summary	Adjusted P V alue	Summary	Adjusted P Value	Summary	Adjusted P Value	Summary	Adjusted P Value
2 vs. 4	ns	0.0503	*	0.047	*	0.044	ns	0.0648
2 vs. 8	***	<0.0001	***	<0.0001	***	<0.0001	***	<0.0001
2 vs. 12	***	<0.0001	***	<0.0001	***	<0.0001	***	0.0001
4 vs. 8	***	0.0005	***	0.0002	***	0.0006	*	0.0246
4 vs. 12	*	0.0157	*	0.0102	*	0.0204	ns	0.1799
8 vs. 12	ns	>0.9999	ns	>0.9999	ns	>0.9999	ns	>0.9999

Figure 2E macrosteatosis area (CDAHFD TN)

Bonferroni's multiple c omparisons test	Overall		CV		PS		PT	
	Summary	Adjusted P V alue	Summary	Adjusted P Value	Summary	Adjusted P Value	Summary	Adjusted P Value
4 vs. 6	ns	0.168	*	0.0367	ns	0.3784	ns	>0.9999
4 vs. 8	ns	>0.9999	*	0.0453	ns	0.8145	ns	>0.9999
6 vs. 8	ns	0.0956	ns	>0.9999	*	0.0354	ns	>0.9999

Figure 2F macrosteatosis area (CDAHFD Aged TA)

Bonferroni's multiple c omparisons test	Overall		CV		PS		PT	
	Summary	Adjusted P V alue	Summary	Adjusted P Value	Summary	Adjusted P Value	Summary	Adjusted P Value
2 vs. 4	ns	>0.9999	ns	>0.9999	ns	>0.9999	ns	0.5571
2 vs. 8	ns	>0.9999	ns	>0.9999	ns	>0.9999	ns	0.1009
2 vs. 12	ns	0.4693	ns	0.8978	ns	0.8301	**	0.0076
4 vs. 8	ns	0.4589	ns	>0.9999	ns	0.8682	ns	>0.9999
4 vs. 12	*	0.0338	ns	0.2989	ns	0.0659	ns	0.3796
8 vs. 12	ns	>0.9999	ns	>0.9999	ns	>0.9999	ns	>0.9999

Figure 2B microsteatosis area (GAN TA)

Bonferroni's multiple c omparisons test	Overall		CV		PS		PT	
	Summary	Adjusted P Value	Summary	Adjusted P Value	Summary	Adjusted P Value	Summary	Adjusted P Value
4 vs. 12	ns	>0.9999	ns	>0.9999	ns	>0.9999	ns	0.3142
4 vs. 23	ns	0.4194	ns	0.0557	ns	0.5597	ns	0.111
4 vs. 32	ns	0.4343	*	0.0354	ns	0.6281	*	0.0381
12 vs. 23	ns	>0.9999	ns	0.8496	ns	>0.9999	ns	>0.9999
12 vs. 32	ns	>0.9999	ns	0.5796	ns	>0.9999	ns	>0.9999
23 vs. 32	ns	>0.9999	ns	>0.9999	ns	>0.9999	ns	>0.9999

Figure 2C microsteatosis area (GAN TN)

Bonferroni's multiple c omparisons test	Overall		CV		PS		PT	
	Summary	Adjusted P Value	Summary	Adjusted P Value	Summary	Adjusted P Value	Summary	Adjusted P Value
4 vs. 12	ns	0.0575	ns	0.0659	ns	0.0533	ns	0.4962
4 vs. 23	ns	0.0718	*	0.0227	ns	0.0683	ns	0.4701
4 vs. 32	ns	0.0575	*	0.0326	ns	0.0643	ns	0.2949
12 vs. 23	ns	>0.9999	ns	>0.9999	ns	>0.9999	ns	>0.9999
12 vs. 32	ns	>0.9999	ns	>0.9999	ns	>0.9999	ns	>0.9999
23 vs. 32	ns	>0.9999	ns	>0.9999	ns	>0.9999	ns	>0.9999

Figure 2D microsteatosis area (CDAHFD TA)

Bonferroni's multiple c omparisons test	Overall		CV		PS		PT	
	Summary	Adjusted P Value	Summary	Adjusted P Value	Summary	Adjusted P Value	Summary	Adjusted P Value
2 vs. 4	ns	>0.9999	ns	>0.9999	ns	>0.9999	ns	>0.9999
2 vs. 8	**	0.0011	*	0.0155	***	0.0008	*	0.0469
2 vs. 12	*	0.0392	ns	0.0935	*	0.0314	ns	0.2567
4 vs. 8	**	0.0025	*	0.0133	**	0.0027	*	0.0182
4 vs. 12	ns	0.078	ns	0.0813	ns	0.09	ns	0.1081
8 vs. 12	ns	>0.9999	ns	>0.9999	ns	>0.9999	ns	>0.9999

Figure 2E microsteatosis area (CDAHFD TN)

Bonferroni's multiple c omparisons test	Overall		CV		PS		PT	
	Summary	Adjusted P Value	Summary	Adjusted P Value	Summary	Adjusted P Value	Summary	Adjusted P Value
4 vs. 6	ns	0.0589	***	0.0001	***	0.0001	*	0.0191
4 vs. 8	ns	>0.9999	***	<0.0001	***	<0.0001	***	0.0004
6 vs. 8	ns	0.2583	*	0.0469	*	0.0469	ns	0.4238

Figure 2F microsteatosis area (CDAHFD Aged TA)

Bonferroni's multiple c omparisons test	Overall		CV		PS		PT	
	Summary	Adjusted P Value	Summary	Adjusted P Value	Summary	Adjusted P Value	Summary	Adjusted P Value
2 vs. 4	**	0.001	**	0.003	***	0.0008	ns	>0.9999
2 vs. 8	ns	0.138	*	0.011	ns	0.0889	ns	>0.9999
2 vs. 12	ns	>0.9999	ns	0.5003	ns	>0.9999	ns	0.4338
4 vs. 8	ns	0.33	ns	>0.9999	ns	0.3929	ns	>0.9999
4 vs. 12	**	0.0027	ns	0.4438	**	0.0032	*	0.0383
8 vs. 12	ns	0.2355	ns	>0.9999	ns	0.2257	ns	0.238

Figure 3D Inflam clusters (GAN TA)

Bonferroni's multiple comparisons test	CV		PS		PT	
	Summary	Adjusted P Value	Summary	Adjusted P Value	Summary	Adjusted P Value
4 vs. 12	ns	>0.9999	ns	>0.9999	ns	>0.9999
4 vs. 23	ns	>0.9999	ns	0.6758	ns	>0.9999
4 vs. 32	ns	>0.9999	ns	>0.9999	ns	>0.9999
12 vs. 23	ns	0.6701	ns	0.3244	ns	>0.9999
12 vs. 32	ns	0.9141	ns	0.9546	ns	>0.9999
23 vs. 32	ns	>0.9999	ns	>0.9999	ns	>0.9999

Figure 3E Inflam clusters (GAN TN)

Bonferroni's multiple comparisons test	CV		PS		PT	
	Summary	Adjusted P Value	Summary	Adjusted P Value	Summary	Adjusted P Value
4 vs. 12	ns	0.4316	ns	0.252	ns	0.0537
4 vs. 23	ns	>0.9999	ns	>0.9999	ns	>0.9999
4 vs. 32	ns	>0.9999	ns	>0.9999	ns	>0.9999
12 vs. 23	ns	0.9	ns	0.8035	ns	0.3944
12 vs. 32	ns	>0.9999	ns	>0.9999	ns	0.929
23 vs. 32	ns	>0.9999	ns	>0.9999	ns	>0.9999

Figure 3F Inflam cluster (CDAHFD TA)

Bonferroni's multiple comparisons test	CV		PS		PT	
	Summary	Adjusted P Value	Summary	Adjusted P Value	Summary	Adjusted P Value
2 vs. 4	ns	>0.9999	ns	0.4724	ns	0.1972
2 vs. 8	***	<0.0001	**	0.0019	*	0.0244
2 vs. 12	***	<0.0001	**	0.0018	ns	0.0906
4 vs. 8	***	0.0009	ns	0.2215	ns	>0.9999
4 vs. 12	***	0.0008	ns	0.2054	ns	>0.9999
8 vs. 12	ns	>0.9999	ns	>0.9999	ns	>0.9999

Figure 4F SHG (CDAHFD TN)

Bonferroni's multiple comparisons test	CV		PS		PT	
	Summary	Adjusted P Value	Summary	Adjusted P Value	Summary	Adjusted P Value
4 vs. 6	ns	>0.9999	ns	0.4885	ns	0.5734
4 vs. 8	ns	0.0732	***	0.0003	**	0.007
6 vs. 8	ns	0.0557	*	0.0106	ns	0.146

Figure 4G SHG (CDAHFD Aged TA)

Bonferroni's multiple comparisons test	CV		PS		PT	
	Summary	Adjusted P Value	Summary	Adjusted P Value	Summary	Adjusted P Value
2 vs. 4	ns	>0.9999	ns	0.7397	ns	>0.9999
2 vs. 8	**	0.0034	*	0.0214	ns	0.0917
2 vs. 12	***	<0.0001	**	0.0033	*	0.0244
4 vs. 8	**	0.0031	***	0.0003	*	0.0262
4 vs. 12	***	<0.0001	***	<0.0001	**	0.0077
8 vs. 12	**	0.0018	ns	>0.9999	ns	>0.9999

Figure 4C SHG (TA)

Bonferroni's multiple comparisons test	Overall		CV		PS		PT	
	Summary	Adjusted P Value	Summary	Adjusted P Value	Summary	Adjusted P Value	Summary	Adjusted P Value
4 vs. 12	ns	>0.9999	*	0.0448	ns	>0.9999	ns	>0.9999
4 vs. 23	ns	>0.9999	**	0.0073	ns	>0.9999	ns	0.4203
4 vs. 32	ns	0.8557	*	0.0152	**	0.0091	ns	0.4667
12 vs. 23	ns	>0.9999	ns	>0.9999	ns	>0.9999	ns	0.2954
12 vs. 32	ns	0.0661	ns	>0.9999	**	0.0026	ns	0.331
23 vs. 32	*	0.0452	ns	>0.9999	*	0.0127	ns	>0.9999

Figure 4D SHG (TN)

Bonferroni's multiple comparisons test	Overall		CV		PS		PT	
	Summary	Adjusted P Value	Summary	Adjusted P Value	Summary	Adjusted P Value	Summary	Adjusted P Value
4 vs. 12	ns	>0.9999	ns	0.8326	ns	>0.9999	ns	>0.9999
4 vs. 23	**	0.0015	ns	>0.9999	***	<0.0001	ns	0.5318
4 vs. 32	***	<0.0001	ns	>0.9999	***	<0.0001	*	0.0389
12 vs. 23	***	0.0007	ns	0.0668	***	<0.0001	ns	0.6587
12 vs. 32	***	<0.0001	ns	0.1475	***	<0.0001	*	0.044
23 vs. 32	*	0.0323	ns	>0.9999	**	0.0017	ns	>0.9999

Figure 4E SHG (CDAHFD TA)

Bonferroni's multiple comparisons test	Overall		CV		PS		PT	
	Summary	Adjusted P Value	Summary	Adjusted P Value	Summary	Adjusted P Value	Summary	Adjusted P Value
2 vs. 4	ns	>0.9999	ns	>0.9999	ns	>0.9999	ns	>0.9999
2 vs. 8	***	0.0004	ns	0.9245	***	<0.0001	ns	0.8056
2 vs. 12	***	<0.0001	ns	>0.9999	***	<0.0001	ns	0.3995
4 vs. 8	**	0.0041	ns	>0.9999	**	0.0012	ns	>0.9999
4 vs. 12	***	<0.0001	ns	>0.9999	***	<0.0001	ns	0.8307
8 vs. 12	***	<0.0001	ns	0.8822	***	<0.0001	ns	>0.9999

Figure 4F SHG (CDAHFD TN)

Bonferroni's multiple comparisons test	Overall		CV		PS		PT	
	Summary	Adjusted P Value	Summary	Adjusted P Value	Summary	Adjusted P Value	Summary	Adjusted P Value
4 vs. 6	ns	>0.9999	ns	0.5591	ns	0.0956	ns	0.1276
4 vs. 8	***	<0.0001	ns	0.1183	***	<0.0001	ns	>0.9999
6 vs. 8	***	<0.0001	ns	>0.9999	***	<0.0001	ns	0.3809

Figure 4G SHG (CDAHFD Aged TA)

Bonferroni's multiple comparisons test	Overall		CV		PS		PT	
	Summary	Adjusted P Value	Summary	Adjusted P Value	Summary	Adjusted P Value	Summary	Adjusted P Value
2 vs. 4	ns	>0.9999	ns	>0.9999	ns	>0.9999	ns	>0.9999
2 vs. 8	***	<0.0001	**	0.008	***	<0.0001	ns	0.0696
2 vs. 12	***	<0.0001	*	0.0272	***	<0.0001	**	0.0013
4 vs. 8	***	<0.0001	***	0.0002	***	<0.0001	**	0.0039
4 vs. 12	***	<0.0001	**	0.0011	***	<0.0001	***	<0.0001
8 vs. 12	**	0.0054	ns	>0.9999	**	0.0024	ns	0.5226

Figure 5C Steatosis/fibrosis colocalization (TA)

Bonferroni's multiple comparisons test	Overall		Colocalized fibrosis		Non-colocalized fibrosis	
	Summary	Adjusted P Value	Summary	Adjusted P Value	Summary	Adjusted P Value
4 vs. 12	ns	>0.9999	ns	>0.9999	*	0.042
4 vs. 23	ns	>0.9999	ns	>0.9999	**	0.0025
4 vs. 32	ns	0.8557	**	0.0023	**	0.0031
12 vs. 23	ns	>0.9999	ns	>0.9999	ns	>0.9999
12 vs. 32	ns	0.0661	**	0.0027	ns	>0.9999
23 vs. 32	*	0.0452	**	0.0089	ns	>0.9999

Figure 5D Steatosis/fibrosis colocalization (TN)

Bonferroni's multiple comparisons test	Overall		Colocalized fibrosis		Non-colocalized fibrosis	
	Summary	Adjusted P Value	Summary	Adjusted P Value	Summary	Adjusted P Value
4 vs. 12	ns	>0.9999	ns	>0.9999	ns	>0.9999
4 vs. 23	**	0.0015	**	0.0032	ns	>0.9999
4 vs. 32	****	<0.0001	****	<0.0001	ns	>0.9999
12 vs. 23	***	0.0007	**	0.0054	ns	0.5204
12 vs. 32	****	<0.0001	****	<0.0001	ns	>0.9999
23 vs. 32	*	0.0323	***	0.0009	ns	>0.9999

Figure 5E Steatosis/fibrosis olocalization (CDAHFD TA)

Bonferroni's multiple comparisons test	Overall		Colocalized fibrosis		Non-colocalized fibrosis	
	Summary	Adjusted P Value	Summary	Adjusted P Value	Summary	Adjusted P Value
2 vs. 4	ns	>0.9999	ns	>0.9999	ns	>0.9999
2 vs. 8	***	0.0004	***	<0.0001	ns	>0.9999
2 vs. 12	***	<0.0001	***	<0.0001	*	0.039
4 vs. 8	**	0.0041	***	0.0002	ns	>0.9999
4 vs. 12	***	<0.0001	***	<0.0001	*	0.0256
8 vs. 12	***	<0.0001	***	<0.0001	ns	0.4355

Figure 5F Steatosis/fibrosis olocalization (CDAHFD TN)

Bonferroni's multiple comparisons test	Overall		Colocalized fibrosis		Non-colocalized fibrosis	
	Summary	Adjusted P Value	Summary	Adjusted P Value	Summary	Adjusted P Value
4 vs. 6	ns	>0.9999	ns	0.2128	ns	0.1984
4 vs. 8	***	<0.0001	***	<0.0001	ns	>0.9999
6 vs. 8	***	<0.0001	***	<0.0001	ns	0.2012

Figure 5E Steatosis/fibrosis olocalization (CDAHFD TA)

Bonferroni's multiple comparisons test	Overall		Colocalized fibrosis		Non-colocalized fibrosis	
	Summary	Adjusted P Value	Summary	Adjusted P Value	Summary	Adjusted P Value
2 vs. 4	ns	>0.9999	ns	>0.9999	ns	>0.9999
2 vs. 8	***	<0.0001	***	<0.0001	*	0.0138
2 vs. 12	***	<0.0001	***	<0.0001	***	<0.0001
4 vs. 8	***	<0.0001	***	<0.0001	**	0.0029
4 vs. 12	***	<0.0001	***	<0.0001	***	<0.0001
8 vs. 12	**	0.0054	*	0.0125	*	0.0381

Figure 5H Microsteatosis/fibrosis colocalization (GAN; TA)

Bonferroni's multiple comparisons test	Summary	Adjusted P Value
4 vs. 12	ns	>0.9999
4 vs. 23	ns	>0.9999
4 vs. 32	**	0.0038
12 vs. 23	ns	>0.9999
12 vs. 32	**	0.0039
23 vs. 32	*	0.0121

Figure 5M Macrosteatosis/fibrosis colocalization (GAN; TA)

Bonferroni's multiple comparisons test	Summary	Adjusted P Value
4 vs. 12	ns	>0.9999
4 vs. 23	ns	>0.9999
4 vs. 32	**	0.0034
12 vs. 23	ns	>0.9999
12 vs. 32	**	0.0038
23 vs. 32	*	0.0112

Figure 5I Microsteatosis/fibrosis colocalization (GAN; TN)

Bonferroni's multiple comparisons test	Summary	Adjusted P Value
4 vs. 12	ns	>0.99
4 vs. 23	**	0.002
4 vs. 32	***	<0.001
12 vs. 23	**	0.004
12 vs. 32	***	<0.001
23 vs. 32	*	0.03

Figure 5N Macrosteatosis/fibrosis colocalization (GAN; TN)

Bonferroni's multiple comparisons test	Summary	Adjusted P Value
4 vs. 12	ns	>0.99
4 vs. 23	**	0.004
4 vs. 32	***	<0.001
12 vs. 23	**	0.006
12 vs. 32	***	<0.001
23 vs. 32	***	<0.001

Figure 5J Microsteatosis/fibrosis colocalization (CDAHFD; TA)

Bonferroni's multiple comparisons test	Summary	Adjusted P Value
2 vs. 4	ns	>0.99
2 vs. 8	ns	0.11
2 vs. 12	***	<0.001
4 vs. 8	ns	>0.99
4 vs. 12	***	<0.001
8 vs. 12	***	<0.001

Figure 5O Macrosteatosis/fibrosis colocalization (CDAHFD; TA)

Bonferroni's multiple comparisons test	Summary	Adjusted P Value
2 vs. 4	ns	>0.99
2 vs. 8	***	<0.001
2 vs. 12	***	<0.001
4 vs. 8	***	<0.001
4 vs. 12	***	<0.001
8 vs. 12	***	<0.001

Figure 5S Inflammation//fibrosis colocalization (CDAHFD; TA)

Bonferroni's multiple co mparisons test	Summary	Adjusted P Value
2 vs. 4	ns	>0.99
2 vs. 8	**	0.004
2 vs. 12	***	<0.001
4 vs. 8	*	0.02
4 vs. 12	***	<0.001
8 vs. 12	ns	0.08

Figure 5K Microsteatosis/fibrosis colocalization (CDAHFD; TN)

Bonferroni's multiple comparisons test	Summary	Adjusted P Value
4 vs. 6	ns	0.19
4 vs. 8	ns	>0.99
6 vs. 8	ns	0.51

Figure 5P Macrosteatosis/fibrosis colocalization (CDAHFD; TN)

Bonferroni's multiple comparisons test	Summary	Adjusted P Value
4 vs. 6	ns	0.13
4 vs. 8	***	<0.001
6 vs. 8	***	<0.001

Figure 5T Inflammation//fibrosis colocalization (CDAHFD; TN)

Bonferroni's multiple co mparisons test	Summary	Adjusted P Value
4 vs. 6	ns	>0.99
4 vs. 8	***	<0.001
6 vs. 8	***	<0.001

Figure 5L Microsteatosis/fibrosis colocalization (CDAHFD; Aged TA)

Bonferroni's multiple comparisons test	Summary	Adjusted P Value
2 vs. 4	ns	>0.99
2 vs. 8	***	<0.001
2 vs. 12	***	<0.001
4 vs. 8	***	<0.001
4 vs. 12	***	<0.001
8 vs. 12	**	0.003

Figure 5Q Macrosteatosis/fibrosis colocalization (CDAHFD; Aged TA)

Bonferroni's multiple comparisons test	Summary	Adjusted P Value
2 vs. 4	ns	>0.99
2 vs. 8	***	<0.001
2 vs. 12	***	<0.001
4 vs. 8	***	<0.001
4 vs. 12	***	<0.001
8 vs. 12	*	0.04

Figure 5U Inflammation//fibrosis colocalization (CDAHFD; Aged TA)

Bonferroni's multiple con	Summary	Adjusted P Value
2 vs. 4	ns	>0.99
2 vs. 8	***	<0.001
2 vs. 12	***	<0.001
4 vs. 8	**	0.001
4 vs. 12	***	<0.001
8 vs. 12	**	0.004

Figure 6F Pigment granuloma(CDAHFD; TA)

Bonferroni's multiple comparisons test	Summary	Adjusted P Value
2 vs. 4	ns	>0.99
2 vs. 8	**	0.001
2 vs. 12	***	<0.001
4 vs. 8	**	0.002
4 vs. 12	***	<0.001
8 vs. 12	***	<0.001

Figure 6G Pigment granuloma(CDAHFD; TN)

Bonferroni's multiple comparisons test	Summary	Adjusted P Value
4 vs. 6	*	0.03
4 vs. 8	***	<0.001
6 vs. 8	***	<0.001

Figure 6H Pigment granuloma(CDAHFD Aged; TA)

Bonferroni's multiple comparisons test	Summary	Adjusted P Value
2 vs. 4	ns	>0.9999
2 vs. 8	ns	>0.9999
2 vs. 12	ns	0.0649
4 vs. 8	ns	>0.9999
4 vs. 12	ns	0.233
8 vs. 12	ns	0.7697

Figure 6I mIF (CDAHFD TA)

Bonferroni's multiple comparisons test	CD44		CD68		CD11b		F4/80	
	Summary	Adjusted P Value	Summary	Adjusted P Value	Summary	Adjusted P Value	Summary	Adjusted P Value
2 vs. 6	ns	>0.9999	ns	>0.9999	ns	>0.9999	ns	>0.9999
2 vs. 8	**	0.0013	**	0.0025	***	0.0009	*	0.01
6 vs. 8	**	0.0031	**	0.0026	**	0.0025	*	0.013

Figure 6K Pigment granuloma/fibrosis colocalization (CDAHFD; TA)

Bonferroni's multiple comparisons test	Summary	Adjusted P Value
2 vs. 4	ns	>0.99
2 vs. 8	ns	0.06
2 vs. 12	***	<0.001
4 vs. 8	ns	0.07
4 vs. 12	***	<0.001
8 vs. 12	***	<0.001

Figure 6L Pigment granuloma/fibrosis colocalization (CDAHFD; TN)

Bonferroni's multiple comparisons test	Summary	Adjusted P Value
4 vs. 6	ns	0.3
4 vs. 8	***	<0.001
6 vs. 8	***	<0.001

Figure 6M Pigment granuloma/fibrosis colocalization (CDAHFD Aged; TA)

Bonferroni's multiple comparisons test	Summary	Adjusted P Value
2 vs. 4	ns	>0.9999
2 vs. 8	*	0.0373
2 vs. 12	***	0.0007
4 vs. 8	ns	0.8428
4 vs. 12	*	0.0254
8 vs. 12	ns	0.5889

Figure 6Q Lipogranuloma F4/80 (GAN; TA)

Bonferroni's multiple comparisons test	Summary	Adjusted P Value
4 vs. 12	ns	>0.9999
4 vs. 23	ns	>0.9999
4 vs. 32	*	0.0125
12 vs. 23	ns	>0.9999
12 vs. 32	**	0.0024
23 vs. 32	**	0.0053

Figure 6R Lipogranuloma F4/80 (GAN; TN)

Bonferroni's multiple comparisons test	Summary	Adjusted P Value
4 vs. 12	ns	>0.9999
4 vs. 23	ns	>0.9999
4 vs. 32	ns	0.3628
12 vs. 23	ns	0.6319
12 vs. 32	*	0.0149
23 vs. 32	ns	0.5656

Semaglutide

Figure 7A Macrosteatosis

Fisher's LSD	Overall (Student's T-test)		CV		PS		PT	
	Summary	Pvalue	Summary	Pvalue	Summary	Pvalue	Summary	Pvalue
Untreated vs Sema	***	<0.0001	***	<0.0001	***	<0.0001	***	<0.0001

Figure 7B Microsteatosis

Fisher's LSD	Overall (Student's T-test)		CV		PS		PT	
	Summary	Pvalue	Summary	Pvalue	Summary	Pvalue	Summary	Pvalue
Untreated vs Sema	ns	0.2619	ns	0.3848	ns	0.1274	ns	0.5981

Figure 7C Inflammation clusters

Fisher's LSD	CV		PS		PT	
	Summary	Pvalue	Summary	Pvalue	Summary	Pvalue
Untreated vs Sema	ns	0.5499	ns	0.4242	***	0.0009

Figure 7D Fibrosis

Fisher's LSD	CV		PS		PT	
	Summary	Pvalue	Summary	Pvalue	Summary	Pvalue
Untreated vs Sema	ns	0.8992	ns	0.7979	ns	0.1143

Figure 7E Fibrosis colocalizing with steatosis

Student's T-Test	ns	0.05

Figure 7F Fibrosis colocalizing with microsteatosis

Student's T-Test	ns	0.6694

Figure 7G Fibrosis colocalizing with macrosteatosis

Student's T-Test	**	0.0077

Figure 7H Fibrosis not colocalizing with steatosis

Student's T-Test	***	0.0008

Resmetirom

Figure 7I Macrosteatosis

Fisher's LSD	Overall (Student's T-test)		CV		PS		PT	
	Summary	Pvalue	Summary	Pvalue	Summary	Pvalue	Summary	Pvalue
Untreated vs Resm	***	0.0004	***	<0.0001	***	<0.0001	***	<0.0001

Figure 7J Microsteatosis

Fisher's LSD	Overall (Student's T-test)		CV		PS		PT	
	Summary	Pvalue	Summary	Pvalue	Summary	Pvalue	Summary	Pvalue
Untreated vs Resm	***	0.0003	***	<0.0001	***	0.0006	***	<0.0001

Figure 7K Inflammation clusters

Fisher's LSD	CV		PS		PT	
	Summary	Pvalue	Summary	Pvalue	Summary	Pvalue
Untreated vs Resm	ns	0.7147	ns	0.9397	ns	0.0703

Figure 7L Fibrosis

Fisher's LSD	CV		PS		PT	
	Summary	Pvalue	Summary	Pvalue	Summary	Pvalue
Untreated vs Resm	ns	0.4023	ns	0.7946	ns	0.2221

Figure 7M Fibrosis colocalizing with steatosis

Student's T-Test	*	0.0363

Figure 7N Fibrosis colocalizing with microsteatosis

Student's T-Test	*	0.016

Figure 7O Fibrosis colocalizing with macrosteatosis

Student's T-Test	*	0.0311

Figure 7P Fibrosis not colocalizing with steatosis

Student's T-Test	***	<0.0001

ACCi

Figure 7Q Macrosteatosis

Fisher's LSD	Overall (Student's T-test)		CV		PS		PT	
	Summary	Pvalue	Summary	Pvalue	Summary	Pvalue	Summary	Pvalue
Untreated vs ACCi	***	P<0.0001	***	<0.0001	***	<0.0001	***	<0.0001

Figure 7R Microsteatosis

Fisher's LSD	Overall (Student's T-test)		CV		PS		PT	
	Summary	Pvalue	Summary	Pvalue	Summary	Pvalue	Summary	Pvalue
Untreated vs ACCi	**	0.0036	ns	0.5346	***	<0.0001	ns	0.3093

Figure 7S Inflammation clusters

Fisher's LSD	CV		PS		PT	
	Summary	Pvalue	Summary	Pvalue	Summary	Pvalue
Untreated vs ACCi	ns	0.147	ns	0.8106	ns	0.6412

Figure 7T Fibrosis

Fisher's LSD	CV		PS		PT	
	Summary	Pvalue	Summary	Pvalue	Summary	Pvalue
Untreated vs ACCi	ns	0.887	**	0.0061	ns	0.1548

Figure 7U Fibrosis colocalizing with steatosis

Student's T-Test	ns	0.4483

Figure 7V Fibrosis colocalizing with microsteatosis

Student's T-Test	ns	0.7306

Figure 7W Fibrosis colocalizing with macrosteatosis

Student's T-Test	ns	0.2967

Figure 7X Fibrosis not colocalizing with steatosis

Student's T-Test	***	0.0004
