# Supplementary material for: AI digital pathology as a key tool providing in-depth understanding of the progression and regression of MASH and fibrosis in male mouse models
Source: Nat Commun. 2026 Jul 22;17:6964. doi: 10.1038/s41467-026-73370-z (PMC13392390; doi:10.1038/s41467-026-73370-z)
Supplement: Supplementary file 2 — Reporting summary [file 41467_2026_73370_MOESM2_ESM.pdf]

nature portfolio

Corresponding author(s): Ashmita Saigal

Last updated by author(s): Apr 29, 2026

Reporting Summary

Nature Portfolio wishes to improve the reproducibility of the work that we publish. This form provides structure for consistency and transparency in reporting. For further information on Nature Portfolio policies, see our Editorial Policies and the Editorial Policy Checklist.

Statistics

For all statistical analyses, confirm that the following items are present in the figure legend, table legend, main text, or Methods section.

n/a	Confirmed	
☐	☒	The exact sample size (*n*) for each experimental group/condition, given as a discrete number and unit of measurement
☐	☒	A statement on whether measurements were taken from distinct samples or whether the same sample was measured repeatedly
☐	☒	The statistical test(s) used AND whether they are one- or two-sided<br>*Only common tests should be described solely by name; describe more complex techniques in the Methods section.*
☒	☐	A description of all covariates tested
☒	☐	A description of any assumptions or corrections, such as tests of normality and adjustment for multiple comparisons
☐	☒	A full description of the statistical parameters including central tendency (e.g. means) or other basic estimates (e.g. regression coefficient) AND variation (e.g. standard deviation) or associated estimates of uncertainty (e.g. confidence intervals)
☐	☒	For null hypothesis testing, the test statistic (e.g. *F*, *t*, *r*) with confidence intervals, effect sizes, degrees of freedom and *P* value noted<br>*Give P values as exact values whenever suitable.*
☒	☐	For Bayesian analysis, information on the choice of priors and Markov chain Monte Carlo settings
☒	☐	For hierarchical and complex designs, identification of the appropriate level for tests and full reporting of outcomes
☐	☒	Estimates of effect sizes (e.g. Cohen's *d*, Pearson's *r*), indicating how they were calculated

Our web collection on statistics for biologists contains articles on many of the points above.

Software and code

Policy information about availability of computer code

Data collection	WSI was generated using Vectra Polaris (Akoya) for stained images, and Genesis(R)200 system (Histoindex) for SHG images.
Data analysis	Histoindex Artificial Intelligence Digital Pathology platform, HALO (Indica Lab), and in-house AI model and image analysis (available in : https://zenodo.org under DOI 10.5281/zenodo.18208001). Additionally, sensitivity analysis varying StarDist and SAM thresholds was performed to evaluate its relationship with False positive rate.

For manuscripts utilizing custom algorithms or software that are central to the research but not yet described in published literature, software must be made available to editors and reviewers. We strongly encourage code deposition in a community repository (e.g. GitHub). See the Nature Portfolio guidelines for submitting code & software for further information.

Data

Policy information about availability of data

All manuscripts must include a data availability statement. This statement should provide the following information, where applicable:

- Accession codes, unique identifiers, or web links for publicly available datasets
- A description of any restrictions on data availability
- For clinical datasets or third party data, please ensure that the statement adheres to our policy

Source data underlying all main and supplementary figures and tables, including processed metabolite and lipid abundance values, are provided in the Source Data Files as Microsoft Excel Files. Representative whole-slide images (WSIs) used to develop and evaluate the algorithms, as well as the intermediate files, are available

via Zenodo repository (https;//doi.org/10.5281/zenodo.18208001), while the full set of WSIs can be made available upon reasonable request. The full set of WSIs underlying this study comprises histological data from 256 animals across multiple experimental conditions, with paired H&E and SHG images totalling approximately 3–4 GB per animal (overall ~750GB–1TB). This large size makes routine deposition in public repositories and standard download workflows impractical. Providing the data upon request allows us to ensure reliable transfer (e.g., via appropriate file-sharing platforms) and that recipients receive complete, usable datasets. Bulk RNA-seq data generated in this study have been deposited in NCBI's Gene Expression Omnibus (GEO) with GSE accession number GSE316023 (https://www.ncbi.nlm.nih.gov/geo/query/acc.cgi?acc=GSE316023). Lipidomic and metabolic profiling was performed by a third-party facility (Metabolon Inc.) under a commercial service agreement. All processed and metabolomic data underlying the analyses and figures reported in the Supplementary Information are provided as Microsoft Excel files.

Research involving human participants, their data, or biological material

Policy information about studies with human participants or human data. See also policy information about sex, gender (identity/presentation), and sexual orientation and race, ethnicity and racism.

Reporting on sex and gender	N/A
Reporting on race, ethnicity, or other socially relevant groupings	N/A
Population characteristics	N/A
Recruitment	N/A
Ethics oversight	N/A

Note that full information on the approval of the study protocol must also be provided in the manuscript.

Field-specific reporting

Please select the one below that is the best fit for your research. If you are not sure, read the appropriate sections before making your selection.

☒ Life sciences ☐ Behavioural & social sciences ☐ Ecological, evolutionary & environmental sciences

For a reference copy of the document with all sections, see nature.com/documents/nr-reporting-summary-flat.pdf

Life sciences study design

All studies must disclose on these points even when the disclosure is negative.

Sample size	Sample sizes varied across experiments and are indicated in the corresponding figure legends and Methods. For each analysis, n values (number of animals) are clearly reported in the figure legends. Sample sizes were chosen based on prior experience with similar murine and omics studies and are standard for this type of experiment.
Data exclusions	Data points that are extreme outliers (lies above the 3*IQR) were excluded from the final analysis/plot/statistics; however, the values are included in the source data for each figure and indicated for exclusion.
Replication	All key findings were replicated in independent biological samples or technical replicates, as detailed in the Methods and Figure legends for each experiment.
Randomization	Animals were randomized based on body weight and baseline blood chemistry prior to enrolling to different treatment groups.
Blinding	No blinding was performed. Data collection and analysis were not blinded because group identities (e.g. control vs. treated) were objectively defined, and analyses (including omics and image quantification) were performed using standardized, automated, or algorithmic workflows that minimize observer bias.

Reporting for specific materials, systems and methods

We require information from authors about some types of materials, experimental systems and methods used in many studies. Here, indicate whether each material, system or method listed is relevant to your study. If you are not sure if a list item applies to your research, read the appropriate section before selecting a response.

Materials & experimental systems

n/a	Involved in the study	
☐	☒	Antibodies
☒	☐	Eukaryotic cell lines
☒	☐	Palaeontology and archaeology
☐	☒	Animals and other organisms
☒	☐	Clinical data
☒	☐	Dual use research of concern
☒	☐	Plants

Methods

n/a	Involved in the study	
☒	☐	ChIP-seq
☒	☐	Flow cytometry
☒	☐	MRI-based neuroimaging

Antibodies

Antibodies used	Macrophage panel : ULT30427 U-Vue-4-Plex (Ultivue) , T-cell panel : ULT30425 U-Vue 4-Plex (Ultivue), dilution 1:100
Validation	All primary antibodies were developed first with a 3,3'-diaminobenzidine (DAB) assay using an appropriate tissue control with the final DAB condition approved by licensed pathologist. Following approval in DAB, all primary antibodies were conjugated and stained in a singleplex immunofluorescence format utilizing the Insituplex assay with a minimum of 9 fluorescence conditions evaluated. Singleplex conditions were then approved by a licensed pathologist based on their qualitative concordance to the established DAB standard. Full panels, as outlined above, were then stained in a multiplexed format on appropriate tissue controls utilizing the Insituplex assay to ensure multiplexing did not impact functional performance of any singular biomarker. All development and testing outlined was performed by Vizgen.

Animals and other research organisms

Policy information about studies involving animals; ARRIVE guidelines recommended for reporting animal research, and Sex and Gender in Research

Laboratory animals	All animal experiments were performed in male mice (Mus musculus). Fig. 7Q-X and Supplementary Fig. 9A used C57BL/6N (Taconic) while remaining studies used C57BL/6J (Jackson Laboratories). Details of strain, sex, age, diet and number of animals used are provided in the Methods and Figure legends. Animals were housed in a controlled environment with ad-libitum access to food and water.
Wild animals	No wild animals were used in this study.
Reporting on sex	Both the sex and number of animals used in each experiment are reported in the Methods and Figure legends. Only male mice were used to minimize impact of hormones on disease progression.
Field-collected samples	No field-collected samples were used.
Ethics oversight	All animal experiments were approved and were performed in compliance with the Institutional Animal Care and Use Committee (IACUC) of Merck & Co., Inc., Rahway, NJ, USA (APS400180) and conducted in accordance with institutional and national guidelines for animal welfare.

Note that full information on the approval of the study protocol must also be provided in the manuscript.

Plants

Seed stocks	N/A
Novel plant genotypes	N/A
Authentication	N/A

April 2023
