# Supplementary material for: AI digital pathology as a key tool providing in-depth understanding of the progression and regression of MASH and fibrosis in male mouse models
Source: Nat Commun. 2026 Jul 22;17:6964. doi: 10.1038/s41467-026-73370-z (PMC13392390; doi:10.1038/s41467-026-73370-z)
Supplement: Supplementary file 3 — Transparent Peer Review file [file 41467_2026_73370_MOESM3_ESM.pdf]

nature portfolio

Peer Review File

AI digital pathology as a key tool providing in-depth understanding of the progression and regression of MASH and fibrosis in male mouse models

Corresponding Author: Ms Ashmita Saigal

This file contains all reviewer reports in order by version, followed by all author rebuttals in order by version.

Version 0:

Reviewer comments:

Reviewer #1

(Remarks to the Author)
This study presents a novel AI-based digital pathology pipeline to automatically quantify histopathological features of MASH, including the identification of central and portal veins. Subsequently, the authors characterize several murine MASH models using multi-omics analyses and the newly developed digital pathology approach. Furthermore, they report on the effects of clinically validated anti-MASLD compounds—semaglutide, resmetirom, and an ACC1 inhibitor—on histopathological features as detected by the novel digital pathology pipeline.
While the technical advance is noteworthy and the distinct histological transitions across the different murine models are interesting, there are several concerns regarding the assay's reliability and the translational relevance of the findings.
1. Given that the title emphasizes AI digital pathology, the first half of the manuscript, which focuses on omics analyses, may be disproportionately long.
2. The connections between the digital pathology findings and the omics analyses remain unclear. For instance, are there differences in accumulated metabolites between livers exhibiting predominantly macrosteatosis versus those with microsteatosis? The transcriptomic data might enable the authors to explore the molecular drivers underlying these histopathological differences.
3. In Figure 3, A substantial number of immune cells appear to be missed by the AI digital pathology pipeline, raising concerns about the accuracy of its quantification. For example, control mice show higher levels of inflammatory cells than the GAN model at week 23 (Figures 3D and 3E), which is inconsistent with clinical observations.
4. What is the biological significance of the pigmented granulomas shown in Figure 6?
5. In Figure 7, all three compounds increased fibrosis that was not co-localized with steatosis. How do the authors biologically interpret this finding?
6. Is the novel digital pathology pipeline applicable to human MASH specimens? Data on human samples would support that this technology might be useful for anti-MASH clinical trials.
7. Many AI-based digital pathology methods have been described previously. What is the specific novelty of this approach, and how does it improve upon existing technologies?
8. Please check the manuscript carefully for other typographical errors. In Figure S9, the phrase "GLP1 agonism improve" should be corrected to "GLP1 agonism improves", for instance.

(Remarks on code availability)

Reviewer #2

(Remarks to the Author)
The manuscript presents a comprehensive study on the application of AI-driven digital pathology to analyze murine models of Metabolic Dysfunction-Associated Steatohepatitis (MASH) and liver fibrosis. The authors have developed and validated a multi-modal AI pipeline for zone-specific analysis and spatial co-localization of key MASH features, providing insights beyond standard histology. The study is well-conducted, and the results are promising, highlighting the potential of AI in

improving the understanding and management of MASH and fibrosis.My concerns for this manuscript are as follows:

1. The authors stated that lipid droplets <15 μm were classified as microsteatosis and ≥15 μm as macrosteatosis. This critical threshold lacks biological justification. Why 15 μm? Was this threshold validated against established histological criteria or biochemical measures of lipid droplet toxicity?
2. The authors emphasized the AI's ability to distinguish micro/macrosteatosis but did not robustly link this distinction mechanistically to disease progression or treatment response beyond quantification. Does microsteatosis correlate more strongly with specific metabolic pathways or inflammatory signals than macrosteatosis in the omics data? The relevance of this AI-derived distinction for MASH pathophysiology or drug efficacy needs stronger justification.
3. The CDAHFD model is largely induced by choline deficiency, which impairs VLDL secretion and leads to steatosis, along with high fat. It's more of a rapid toxin/diet-induced model for steatohepatitis and fibrosis, unlike the GAN diet that better mimics human overnutrition. The current description might oversimplify the complex, partially toxic nature of CDAHFD's etiology, which could weaken the analysis of model differences and the colocalization analysis. It would be better to stress the distinct mechanisms of CDAHFD in the Introduction.
4. The inflammation detection model performs significantly worse on the GAN model (ROC AUC/PR AUC 0.82/0.55 at 20x) compared to CDAHFD (0.92/0.93 at 40x). Was the GAN model trained on lower-quality images or fewer annotations? Can the pipeline be reliably used across models if performance varies this drastically? This limitation needs critical discussion.
5. The study identifies granulomas and lipogranulomas as morphometric features of MASH. However, the significance of these structures in disease progression is not clearly established. Are these structures indicators of disease severity or specific stages of MASH progression? How do they correlate with other phenotypic metrics such as fibrosis and inflammation?
6. The significant increase in non-colocalized fibrosis with Resmetirom treatment (Fig 7L, Lines 337-340) is interpreted as potentially reflecting "tissue remodelling as part of resmetirom's efficacy." This interpretation is highly speculative and potentially misleading. An increase in fibrosis, even if non-colocalized, is generally considered detrimental in MASH. Proposing it as part of efficacy requires robust supporting evidence not provided here (e.g., markers of collagen degradation/remodeling, functional improvement independent of histology, comparison to known remodeling phases). Without this, the data could equally indicate a lack of efficacy or even a pro-fibrotic effect under certain conditions. This finding warrants a more cautious and critical interpretation.
7. The interpretation of microsteatosis plateauing while macrosteatosis increases due to "fusion" (Lines 217-218) is speculative. The data presented (size distribution) supports droplet growth but not fusion. While fusion is a potential mechanism, alternative explanations, such as preferential expansion of existing large droplets or differential susceptibility of hepatocyte zones, are not adequately considered.
8. The interpretation of steatosis progression following a "biochemical pathway" gradient from Zone 3 to Zone 1 and its implied similarity to human liver zonation requires caution. While human liver has metabolic zonation, the specific patterns and drivers in murine models under these diets may not perfectly recapitulate the human situation. The discussion overstates the direct clinical relevance of the murine zonal patterns observed.

(Remarks on code availability)

Reviewer #3

(Remarks to the Author)
The submitted article „AI digital pathology as a key tool providing in-depth understanding of the progression and regression of MASH and fibrosis in mice" by Dr Talukdar and colleagues provides and summarizes a broad variety of data on MASH and liver fibrosis in mouse models for human steatotic liver disease. As this work analyses pathological, computational, and pharmaceutical data, it is very comprehensive and forward-focused.
Especially regarding the pathological aspects of the study, we would like to raise concerns mainly from a methodological point of view.
• Were there any (liver-associated) background lesions in the examined mouse models detected and taken into account? Please state, how a reliable delineation of background lesions vs. experimentally induced pathologies was ensured (e.g., Fig. 3 inflammation vs extramedullary hematopoesis)
• With regard to feeding / 5 hour postprandial fasting period prior to experiment termination and mouse necropsy we would like to know if intra-hepatocyte glycogen storage was still present in the harvested liver tissues and how a reliable differentiation between cytoplasmic clearing due to glycogen vs. lipid storage was carried out.
• NASH-CRN scoring was developed as a histological scoring system for human liver biopsies. The methodology of NASH-CRN scores needs to be described in more detail incl. the individual scoring parameters. It needs to be explained how this human scoring system can be translated to the murine situation. According to our experience, comparative hepatocyte ballooning cannot be seen in mouse livers. Please provide a convincing histological example of hepatocyte ballooning, ideally species-specific images (mouse vs. human) in high magnification. And please explain in detail how the scoring was applied and comparison to the AI derived data was performed - also taking expert control into account.
• Shortly mention and explain the relationship of MASH and NASH, especially when referring to "NASH" scoring.
• As we know that mouse and human liver tissues and pathologies harbor species-specific differences (e.g., amount of physiologic fibrous connective tissue) we strongly recommend to show AI applicability on human tissue as well to enable for a broader translatability.
• According to line 447ff, two different slide/sections were assessed by the pathologist and AI. In order to validate SHG, it has to be shown how classic histopathological evaluation and results of SHG applied AI correlate on the same slide.
Also SHG should be explained in more detail.
• Line 209ff: Differentiation between macro- and micro-steatosis and the liver zones does not contrast with conventional

histopathology, as a pathologist can also differentiate zonal regions morphologically. The statement “This contrasts with conventional histopathology…” (line 210) is somehow misleading.
• State and discuss the major advantages and benefits of AI-pathology over a pathologist. What about time needed to establish and conduct the AI digital pathology workflow versus classic microscopic histopathology? How easily can this workflow be transferred to other experimental setups/pathological conditions?

Material and Methods
• Line 448: It is described that fixation was achieved by formalin fixation for 24 hours followed by ethanol at room temperature. Is this to be regarded as representative fixation standard (for diagnostic human samples)? How about the influence of different fixation protocols (e.g., formalin only, shorter or longer fixation) on liver tissue histomorphology and subsequent influence on AI digital pathology analysis?
• Line 454: Provide and specify used slide scanners and respective scanning resolutions
• Provide additional information on murine liver sampling and trimming, especially specify which liver lobes/localizations were examined. Where there any anatomical predilection sites/lobes and any macroscopic findings?

Figures
• Figure 3. Inflammation quantification using AI digital pathology
o Add scale bars to the HE images (A-C)
o A: According to the image detail shown, the focally distributed immune cells seem to be intra-parenchymal. How was this lesion characterized and differentiated from common background findings in mouse liver (e.g., extramedullary hematopoiesis)?
• Figure 4. Fibrosis quantification using AI digital pathology
o Please align maximum values on y-axis (C+D)
• Figure 5 and Figure 6
o The collagen deposition shown in Fig. 5 (H) and Fig. 6 (I) appears very mild/not verifiable. Can MT/PRS be provided to confirm and show fibrosis, in addition to HE, SHG/TPE? (Fig. 5 H and Fig. 6 I)

(Remarks on code availability)

Reviewer #4

(Remarks to the Author)
I co-reviewed this manuscript with one of the reviewers who provided the listed reports. This is part of the Nature Communications initiative to facilitate training in peer review and to provide appropriate recognition for Early Career Researchers who co-review manuscripts.

(Remarks on code availability)

Reviewer #5

(Remarks to the Author)
This study investigates murine models of metabolic dysfunction-associated steatohepatitis (MASH) and liver fibrosis using a multi-modal artificial intelligence (AI)-driven digital pathology pipeline. The study analyzes histology, blood chemistry, transcriptomics, lipidomics, and metabolomics to compare diet-induced models (e.g., Gubra Amylin NASH (GAN) and Choline Deficient Amino Acid-defined High Fat Diet (CDAHFD)) under different housing conditions (e.g., standard ambient (TA) or thermoneutral (TN)). The AI method quantifies zone-specific features such as micro- and macrosteatosis, inflammation, fibrosis, and granulomas, enabling colocalization analyses. Distinct model behaviors are described, with thermoneutral housing accelerating disease progression and certain diets producing more severe fibrosis. The manuscript further validates the AI approach in experiments with semaglutide, resmetirom, and an acetyl-CoA carboxylase inhibitor, highlighting differential treatment effects.

Specific comments:
1. Lines 122 and 124 describe that “housing animals at TN exacerbated disease progression and overall burden” and that “GAN-fed animals at TN reached Grade 3 steatosis in just 4 weeks, while those at TA took 8 weeks. By 16 weeks on GAN, 100% of TN-housed mice achieved the maximal score, compared to 28 weeks for TA-housed mice.”
In contrast, Line 148 states that “the GAN diet induced fewer transcriptional changes at TN than at TA by week 12” and that “transcriptional changes worsened between weeks 16 and 32 at TN, with the most significant difference at week 23.”
Based on this information, the pronounced gene expression transitions in GAN-fed animals under TN relative to TA appear to occur after 16 weeks, whereas TN-related disease progression is evident much earlier. This temporal mismatch warrants further explanation to reconcile the early phenotypic exacerbation with the delayed transcriptional divergence.

2. Lines 239 – 240: “The models achieved ROC AUC/PR AUC scores of 0.82/0.55 and 0.92/0.93, respectively.”
Please provide further clarification on the gold standards used to identify inflammatory cells in normal hepatocyte areas and steatotic regions.

3. Lines 279 – 280: “To assess whether liver fibrosis development in MASH models is metabolically driven, we quantified the colocalization of steatosis and fibrosis using SHG/TPEF images”.
Please provide a clear definition of “colocalization” of steatosis and fibrosis and how it is implemented in the AI model training process.

4. Lines 312 – 314: “Colocalization analysis indicated a correlation between pigmented granulomas and fibrosis development (Fig. 6I-J), suggesting a potential link to disease etiology.”
Please elaborate on this statement by providing supporting evidence on the potential causal links in etiology beyond the observed association.

5. Lines 488 – 489: “All Omics data was analyzed using R. Transcriptomics was analyzed with DESeq2, lipidomics was with package of lipidr while metabolomics utilized omu and MetaboAnalystR.” Please elaborate on the parameters used in these analyses, including sensitivity tests and multiple test correction conducted.

6. Lines 504 – 506: “AI-based image analysis was performed to identify liver zone-specific (Zone 3/CV, Zone 2/PS, Zone 1/PT) features comprising a total of 100 fibrosis features and 45 steatosis features, as well as the colocalization between fibrosis and steatosis.” Please provide further information on the AI model training process, including slide amount, internal or external validation, and categorization of the fibrosis/ steatosis features. Please include performance evaluation metrics of the AI model.

7. It would be helpful to compare the pros and cons of the multi-modal digital pathology in detecting liver zones in this study, compared to other existing AI models such as AIM-MASH, FibroNest, MorphoQuant, or Histoindex.

8. Could the authors expand on the potential applications of their murine-based AI digital liver pathology tool for assessing liver pathology in humans and other organisms? It would be helpful to incorporate this perspective in the Discussion section.

(Remarks on code availability)
The code provides a README file with instructions for installing and running the application.

Reviewer #6

(Remarks to the Author)
I co-reviewed this manuscript with one of the reviewers who provided the listed reports. This is part of the Nature Communications initiative to facilitate training in peer review and to provide appropriate recognition for Early Career Researchers who co-review manuscripts.

(Remarks on code availability)

Version 1:

Reviewer comments:

Reviewer #1

(Remarks to the Author)
I appreciate the authors’ efforts in addressing my previous concerns. However, I still have major concerns regarding the accuracy of newly developed AI model as specified below.

1. In response to my comment 3, the authors updated the inflammatory cell detection model using higher-resolution images. However, the validation of this model relies on a common conflation of correlation and accuracy. While they demonstrated a high correlation between the AI-based counts and the multiplexed immunofluorescence (mIF) gold standard (Supplementary Figure S4C), high correlation does not equate to absolute accuracy. Specifically, the AI model estimated only 300 immune cells in a section where mIF identified approximately 1,000. Because the model significantly underestimates the true cell count, it cannot yet be considered a reliable tool for accurate quantification.

2. The biological significance of pigmented granulomas remains speculative. To justify the inclusion of this feature in the model, the authors should provide data regarding its association with patient outcomes or therapeutic responses. Without establishing a clear clinico-pathological correlation, this AI-based detection tool lacks a defined purpose in a diagnostic or prognostic setting.

3. As noted by other reviewers, it is essential for the authors to demonstrate the applicability of this model to human MASH samples. At a minimum, providing preliminary data from a small cohort of human MASH cases would significantly strengthen the study by confirming that the model’s performance translates from experimental models to clinical pathology.

(Remarks on code availability)

Reviewer #2

(Remarks to the Author)
The authors have responded appropriately to my review comments and made changes to the revised paper. I have no additional comments.

(Remarks on code availability)

Reviewer #3

(Remarks to the Author)
Thank you to the authors for their well-written and comprehensive rebuttal letter incl. explanatory figures and the revised version of the manuscript. Responses are detailed, clear and all comments were addressed appropriately.

One major remaining point concerns the appropriate acknowledgement of the substantial scientific expert contribution of involved human and / or veterinary pathologists. Please add the initials in the M&M and author contribution sections.

(Remarks on code availability)

Reviewer #4

(Remarks to the Author)
I co-reviewed this manuscript with one of the reviewers who provided the listed reports. This is part of the Nature Communications initiative to facilitate training in peer review and to provide appropriate recognition for Early Career Researchers who co-review manuscripts.

(Remarks on code availability)

Reviewer #5

(Remarks to the Author)
The authors have addressed the points raised previously. Thank you.

(Remarks on code availability)
The code provides instructions for running the application.

Reviewer #6

(Remarks to the Author)
I co-reviewed this manuscript with one of the reviewers who provided the listed reports. This is part of the Nature Communications initiative to facilitate training in peer review and to provide appropriate recognition for Early Career Researchers who co-review manuscripts.

(Remarks on code availability)

Version 2:

Reviewer comments:

Reviewer #1

(Remarks to the Author)
The authors have responded appropriately to my review concerns. I have no additional comments.

(Remarks on code availability)

Open Access This Peer Review File is licensed under a Creative Commons Attribution 4.0 International License, which permits use, sharing, adaptation, distribution and reproduction in any medium or format, as long as you give appropriate credit to the original author(s) and the source, provide a link to the Creative Commons license, and indicate if changes were made.
In cases where reviewers are anonymous, credit should be given to 'Anonymous Referee' and the source.
The images or other third party material in this Peer Review File are included in the article's Creative Commons license, unless indicated otherwise in a credit line to the material. If material is not included in the article's Creative Commons license and your intended use is not permitted by statutory regulation or exceeds the permitted use, you will need to obtain permission directly from the copyright holder.
To view a copy of this license, visit https://creativecommons.org/licenses/by/4.0/

"AI digital pathology as a key tool providing in-depth understanding of the progression and regression of MASH and fibrosis in male mouse models"

Nature Communications manuscript NCOMMS-25-40823-A

Point-by-point answers to the reviewers' questions

Reviewer #1 (Remarks to the Author)

This study presents a novel AI-based digital pathology pipeline to automatically quantify histopathological features of MASH, including the identification of central and portal veins. Subsequently, the authors characterize several murine MASH models using multi-omics analyses and the newly developed digital pathology approach. Furthermore, they report on the effects of clinically validated anti-MASLD compounds—semaglutide, resmetirom, and an ACC1 inhibitor—on histopathological features as detected by the novel digital pathology pipeline. While the technical advance is noteworthy and the distinct histological transitions across the different murine models are interesting, there are several concerns regarding the assay's reliability and the translational relevance of the findings.

1. Given that the title emphasizes AI digital pathology, the first half of the manuscript, which focuses on omics analyses, may be disproportionately long.

Answer: Thank you for raising this valid concern. We have integrated the multi-omic findings that illuminate environmental influences on MASLD/MASH progression into the section "**Key insights from histopathology on environmental influences in MASLD/MASH progression**," which synthesizes the histopathology outputs with the most relevant omics evidence to strengthen translational relevance while maintaining focus on the AI pipeline. The following passages have been added :

"Bulk RNA sequencing corroborated these observations. GAN diet induced transcriptional changes at TN which worsened from week 16 to week 32, peaked at week 23 with 1305 Differentially expressed (DE) genes at TN vs. 836 DE genes at TA, followed by deceleration in changes thereafter (Fig. S1A). Additionally, KEGG and Gene Ontology (GO) enrichment analyses highlighted unique signatures for each housing conditions. While cardiac hypertrophy, fatty acid degradation, and inflammatory bowel disease were linked to TA, metabolically relevant signatures for Type II Diabetes, inflammasome activity, and fatty acid elongation were exclusive to TN (Fig. S1B). Notably, uniquely enriched pathways at TN are central to MASH pathogenesis suggestive of physiological relevance of housing conditions on disease etiology."

"Lipidomic analysis revealed that feeding on GAN diet upregulated short-chain, highly saturated TAGs and downregulated long-chain, low-saturation TAGs, particularly at TN (Fig. S1D) consistent with increased lipogenesis and reflective of hyperinsulinemia-driven SREBP-

1 activation. While differences in housing temperature were reflected modestly in metabolomic analysis, yet notable upregulation of sphingolipid synthesis (Fig. S1E), including 1-deoxysphinganine (m18:0) and sphingomyelin, explain dietary impact in contributing to systemic insulin resistance, dysregulated lipid accumulation, obesity-induced endothelial dysfunction, and atherosclerosis [3]. Several amino acid, drug metabolism and vitamin/co-factor related pathways were downregulated in liver (vitamin A, alanine, aspartate, glutamate, histidine, benzoate), and plasma (tyrosine, alanine, aspartate, lysine, phosphatidylglycerol (PG), phosphatidylethanolamine (PE))changes that can promote hepatic steatosis, ferroptosis, and disrupted redox homeostasis [4,5]. Top differentially expressed metabolites in the liver and plasma following GAN diet feeding are summarized in (Fig. S1F)."

We also included the following passage in the section **"AI-DP reveals temporal and spatial localization of steatosis development in different diet induced models of MASH and liver fibrosis"**

"De novo lipogenesis (DNL) favors production of saturated fatty acids with a relative loss of polyunsaturated fatty acids (PUFAs), consistent with our lipidomic and metabolomic data (Fig. S1D, F). In GAN-fed mice at weeks 12 and 23, microsteatosis is more advanced at TN than TA (Fig. 2B,C) paralleling stronger fatty-acid saturation and shorter chain lengths at TN in lipidomics (Fig. S1D) and downregulation of long-chain PUFAs in metabolomics (Fig. S1F). These patterns suggest that regions with prominent microsteatosis show biochemical signatures of enhanced DNL, altered fatty-acid remodeling, and metabolic features linked to lipotoxicity and steatohepatitis progression. Such mechanistic claims can be confirmed via spatial co-registration of molecular data with histologic AI segmentation."

2. The connections between the digital pathology findings and the omics analyses remain unclear. For instance, are there differences in accumulated metabolites between livers exhibiting predominantly macrosteatosis versus those with microsteatosis? The transcriptomic data might enable the authors to explore the molecular drivers underlying these histopathological differences.

Answer: We agree that linking the histologic patterns identified by DP to the omics data is important. In this study the omics (bulk RNA-seq, lipidomics, metabolomics) were used primarily as an initial, model-validation step and reproduced many previously reported biomarker changes, confirming the murine model. However, because those omics assays were performed on bulk liver (or plasma) tissue they do not provide the spatial resolution required to unambiguously assign specific transcripts, lipids or metabolites to macrosteatotic versus microsteatotic regions or to distinct hepatic zones. That limitation motivated our use of AI-based DP: the image analysis provides the spatial/zonation information and allowed us to quantify macro- and microsteatosis in periportal, midzonal and pericentral areas. Ideally, direct spatial molecular resolution could be achieved using one or more complementary approaches :

- Spatial transcriptomics (e.g., 10x Visium, NanoString GeoMx) to map gene expression to histologic zones.

- Laser-capture microdissection of macro- and microsteatotic regions followed by targeted lipidomic/metabolomic profiling or RNA-seq.
- Imaging mass spectrometry (MALDI or DESI) to spatially map lipids/metabolites.
- Single-nucleus or single-cell RNA-seq combined with in situ validation (IHC/ISH) to define cell types and pathways associated with each steatosis pattern.

The scope of this manuscript was specifically focused on developing and validating the AI-DP approach for evaluating histological progression of MASH and liver fibrosis in mice. An integrative analyses correlating zone-level histologic metrics (for example, percent macrosteatosis or microsteatosis in each zone) with sample-level omics features (differentially abundant metabolites, lipids, and transcript modules) can be performed but shall be fraught with limitations as mentioned above. However, we provided a brief qualitative discussion of this analysis in our response to Reviewer 2, Question 2 and included a paragraph in ***Sub-section "AI-DP reveals temporal and spatial localization of steatosis development in different diet induced models of MASH and liver fibrosis"***

"De novo lipogenesis (DNL) favors production of saturated fatty acids with a relative loss of polyunsaturated fatty acids (PUFAs), consistent with our lipidomic and metabolomic data (Fig. S1D, F). In GAN-fed mice at weeks 12 and 23, microsteatosis is more advanced at TN than TA (Fig. 2B,C) paralleling stronger fatty-acid saturation and shorter chain lengths at TN in lipidomics (Fig. S1D) and downregulation of long-chain PUFAs in metabolomics (Fig. S1F). These patterns suggest that regions with prominent microsteatosis show biochemical signatures of enhanced DNL, altered fatty-acid remodeling, and metabolic features linked to lipotoxicity and steatohepatitis progression. Such mechanistic claims can be confirmed via spatial co-registration of molecular data with histologic AI segmentation."

3. In Figure 3, A substantial number of immune cells appear to be missed by the AI digital pathology pipeline, raising concerns about the accuracy of its quantification. For example, control mice show higher levels of inflammatory cells than the GAN model at week 23 (Figures 3D and 3E), which is inconsistent with clinical observations.

Answer:

We appreciate the reviewer's concern regarding potential under-detection of immune cells by the AI pipeline and the apparent inconsistency at week 23.

Initially, the WSIs of the GAN model in Fig 3D-E was performed using the AI model tuned for 20x, while the CDAHFD model and the interventional studies were performed using the AI model tuned for 40x. As reported previously in the manuscript, the performance for 20x (ROC AUC/PR AUC 0.82/0.55) was not as high as that for 40x (ROC AUC/PR AUC 0.92/0.93). This can be attributed to fewer annotation at 20x (~1000 cells at 20x vs ~4000 cells at 40x) and relatively superior quality of annotations at 40x as pathologist could reliably identify the cells which they were "uncertain" about at 20x.

Following the comments by Reviewer 2 (Question #4), we re-cut, stained, and scanned the GAN samples at 40x, using the same conditions/setting as those for the CDAHFD.

For the validation of the model, we first conducted a correlation analysis of AI-DP based detection of inflammatory cell density and pathologists' inflammatory scores. These are board-certified pathologists who have extensive experience in rodent histology, performed targeted validation confirming the observations. The 40x model could differentiate inflammatory cells from oval and bile ductular cells showing a strong correlation with pathologist inflammation scores (Spearman's R = 0.82, P<0.001; added as panel A in Supplementary Figure S4).

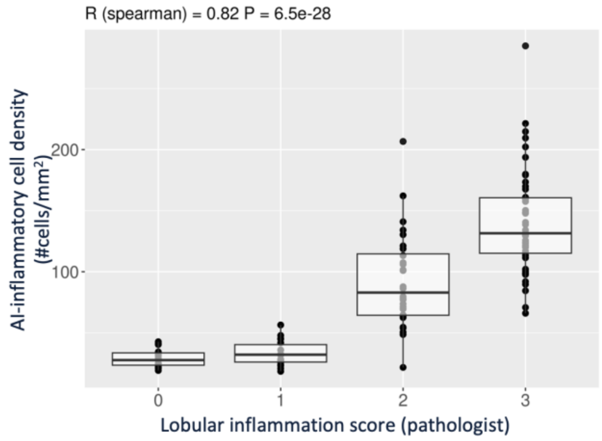

Supplementary Figure S4A. The correlation between AI-derived inflammatory cell density with MASH-CRN lobular inflammation score.

Next, we ran additional experiments using multiplexed-IF for an orthogonal validation. The multiplexed-IF (mIF) was performed on two different sections: one section with T-cell panel (i.e., CD3, CD4, CD8) and another section with macrophage panel (i.e., F4/80, CD68, CD11b, and CD44). H&E was performed on the same sections post-mIF. We assume that the sum of all markers (T-cells and macrophages) to be closer to the total inflammatory cell population that we can expect in the liver tissue. Indeed, we showed that the inflammatory cells being picked up by our AI model is highly correlated with orthogonal validation via mIF (Spearman's R = 0.83, P<0.001; added as panel C in Supplementary Figure S4). The T-cell panel and macrophage panel were performed on a different section; H&E was performed on the same section post-mIF. The H&E-based inflammatory cell density (x-axis) was the average of the inflammatory cells detected on the two corresponding H&E sections. This suggests that our model recapitulates inflammatory cells robustly. Furthermore, H&E and PSR images were qualitatively consistent with the recorded diet groups (Normal Chow and GAN) at TA and TN supporting the fidelity of the diet groups (Figure R1).

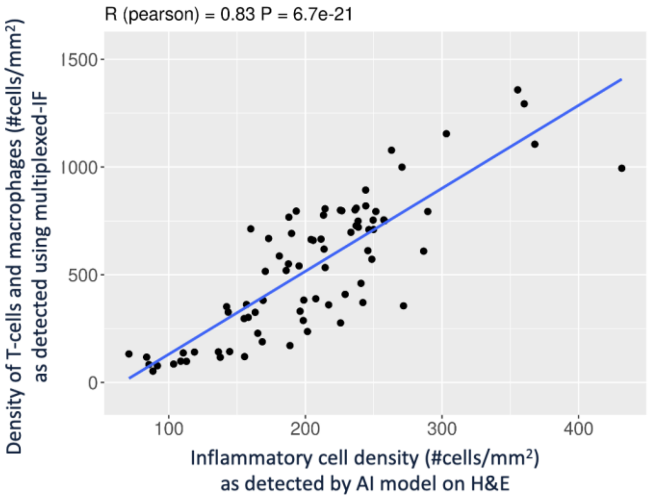

Supplementary Figure S4C. The correlation between inflammatory cell density as detected by AI model on H&E sections and the density of all T-cells (CD3+, CD4+, or CD8+) and macrophages (F4/80+, CD11b+, CD68+, or CD44+) as detected on multiplexed-IF.

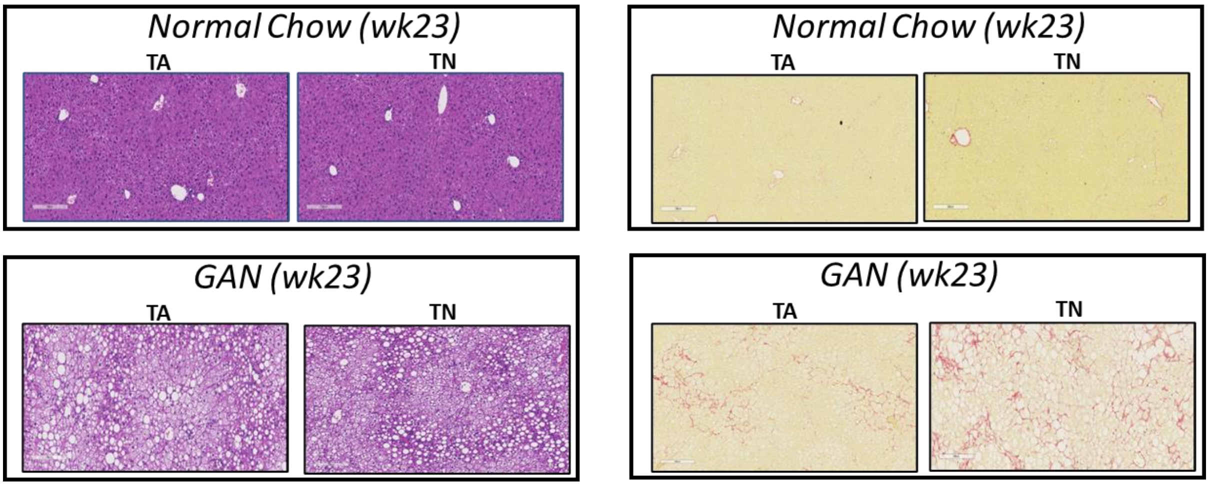

Figure R1. Representative H&E and PSR images for chow and GAN diet.

Finally, we aligned our quantification strategy with established clinical criteria. In NASH-CRN scoring, lobular inflammation is assessed by counting inflammatory foci per 200× field. To mirror this definition, we have now quantified inflammatory cell clusters normalized to tissue area rather than dispersed individual cell counts (Figures R2E, G, I, K, M; see below). This cluster-based metric is more comparable to the histopathologic standard and reduces bias from local tissue density and sectioning variability.

Our GAN-derived quantifications in both TA and TN arms converged with the pathologists' assessments (Spearman's R = 0.84, P<0.001; added as panel B in Supplementary Figure S4).

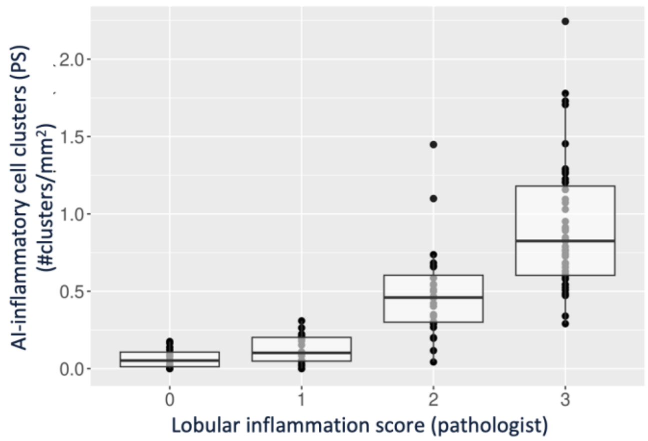

Supplementary Figure S4B. The correlation between AI-derived inflammatory cell clusters with MASH-CRN lobular inflammation score.

Notably, the variability observed at early timepoints (e.g., week 4 TN in GAN fed mice) was consistently captured by both methods (Fig S5 for conventional histopathology vs. FigR2F,G for AI DP), supporting the pipeline's sensitivity to biologically plausible fluctuations. Regarding the specific week 23 point, the apparent higher inflammation in controls relative to the GAN output (Figures R2D, 3F) reflects the distinction between cell-level detection (Figures R2D, F) and cluster-level scoring (Figures R2E, G). When evaluated on cluster counts normalized by area and cross-checked against pathologist scores (Fig S5), the week 23 findings became comparable with the other timepoints and the inflammation in GAN model was generally higher compared to chow.

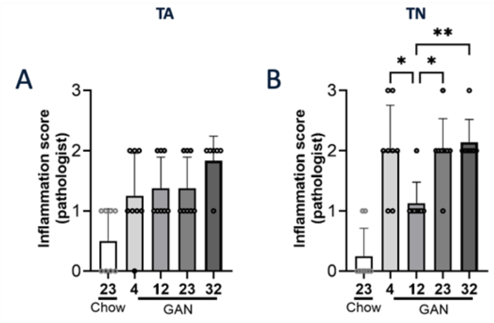

Supplementary Figure S5. NASH-CRN inflammation scores as evaluated by pathologists for GAN model at TA or TN.

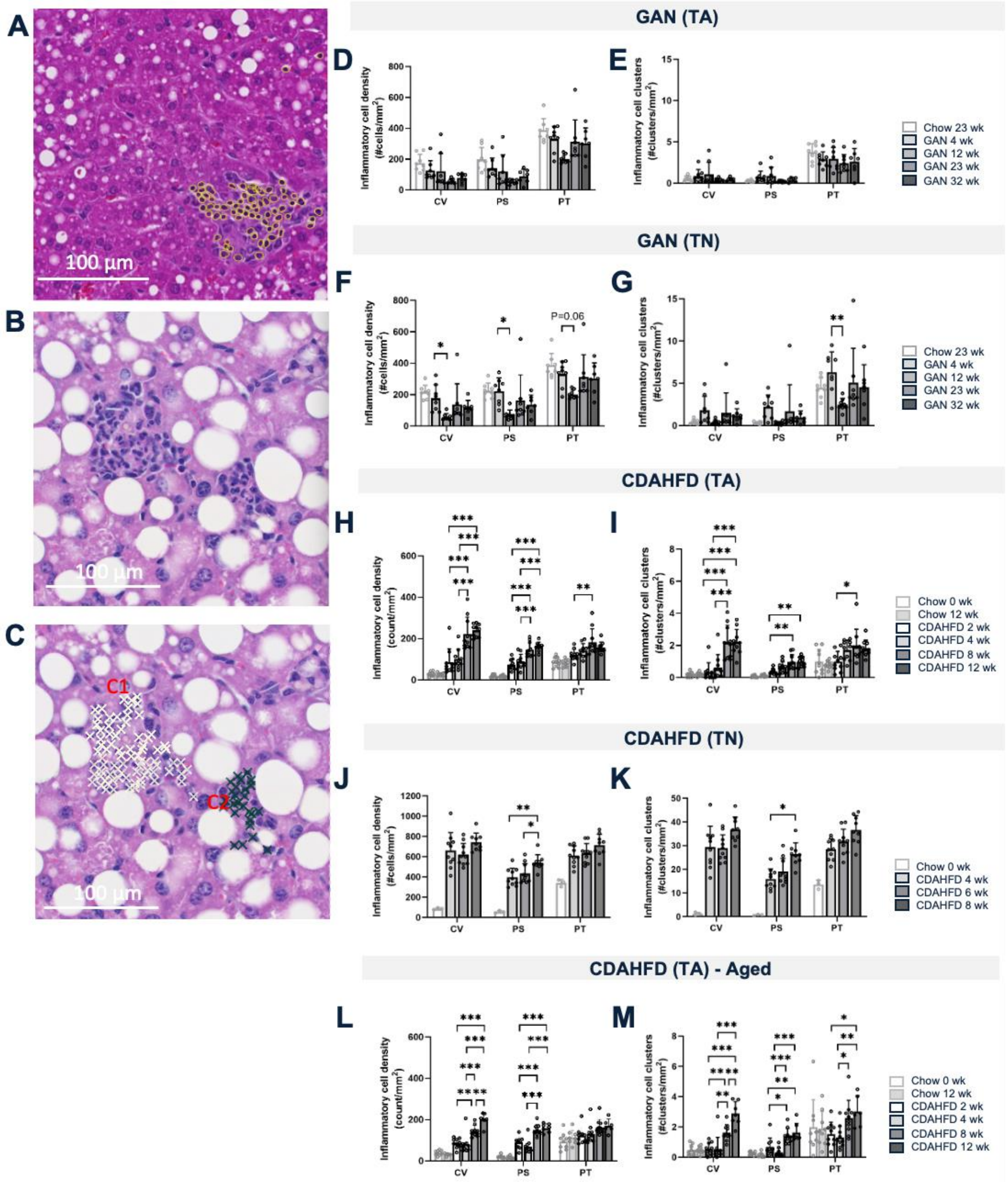

Fig. R2. (A) Illustration of detection of the individual inflammatory cells, (B) an example of two clusters of inflammatory cells, with the cells belonging to the same clusters are indicated by cross marks with the same color in (C). (D, F, H, J, L) Quantification of inflammatory cell density 40x and (E, G, I, K, M) the number of inflammatory foci 40x in (D-G) GAN model or (H-M) CDAHFD model, at (D-E, H-I, L-M) TA or (F-G, J-K) TN condition.

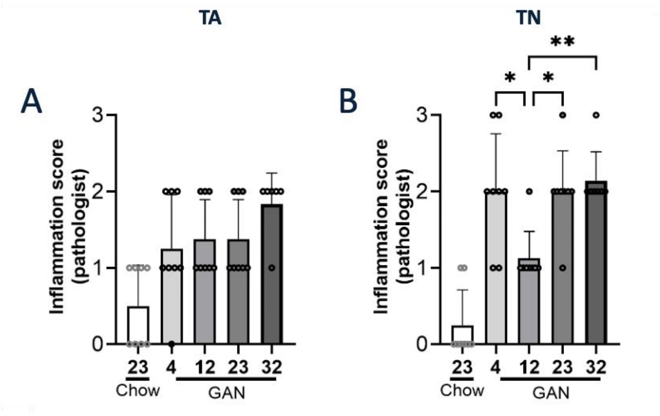

Supplementary Figure S5. Lobular inflammation score (NASH-CRN) by pathologist for the GAN diet at (A) TA and (B) TN housing condition.

The current threshold in the AI model for immune cells classification is chosen to minimize false positive rate at the expense of lower rate of detection of immune cells. Decreasing the detection threshold can increase the number of false positives which might compromise the quality of our interpretation. Overall, the dynamic range of inflammation is narrow in the GAN (as compared to the more aggressive CDAHFD model). Individual inflammatory cell counts may not clearly reflect real pattern shifts, and cluster-based analysis may be more robust to background noise from a few scattered inflammatory cells. Ideally, having a baseline biopsy for each animal might have provided individualized reference trajectories. However, we intentionally avoided baseline biopsies to prevent procedure-induced inflammation and to preserve the natural disease course.

Together, these steps address the concern about missed immune cells by showing that the AI pipeline's quantification agrees with expert histopathology and adheres to clinically grounded scoring criteria; we have added explicit methodological details and references to Figures 3 and S5 to improve clarity, and we have included representative overlays illustrating the cluster analysis (refer to Figure R2 C).

The following paragraph was added to the Results "Inflammation quantification using AI-DP":

"The model quantifies inflammatory clusters, like pathologist evaluation of inflammatory foci for assessing lobular inflammation (Fig. 3C) and shows a strong correlation with pathologist's inflammation scores (Spearman's R = 0.82 for inflammatory cell density; P<0.0001, Fig. S4A, Spearman's R = 0.84 for PS inflammatory cell clusters; P<0.001, Fig. S4B, and Spearman's R = 0.83 for inflammatory cells detected using multiplexed-IF for T-cell and myeloid panels; P<0.001, Fig. S4C) ."

"...compared to the aggressive CDAHFD model. This variability observed, especially at TN, was consistently captured by both, AI-DP and conventional histopathology (Fig. S5), supporting the AI pipeline's sensitivity to biologically plausible fluctuation. In the GAN model, inflammation was higher relative to chow when PS and CV regions were combined, areas pertinent to lobular inflammation, at TN (week-32: 2.8-fold vs. chow, P=0.006; Fig. 3E), but not at TA (week-32: P=0.98 vs. chow; Fig. 3D) consistent with the differential effect of housing temperature on disease progression."

4. What is the biological significance of the pigmented granulomas shown in Figure 6?

Answer:

The pigmented granulomas shown in Fig 6 were one of the two distinct types of hepatic granulomas observed in our murine MASH models (GAN diet–induced and CDAHFD-induced). The pigmented granulomas were specifically observed in the CDAHFD-induced models, where macrophages clustered as slightly pale, foamy, pigmented aggregates. In the GAN model, mononuclear cells and macrophages arranged around steatotic hepatocytes in a crown-like pattern (known as lipogranuloma).

When hepatocellular lipid release exceeds the clearance capacity of resident phagocytes, excess lipid is sequestered by macrophages, producing lipogranulomas [6]. Pigmented granulomas arise by a similar mechanism but show additional pigment accumulation (ceroid/lipofuscin) and often iron deposition [7]. These granulomas therefore reflect ongoing lipid handling/stress and macrophage activation (CD44+, CD68+) within the liver [6,8]. Such lesions are associated with chronic inflammation and have been linked to increased risk of progression to hepatocellular carcinoma (HCC) in chronic steatohepatitis models and human disease [9]. The anatomical location of granulomas can provide etiologic clues (for example, portal granulomas are more typical of primary biliary cholangitis, whereas viral-related granulomas tend to be parenchymal), but substantial overlap exists and location alone is not diagnostic [6]. Granulomas may regress if the underlying cause is treated; however, their fate depends on disease context, duration of injury and extent of fibrosis.

Notably, in our dataset, we observed that pigmented granuloma starts to correlate after inflammation reached a certain level – in this case after week 4 in the CDAHFD model (added as Panel B in Supplementary Figure S6), supporting the association with chronic inflammation. Pigmented granuloma prevalence correlated strongly with liver fibrosis in the CDAHFD model (added as Panel C in Supplementary Figure S6), supporting a link between pigment-laden macrophage aggregates and fibrogenic progression in this dietary model of MASH. Additionally, we also performed multiplexed-IF experiments using macrophage markers CD44, CD68, CD11b, and F4/80. Our data showed that CD44+ cells are the main infiltrates of pigmented granuloma, with 10-fold higher presence compared to the second majority which is CD68+ cells (added as Panel E in Figure6).

We added the biological explanation of pigmented granuloma and additional data in the **Results section "AI-DP for evaluation of pigmented granulomas and lipogranulomas in CDAHFD and GAN models of MASH and liver fibrosis", Figure 6,** and **Supplementary Figure S6**:

"We temporally evaluated pigmented granulomas using AI-DP (Fig. 6F-H), noting an increase in lesion area over time. In the CDAHFD model, factors such as age (2.1-fold higher in aged vs. young mice at week 8, P=0.015; Fig 6F and 6H) and housing conditions (3-fold higher at TN vs. TA at week 8, P<0.001; Fig 6F-G) influenced granuloma growth. Pigment granuloma starts to correlate with inflammation after week 4 (Pearson's R=0.51, P=0.016, Supplementary Fig. S6B), supporting the association with chronic inflammation [7]. We observed an increasing

time-dependent prevalence of CD44+ cells in the pigmented granuloma, which was about 8-fold at week 8 compared to CD68+ cells as the second highest population within the pigmented granuloma (Fig. 6E, I). This supports previous reports that granulomas reflect ongoing lipid handling/stress and macrophage activation (specifically CD44+ and CD68+) within the liver [6,8]. Notably, pigmented granuloma prevalence correlated strongly with liver fibrosis (Pearson's R=0.75, P<0.001, Supplementary Fig. S6C), which is supported by the colocalization analysis between pigmented granuloma and fibrosis (Fig. 6J-M)."

Addition to Figure 6:

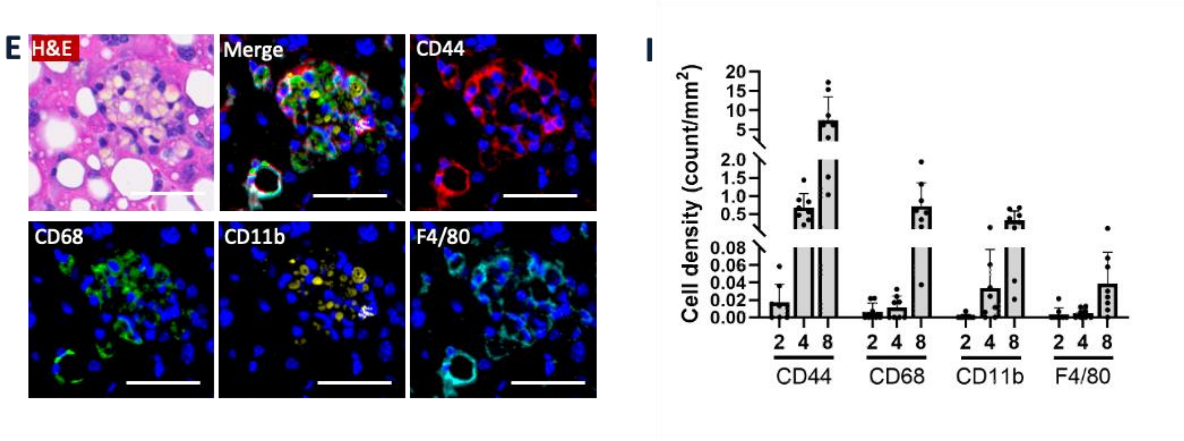

Figure 6. (E) Multiplexed-IF for macrophage panel (CD44,CD68, CD11b, F4/80), along with the H&E on the same section, further demonstrates the presence of macrophages in the pigmented granuloma. (I) Contribution of the different macrophages to the pigmented granuloma at 2,4,8 weeks on CDAHFD.

Addition to Supplementary Figure S6:

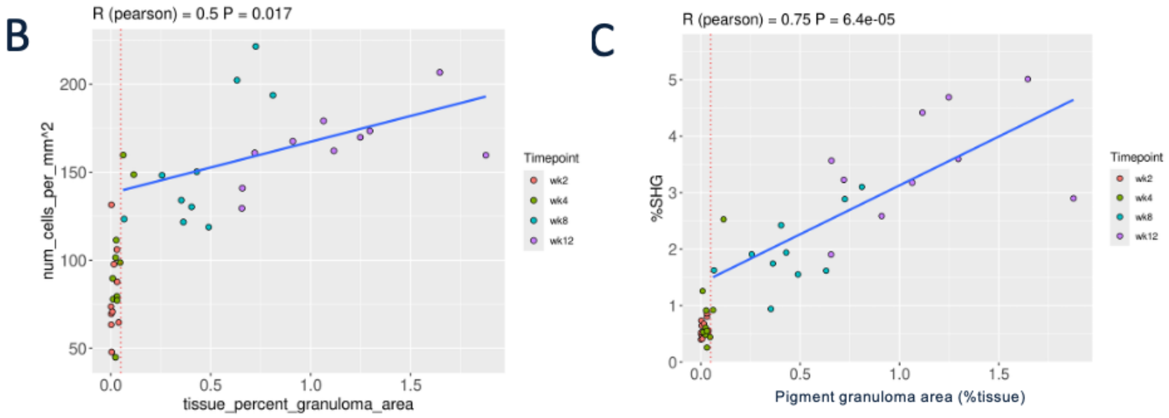

Figure S6. (B) Correlation between AI-derived pigment granuloma area and inflammation, (C) Correlation between AI-derived pigment granuloma area and fibrosis. Data are from CDAHFD-TA young; N=40, correlation was performed for data points with pigmented granuloma area larger than 0.05% tissue (generally data point after week 4).

5. In Figure 7, all three compounds increased fibrosis that was not co-localized with steatosis. How do the authors biologically interpret this finding?

Answer:

We thank the reviewer for this important point and agree that an increase in fibrosis, even when non-colocalized with steatosis, could be concerning if interpreted without context. To avoid misinterpretation, we would like to clarify the following methodological points and supporting data.

Definition and rationale for the colocalized / non-colocalized analysis

The non-colocalized fibrosis data in Fig. 7H,P,X are derived by partitioning the total fibrosis signal shown in Fig. 7D,L,T respectively into two spatial components: (a) fibrosis colocalized with steatosis (i.e., fibrosis in the immediate proximity of steatotic regions), and (b) the remaining fibrosis signal in tissue away from steatotic regions, which we refer to as "non-colocalized fibrosis." This partitioning was performed on the same whole-slide images using our AI-DP pipeline to enable spatially resolution of treatment effects.

The rationale for this "dupont" (spatial partition) analysis is to increase sensitivity for detecting treatment effects that are spatially specific. Colocalized fibrosis is a clinically relevant metric (used in several recent translational studies) because fibrosis adjacent to steatotic hepatocytes is most likely to reflect ongoing dysmetabolism/MASH-driven fibrogenesis; detecting improvement there can indicate efficacy even when global fibrosis measures are less sensitive.

Interpretation of the apparent increase in non-colocalized fibrosis

The apparent relative increase in the non-colocalized compartment is a residual – an artifact of partitioning rather than evidence of new or increased fibrogenesis induced by therapeutic interventions. In other words, because the same total fibrosis signal (Fig. 7D,L,T) is redistributed into two spatial bins, a decrease in steatosis-colocalized fibrosis can mathematically correspond to a relative increase in the remaining (non-colocalized) fraction even when the absolute total fibrosis is unchanged.

Importantly, total fibrosis did not differ significantly between treated and control groups; therefore the non-colocalized increase does not reflect a net increase in collagen burden.

Supporting biological and molecular evidence arguing against treatment-induced fibrosis worsening

Following this review, we profiled mRNA expression for fibrotic and inflammatory biomarkers (*Col1a1, Col3a1, Acta2, Timp1/2, CD68, IL1B, ADGRE1, TNF, Ccl2, Ccr2, Ccr5*), liver histopathology for additional fibrotic markers (*ASMA, Galectin-3, Col1a1*), and plasma chemistry (ALT, AST, Chol) for mice treated with Semaglutide (Supplementary Figure S9). We included a panel of pro- and anti-inflammatory cytokines via MSD (KC/GRO, IL6, TNFa, IP-10, MCP-1, IL-5, IL-10) and pro-fibrogenic mRNA profiling (*Col1a1, Col4a2, Acta2, Lgals1, TGB2, Saa1, Ccl2*) in resmetirom-treated animals (Supplementary Figure S8B-C). And we performed mRNA profiling for fibrotic and inflammatory markers (*Col1a1, Col3a1, Vim,*

TGFB2, TIMP1, TIMP2, IL1B, CD68, Ccl2) in ACCi treated mice (Supplementary Figures S9B). These results have now been added as a new panel in Supplementary Figure S8 and S9 accordingly.

In line with our explanation above, there was no biomarker signature indicative of active fibrogenesis, and no cytokine profile suggested elevated fibrogenic activity. Moreover, mRNA expression analyses showed no upregulation in treated animals; instead, expression levels trended toward improvement, consistent with reduced disease activity.

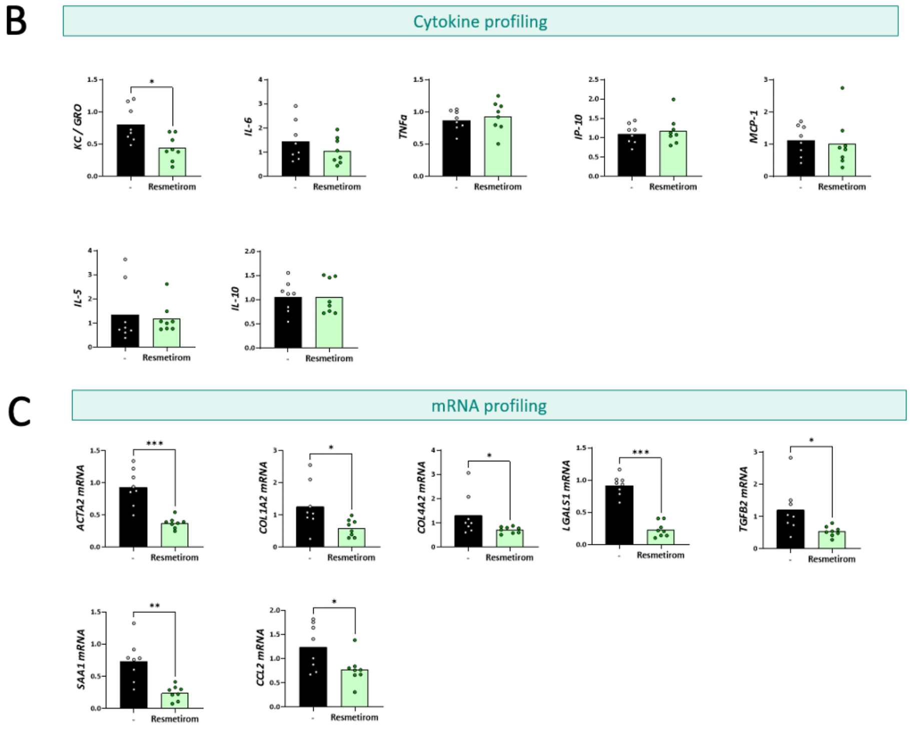

Supplementary Figure S8. (B) Cytokine profiling and (C) mRNA profiling of liver of the mice treated with Resmetirom.

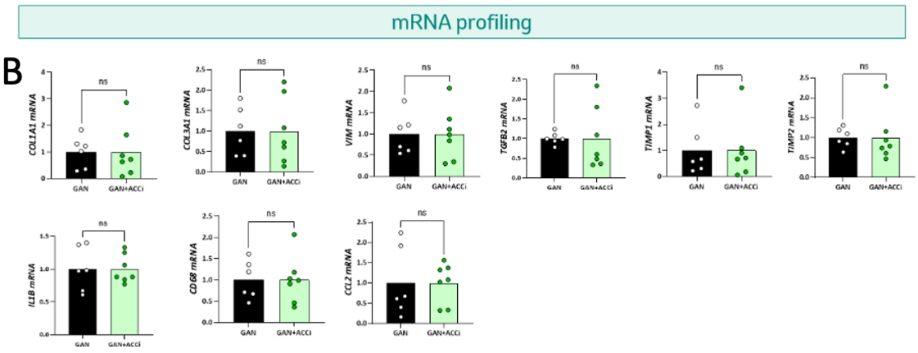

Supplementary Figure S9. (B) mRNA profiling of liver of the mice treated with ACCi.

We agree with the reviewer that the original wording risked overstatement. We therefore propose replacing the sentence that suggested the non-colocalized fibrosis increase "may reflect tissue remodeling as part of resmetirom's efficacy" with a more cautious text to avoid speculative claims about "remodeling" and clarify these points for the readers. The following changes were made to **Results section**, along with the additional panels in **Supplementary Figure S8-S9**, to support that there is no evidence of increased inflammatory or fibrotic activity in the treated animals:

"While there was no significant impact on total fibrosis (P=0.221; Fig. 7L), partitioning total fibrosis into regions colocalized with steatosis and non-colocalized regions reveals that resmetirom predominantly reduced fibrosis adjacent to steatotic areas by 2.4-fold (P=0.036; Fig. 7M), for both macrosteatosis and microsteatosis (Fig. 7N-O). The apparent increase in the non-colocalized fraction (P=0.003; Fig. 7P) reflects the residual – an artifact of partitioning distribution within the unchanged total fibrosis signal rather than an absolute rise in collagen burden. This interpretation is supported by our cytokine profiling and mRNA analyses, which show no evidence of increased inflammatory or fibrotic activity in treated animals (Fig. S8)."

"Similarly, the apparent relative increase in the non-colocalized compartment with ACCi treatment (Fig. 7X) reflects a partitioning artifact rather than true induction of fibrogenesis, a conclusion supported by mRNA profiling of fibrogenic and inflammatory markers (Fig. S9). While the use of a different mouse strain (C57BL6/N from Taconic) could affect model progression and drug responsiveness, based on the data presented, 10-weeks on the GAN diet is premature for robust fibrosis development. So, the apparent increase in fibrosis in PS (Fig. 7T) likely reflects an early and suboptimal timepoint for fibrosis assessment. Mechanistically, ACC inhibition may be more relevant to earlier stages of disease. In addition, prolonged ACCi treatment may have deleterious effects, including plasma hypertriglyceridemia[10] or elevated PNPLA3 mRNA[11]. For these reasons, the present study was designed to evaluate the impact of ACCi on steatosis, which was robustly captured using AI-driven DP."

6. Is the novel digital pathology pipeline applicable to human MASH specimens? Data on human samples would support that this technology might be useful for anti-MASH clinical trials.

Answer:

Our AI-DP pipeline was designed to detect various histopathological features of interests in MASH specimens such as the presence of lipid-droplets, inflammatory cells, fibrosis, as well as liver zonation based on central vein and portal vein landmarks, in mouse models. Our pipeline is applicable to human tissue; however, finetuning is needed to ensure optimal accuracy in these samples.

The two main modalities in our pipeline are SHG/TPEF image and H&E-stained image. The SHG/TPEF approach for MASH has been reported in multiple human studies including clinical trials (Matsuzaki et al.[12] and Akbary et al.[13] among many others). The same pipeline as implemented here by HistoIndex technology has been applied to human samples; therefore,

the application on human samples is relatively straight-forward. Our H&E-based approaches included the detection of lipid-droplets and inflammatory cells. Automated lipid-droplet detection has been explored in human samples for liver [14-17] and similar approach for inflammatory cell detection has been explored in human cancer samples [18].

The following paragraph has been added to Discussion:

"In our workflow, we combined HistoIndex SHG-based AI for sensitive fibrosis detection with an in-house H&E-based AI pipeline for features better resolved on H&E (e.g., inflammation, granulomas, and liver zonation). We then registered SHG and H&E modalities to enable zone-specific and colocalization analyses. Although optimized for mouse models, our approach is translatable to human-specimens or other experimental-models, with appropriate finetuning to account for species-specific histopathologic differences."

7. Many AI-based digital pathology methods have been described previously. What is the specific novelty of this approach, and how does it improve upon existing technologies?

Answer:

We thank the reviewer for raising this important query. There are currently no validated off-the-shelf or widely adopted digital pathology algorithms for comprehensive quantification of steatosis, inflammation, and fibrosis in murine MASH models, particularly those capable of capturing both disease progression and regression. The specific novelty of the approach we proposed in this manuscript is the integration of multi-modal AI-DP approach, enabling the colocalization between different histopathological features in MASH, such as steatosis/fibrosis colocalization, inflammation/fibrosis colocalization, or lipogranuloma/fibrosis colocalization, as illustrated in Fig 1. This approach provides a window to investigate disease mechanisms in MASH from relatively standard or accessible histology techniques. The colocalization analysis provides insights whether disease progression – as manifested in fibrosis progression – could be attributed to steatosis (i.e., metabolic-dependent pathway) or inflammation (i.e., pathways secondary to metabolism), as measured by fibrosis colocalizing with steatosis or inflammation, respectively. To our knowledge, such capability is not available in current digital pathology vendor platforms, with the exception of steatosis/fibrosis colocalization in the HistoIndex system, which is restricted to a single modality (SHG/TPEF). Our method therefore delivers a unique and rigorously novel capability beyond existing solutions.

In light of the limitations mentioned above, we benchmarked our AI-based pipeline against the current standards used in digital pathology practice, namely:

1. Benchmarking against expert hepatopathologist annotations and scoring, which is widely accepted as the "ground truth". Our AI model showed high correlation with pathologist scores for steatosis (Spearman's R=0.80, Fig. S10I), inflammation (Spearman's R=0.84, Fig. S4B), and fibrosis (Spearman's R=0.72, Fig. S10H).
2. Benchmarking against the established histochemical and immunostaining–based quantification approaches, as the current standard in histopathology.
    a. For fibrosis, we have also benchmarked the AI-derived fibrosis metrics against Picrosirius Red (PSR) staining–based quantification, a widely accepted

histological reference standard and demonstrated a strong corelation (Pearson's R = 0.92, Fig. S10G) supporting the performance of our model.

b. For inflammation, we performed additional experiments to further validate the AI model trained on H&E images using multiplexed immunofluorescence, including T-cell markers (CD3, CD4, CD8, FoxP3) and macrophage markers (CD68, CD44, F4/80, CD11b). This orthogonal validation demonstrated a strong correlation between AI-detected inflammatory cells and multiplexed-IF-defined immune populations (Pearson's R = 0.82, Fig S4A), supporting the biological fidelity of the model (see response to Reviewer 1, Q3). To further address reviewer concerns regarding model performance, we focused the AI model for the 40x magnification and re-prepared and scanned the slides that were previously scanned at 20x and 40x. To address the comments regarding interpretability and pathological relevance, we refined our inflammation readouts by introducing an inflammatory foci–based metric, aligning AI outputs with NASH-CRN criteria routinely used by pathologists. This refinement resolved previously noted discrepancies, such as elevated inflammatory cell counts in chow-fed animals, and improved concordance with pathological assessment (see response to Reviewer 1, Q3).

3. Clinically applied digital pathology technology to enhance translational relevance.
   a. We leveraged a clinically applied digital pathology platform (HistoIndex) to bridge preclinical findings with established clinical DP workflows. This approach provides a meaningful translational benchmark in the absence of murine-specific off-the-shelf algorithms.

Taken together, while direct head-to-head comparison with off-the-shelf murine DP algorithms is not feasible due to their absence, the revised manuscript provides robust and pragmatic benchmarking against the current state of digital pathology practice, integrating expert pathology assessment, established staining-based quantification, and clinically applied DP technology. Importantly, our incorporation of cross-modal colocalization constitutes a substantive methodological advance, enabling more comprehensive and interpretable quantification of steatosis, inflammation, and fibrosis in murine MASH. We believe this framework underscores both the strength and translational potential of the customized AI solution.

We added this point in the Discussion section:

"The specific novelty of our approach is the integration of the multi-modal AI-DP, enabling colocalization analysis between different histopathological features in MASH, such as steatosis (macro and micro)-fibrosis, inflammation-fibrosis, or lipogranuloma-fibrosis colocalization. This enables detailed assessment of inflammation and dysmetabolism in liver fibrosis progression across different zones, thereby deepening our understanding of the disease's etiology. "

8. Please check the manuscript carefully for other typographical errors. In Figure S9, the phrase "GLP1 agonism improve" should be corrected to "GLP1 agonism improves", for instance.

Answer: We thank the reviewer for noticing this. We have corrected the grammar in Figure S9, and have checked the manuscript carefully for other typographical errors.

Reviewer #2 (Remarks to the Author):

The manuscript presents a comprehensive study on the application of AI-driven digital pathology to analyze murine models of Metabolic Dysfunction-Associated Steatohepatitis (MASH) and liver fibrosis. The authors have developed and validated a multi-modal AI pipeline for zone-specific analysis and spatial co-localization of key MASH features, providing insights beyond standard histology. The study is well-conducted, and the results are promising, highlighting the potential of AI in improving the understanding and management of MASH and fibrosis. My concerns for this manuscript are as follows:

1. The authors stated that lipid-droplets <15 µm were classified as microsteatosis and ≥15 µm as macrosteatosis. This critical threshold lacks biological justification. Why 15 µm? Was this threshold validated against established histological criteria or biochemical measures of lipid-droplet toxicity?

Answer:

[19]The critical threshold of 15 µm for the classification of macrosteatosis and microsteatosis was based on what was reported in literature [20], as well as empirical and data-driven observations. The pre-Banff method, used by Transplant Pathology Unit of the AOU Città Della Salute e Della Scienza di Torino Hospital, used an area of 200 µm^2 to stratify large droplet steatosis from small droplet steatosis. This is approximately ~16 µm in diameter, assuming the lipid-droplet is circular [21]. In reality, the lipid-droplet might not be exactly circular. In our study, we used a circularity factor of 0.8 [22] to define lipid-droplets. Given the area of an ellipse is πab, where a is the half the length of the major axis and b is the half the length of the minor axis, an ellipse of a = 8.5 µm (equal to full axis length of 17 µm) and b = 7.5 µm (equal to full axis length of 15 µm) would mean an area of about 200 µm^2.

We can observe the critical threshold by visualising several examples of lipid-droplets at different sizes. Supplementary Figure S12A (in red) shows randomly selected lipid-droplets at ~5 µm (i.e. between 4.5 – 5.5 µm),~10 µm, ~14 µm, ~16 µm, ~20 µm, and ~30 µm, across one of the models, CDAHFD from a sample WSI. Detected lipid droplets ≤10 µm are smaller than half a cell[23]. Droplets ≥16 µm exceed half the cell size and are unequivocally macrosteatosis. Although 14 µm is visually ambiguous, the threshold lies between 14 and 16 µm, centering near 15 µm. Consistent with this, the distribution in Supplementary Figure S12B shows two dominant clusters—microsteatosis and macrosteatosis—separated at approximately 15 µm in diameter.

We added the following details in **Materials & Methods Section "Lipid-droplet quantification"** and added **Supplementary Figure S12**;

“Individual lipid-droplet on the H&E images was detected using Vacuole 2.2 algorithm within HALO software (Indica Labs). The algorithm was fine-tuned separately for lipid-droplets with diameter smaller than 15 µm (classified as microsteatosis) and larger or equal to 15 µm (classified as macrosteatosis), with a circularity factor of 0.8. The 15 µm threshold was determined based on previous report [20], as well as empirical and data-driven observations (Supplementary Figure S12).”

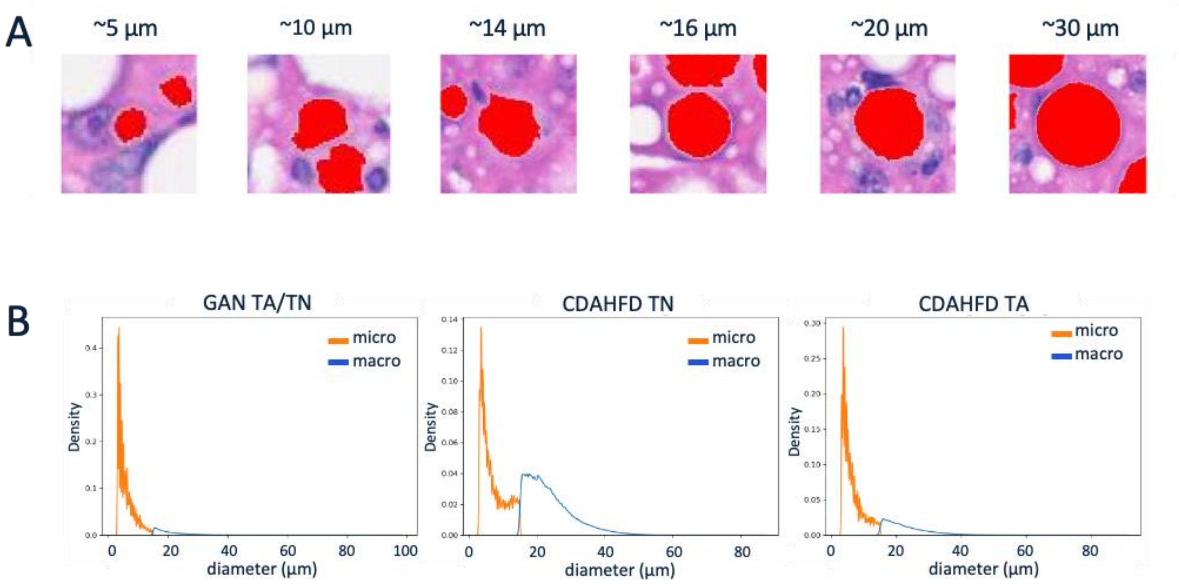

Supplementary Figure S12. (A) Randomly selected lipid-droplets with a diameter range of 5-30 µm from a sample image, (B) distributions of microsteatosis and macrosteatosis across the different models.

2. The authors emphasized the AI's ability to distinguish micro/macrosteatosis but did not robustly link this distinction mechanistically to disease progression or treatment response beyond quantification. Does microsteatosis correlate more strongly with specific metabolic pathways or inflammatory signals than macrosteatosis in the omics data? The relevance of this AI-derived distinction for MASH pathophysiology or drug efficacy needs stronger justification.

Answer: In our dataset the AI-derived distinction between micro- and macrosteatosis is supported by biologically plausible metabolic signals, but important limitations prevent definitive mechanistic claims. De novo lipogenesis (DNL) favors production of saturated fatty acids and a relative loss of polyunsaturated fatty acids (PUFAs), and our lipidomic and metabolomic data are consistent with this pattern at weeks 12 and 23 in GAN-fed mice. (Supplementary Fig. S1D,F). Microsteatosis at TN was more advanced in GAN fed mice than at TA while macro-steatosis was comparable at the two housing temperatures at the week 12 timepoint (Fig. 2B,C); this spatial pattern parallels stronger trends toward fatty-acid saturation and shorter chain lengths at TN relative to TA as gauged by lipidomics (Fig. S1D) These observations support the view that regions with prominent microsteatosis could show biochemical signatures consistent with enhanced DNL, altered fatty-acid remodeling, and

metabolic features implicated in lipotoxicity and progression of steatohepatitis. However, our ability to link micro- versus macrosteatosis mechanistically to specific lipidomic, metabolomic or transcriptomic signatures is limited. The omics assays were performed on whole liver (or plasma) at a small number of time points and were not spatially co-registered with the histologic AI segmentation. Bulk measurements average signals across heterogeneous regions and cell types, obscuring regional heterogeneity and preventing unambiguous assignment of particular transcripts, lipids or metabolites to micro- or macrosteatotic foci. We also did not perform omics in the drug efficacy cohorts, so we cannot evaluate whether treatments differentially modulate micro- versus macrosteatosis at the molecular level. In this study omics served primarily as a model-validation step at a few checkpoints and reproduced many previously reported biomarker changes, thereby confirming the murine model.

To address the reviewer's concern that the relevance of the AI-derived distinction of macrosteatosis and microsteatosis as drivers of disease progression needs stronger justification, we additionally performed colocalization analysis between micro-/macro-steatosis (instead of just total steatosis) and liver fibrosis. In GAN model, magnitude of colocalization with liver fibrosis was higher with macrosteatosis with response being accelerated and exacerbated at TN relative to TA housing conditions (added as new panels: Figure 5H,I,M,N). The magnitude of colocalization with macro-steatosis was amplified in the CDAHFD model both young and aged (added as new panels: Figure 5J-L,O-Q). In a previous study [24], qFibrosis co-localized with macrosteatosis generally correlated better with histological scoring and had a higher AUC in six animal models. Further, this AI segmentation and colocalization allowed us to resolve how different therapeutics affect micro- versus macrosteatosis and their colocalization with fibrosis histologically: semaglutide primarily reduced macrosteatosis colocalized fibrosis; resmetirom reduced both macro- and microsteatosis colocalized fibrosis; ACC inhibitor (ACCi) had little effect on either (added as new panels: Figure 7F-G,N-O,V-W). These patterns suggest that the micro/macro distinction may be relevant for interpreting drug efficacy, but they are histologic observations not yet linked to spatial molecular mechanisms.

Our data provide biologically plausible support for the AI distinction: regions identified as microsteatosis capture lipidomic/metabolomic trends consistent with DNL and fatty-acid remodeling, and histologic colocalization analysis links macrosteatosis more strongly to fibrosis. Together these findings argue that the AI-derived micro/macro classification captures pathologically relevant heterogeneity.

To establish stronger causal and therapeutic links will require spatially resolved molecular profiling co-registered to AI segmentation (for example, laser capture microdissection followed by lipidomics/transcriptomics, or spatial transcriptomics/proteomics/metabolomics) and longitudinal omics in treatment studies to determine whether therapies differentially reverse micro- versus macrosteatosis and their associated inflammatory or fibrogenic pathways.

We added the following paragraph in the Results:

Sub-section "AI-DP reveals temporal and spatial localization of steatosis development in different diet induced models of MASH and liver fibrosis"

"De novo lipogenesis (DNL) favors production of saturated fatty acids with a relative loss of polyunsaturated fatty acids (PUFAs), consistent with our lipidomic and metabolomic data (Fig.

S1D, F). In GAN-fed mice at weeks 12 and 23, microsteatosis is more advanced at TN than TA (Fig. 2B,C) paralleling stronger fatty-acid saturation and shorter chain lengths at TN in lipidomics (Fig. S1D) and downregulation of long-chain PUFAs in metabolomics (Fig. S1F). These patterns suggest that regions with prominent microsteatosis show biochemical signatures of enhanced DNL, altered fatty-acid remodeling, and metabolic features linked to lipotoxicity and steatohepatitis progression. Such mechanistic claims can be confirmed via spatial co-registration of molecular data with histologic AI segmentation."

Sub-section "Spatial colocalization of steatosis, inflammation, and fibrosis by AI-DP reveals mechanistic drivers of disease progression in murine models" and added panels to Figure 5:

"The colocalization analysis of fibrosis adjacent to microsteatosis (Fig. 5H-L) or macrosteatosis (Fig. 5M-Q) demonstrates the critical importance of differentiating macrosteatosis and microsteatosis. In GAN model, time-dependent increase of the fibrosis colocalization with microsteatosis and macrosteatosis was accelerated and exacerbated at TN than at TA with the magnitude of colocalization with liver fibrosis being higher with macrosteatosis than with microsteatosis (Fig. 5H-I,M-N). In CDAHFD, temporal severity of disease manifestation remained similar between TA and TN perhaps owing to aggressive nature of the disease model, with more prominent time-dependent increase in fibrosis colocalized with macrosteatosis (Fig. 5J-K,O-P) suggesting macrosteatosis, rather than microsteatosis, being the driver of fibrosis in this model as well. In the more severe model of CDAHFD, aged CDAHFD at TA, there was a more intense time-dependent increase in microsteatosis and macro-steatosis colocalized fibrosis at week 8 (when compared with other models) with fibrosis colocalizing with macrosteatosis being consistently higher than that with microsteatosis (Fig. 5L, Q). These findings are in line with a previous study [24] that showed qFibrosis co-localized with macrosteatosis generally correlated better with histological scoring and had a higher AUC in six animal models."

Sub-section "Validation of AI-DP using clinically validated assets":

"AI-DP showed a notable reduction in macrosteatosis (1.7- to 2-fold lower vs. vehicle, P<0.001; Fig. 7A) with Semaglutide while no significant reduction in Fibrosis across different zones with the treatment (Fig. 7D). Partitioning total fibrosis into regions colocalized with steatosis and non-colocalized regions showed semaglutide reduced steatosis-colocalized fibrosis (1.7-fold P=0.05, Fig. 7E), which is mostly attributed to the reduction the colocalization with macrosteatosis rather than microsteatosis (Fig. 7F-G). "

"While there was no significant impact on total fibrosis (P=0.221; Fig. 7L), partitioning total fibrosis into regions colocalized with steatosis and non-colocalized regions reveals that resmetirom predominantly reduced fibrosis adjacent to steatotic areas by 2.4-fold (P=0.036; Fig. 7M), for both macrosteatosis and microsteatosis (Fig. 7N-O)."

"ACCi did not show efficacy in reducing fibrosis, steatosis colocalized fibrosis or micro-/macro-steatosis colocalized fibrosis (Fig. 7T,U,V,W)."

We also added the following paragraph in the ***"Discussion"*** :

"Our data provides biologically coherent AI-derived micro/macro classifications: microsteatotic regions aligned with lipidomic/metabolomic signatures of de novo lipogenesis and fatty-acid remodeling, whereas macrosteatosis colocalized more strongly with fibrosis, indicating pathologically relevant heterogeneity."

"To strengthen causal inference, therapeutic relevance, and prognostic utility, future work should integrate AI-based segmentation with spatial omics or laser-capture microdissection, and conduct longitudinal intervention studies to define the role of granulomas in tissue

remodeling and disease etiology, and determine whether interventions differentially reverse micro- versus macrosteatosis and their associated inflammatory and fibrogenic pathways."

Addition to Figure 5:

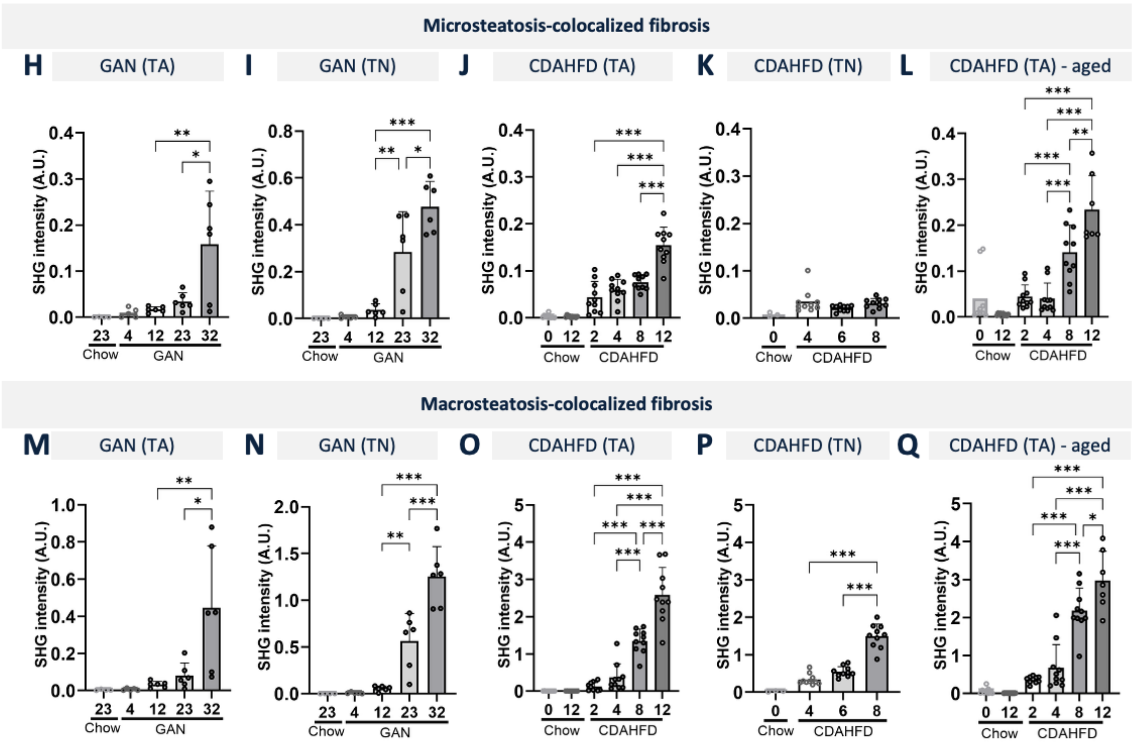

Figure 5. (H-L) Quantification of fibrosis colocalizing with microsteatosis and (M-Q) macrosteatosis in different animal models.

Addition to Figure 7:

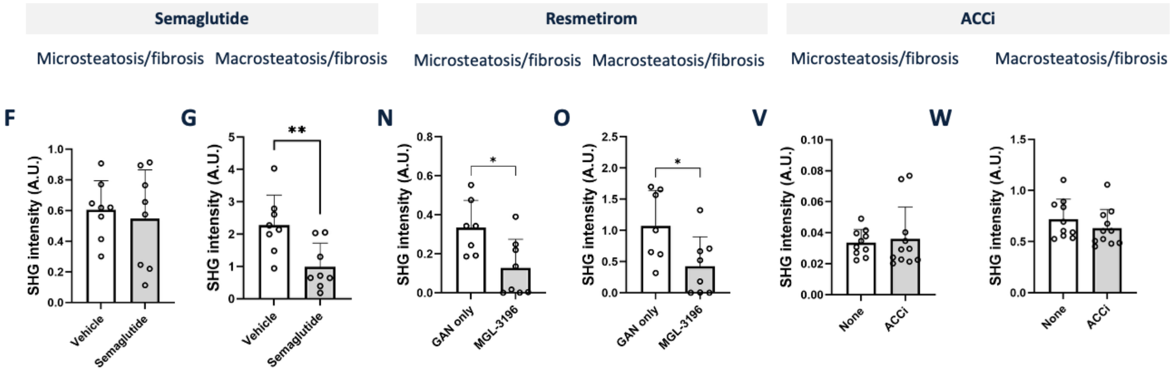

Figure 7. Impact of (F-G) semaglutide, (N-O) resmetirom, and (V-W) ACCi on (F, N, V) microsteatosis-colocalized fibrosis and (G, O, W) macrosteatosis colocalized fibrosis.

3. The CDAHFD model is largely induced by choline deficiency, which impairs VLDL secretion and leads to steatosis, along with high fat. It's more of a rapid toxin/diet-induced model for steatohepatitis and fibrosis, unlike the GAN diet that better mimics human overnutrition. The current description might oversimplify the complex, partially toxic nature of CDAHFD's etiology, which could weaken the analysis of model differences and the colocalization analysis. It would be better to stress the distinct mechanisms of CDAHFD in the Introduction.

Answer:

Thank you for raising this concern. We have included the. Following passage in the **Introduction**.

GAN better models chronic metabolic disease processes relevant to human overnutrition, while CDAHFD is a rapid, fibrosis-focused model useful for studying mechanisms of injury and fibrogenesis. The GAN diet (high fat, fructose, and cholesterol) induces steatosis via metabolic overload characterized by lipid accumulation, oxidative stress[25], and immune activation leading to hepatocyte ballooning, chronic inflammation, stellate-cell activation, and gradual fibrogenesis, mirroring dysmetabolism-driven human MASH. In contrast, CDAHFD acutely impairs lipid export, phospholipid/membrane synthesis and mitochondrial function, triggering oxidative stress, permeability transition, rapid hepatocellular injury, and brisk fibrosis within weeks [26-28]. Operating via toxin-like mechanisms and lacking systemic features (weight gain, insulin resistance), CDAHFD only partially captures the metabolic milieu of human MASH. "

4. The inflammation detection model performs significantly worse on the GAN model (ROC AUC/PR AUC 0.82/0.55 at 20x) compared to CDAHFD (0.92/0.93 at 40x). Was the GAN model trained on lower-quality images or fewer annotations? Can the pipeline be reliably used across models if performance varies this drastically? This limitation needs critical discussion.

Answer:

Thank you for raising a valid query regarding the performance gap between the 20× GAN model (ROC AUC/PR AUC 0.82/0.55) and the 40× model used in CDAHFD (0.92/0.93). Indeed, we had two AI models for the inflammatory cell detection – one was trained for 20x WSI and the other one for 40x WSI. The 20x model was trained specifically for the GAN samples (in Figure 3), as these datasets were originally only available at 20x. However, the 40x model was trained across different models (not only CDAHFD). The interventional studies (Figure 7) were conducted on GAN, and the 40x model was applied for those samples.

The primary limitations of the images scanned at 20x were annotation volume and confidence. The 20× model was trained on fewer annotations (~1,000 cells) than the 40× model (~4,000 cells). In addition, annotation quality at 20× was inferior: pathologists were less able to reliably identify cells, leading to a higher proportion labelled as "uncertain." These factors likely contributed to the lower performance of the 20× model relative to the 40× model. With

harmonized 40× scanning and adequately curated annotations, the pipeline demonstrates reliable performance across models.

We have added explicit discussion of this limitation and additional details on model tuning in our response to Reviewer 1, Q3.

5. The study identifies granulomas and lipogranulomas as morphometric features of MASH. However, the significance of these structures in disease progression is not clearly established. Are these structures indicators of disease severity or specific stages of MASH progression? How do they correlate with other phenotypic metrics such as fibrosis and inflammation?

Answer: Mechanistically, both lesion types, lipogranuloma and pigmented granuloma reflect overwhelmed lipid handling when hepatocellular lipid release exceeds the clearance capacity of resident phagocytes, causing excess lipid to be sequestered by macrophages. Pigmented granulomas represent a similar process with additional accumulation of autofluorescent pigments (ceroid/lipofuscin) and often iron deposition, consistent with ongoing oxidative stress and phagocytic processing. Immunophenotyping in our samples indicates these aggregates are macrophage-rich (CD44+, CD68+) consistent with literature[6,8], supporting their identity as sites of active lipid uptake and macrophage activation. We have performed additional experiments using multiplexed-IF consisting of 4-plex macrophage markers (CD44, CD68, CD11b, and F4/80) and demonstrated that CD44+ cells are the major infiltrates, occurring at 10x higher levels compared to the CD68+ cells as the second highest infiltrates. See also the response to Reviewer 1 Question 4.

Regarding disease stage and severity, granulomas should be interpreted as indicators of local lipid trafficking dysfunction and sustained macrophage activation rather than as pathognomonic stage markers. Such lesions are associated with chronic inflammation and, in the literature and experimental models, have been linked to increased risk of progression to hepatocellular carcinoma (HCC). Following the reviewer's question, we looked into the correlation between pigmented granuloma area and inflammation, and we observed that pigmented granuloma started to increase and correlate with inflammation after the inflammation reaches a certain level (in our dataset: after week 4 in CDAHFD), supportive of chronic inflammation (added as a panel B in Supplementary Fig. S6). Notably, the most salient quantitative association was between pigmented granuloma prevalence and fibrosis: pigmented granulomas correlated strongly with higher fibrosis in the CDAHFD model (added as panel C in Supplementary Fig. S6, in addition to the colocalization analysis in Fig 6J-M), supporting a relationship between pigment-laden macrophage aggregates and fibrogenic progression in this rapid, toxin-like diet model. We observed less clear co-variation between lipogranuloma counts and fibrosis in the GAN model, which develops fibrosis more slowly; in that setting lipogranulomas appear more closely tied to local steatotic burden and macrophage lipid handling.the spatial and temporal heterogeneity of granulomas means they are not a standalone staging metric. Their presence and prognostic significance depend on context — diet, duration of injury, coexisting iron or oxidant stress, and fibrogenic response — and granulomas can regress if the underlying insult is removed. Because our omics were performed on bulk liver, we cannot yet ascribe distinct molecular signatures uniquely to

granuloma-containing regions; spatially resolved molecular profiling would be needed to establish causal links between granuloma formation, inflammatory signaling pathways and fibrogenesis. We therefore propose that granulomas and lipogranulomas are meaningful morphometric readouts of disrupted lipid handling and macrophage activation in MASH and that pigmented granulomas, in particular, are associated with fibrotic progression in the CDAHFD model. To strengthen causal inference and evaluate prognostic utility, follow-up studies should combine AI segmentation with spatial transcriptomics/proteomics or laser capture microdissection and longitudinal intervention studies to determine whether changes in granuloma burden predict or mediate fibrosis and treatment response.

Following the reviewer's comment, we have added the following paragraph:

We added the biological explanation of pigmented granuloma and additional data in the Results subsection : **"AI-DP for evaluation of pigmented granulomas and lipogranulomas in CDAHFD and GAN models of MASH and liver fibrosis"**, **Figure 6, and Supplementary Figure S6**:

"We temporally evaluated pigmented granulomas using AI-DP (Fig. 6F-H), noting an increase in lesion area over time. In the CDAHFD model, factors such as age (2.1-fold higher in aged vs. young mice at week 8, P=0.015; Fig 6F and 6H) and housing conditions (3-fold higher at TN vs. TA at week 8 in age-matched young mice, P<0.001; Fig 6F-G) influenced granuloma growth. Pigment granuloma starts to correlate with inflammation after week 4 (Pearson's R=0.51, P=0.016, Supplementary Fig. S6B), supporting the association with chronic inflammation [7]. We observed an increasing time-dependent prevalence of CD44+ cells in the pigmented granuloma, which was about 8-fold at week 8 compared to CD68+ cells as the second highest population within the pigmented granuloma (Fig. 6E, I). This supports previous reports that granulomas reflect ongoing lipid handling/stress and macrophage activation (specifically CD44+ and CD68+) within the liver [6,8]. Notably, pigmented granuloma prevalence correlated strongly with liver fibrosis (Pearson's R=0.75, P<0.001, Supplementary Fig. S6C), which is supported by the colocalization analysis between pigmented granuloma and fibrosis (Fig. 6J-M)."

Addition to Figure 6:

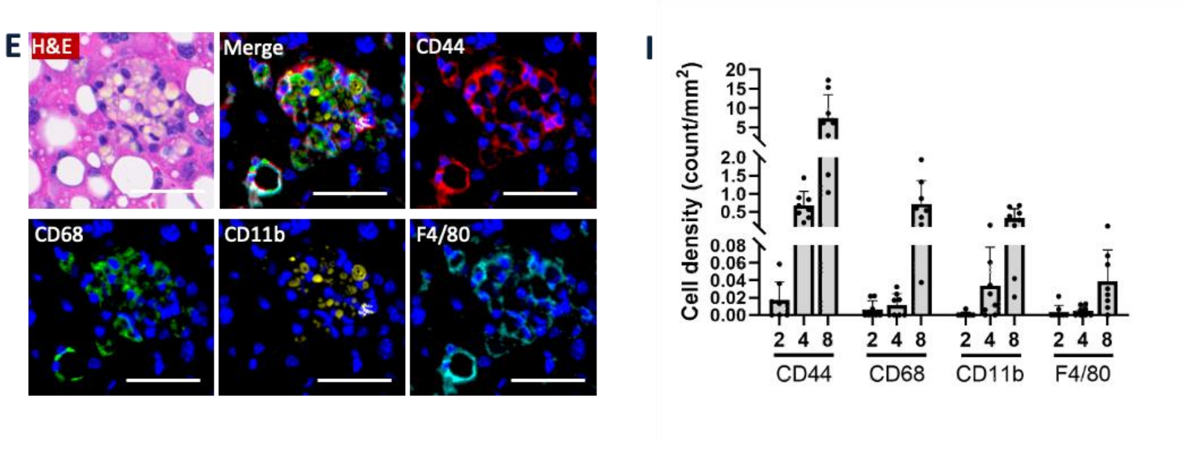

Figure 6. (E) Multiplexed-IF for macrophage panel (CD44,CD68, CD11b, F4/80), along with the H&E on the same section, further demonstrates the presence of macrophages in the

pigmented granuloma. (I) Contribution of the different macrophages to the pigmented granuloma.

Addition to Supplementary Figure S6:

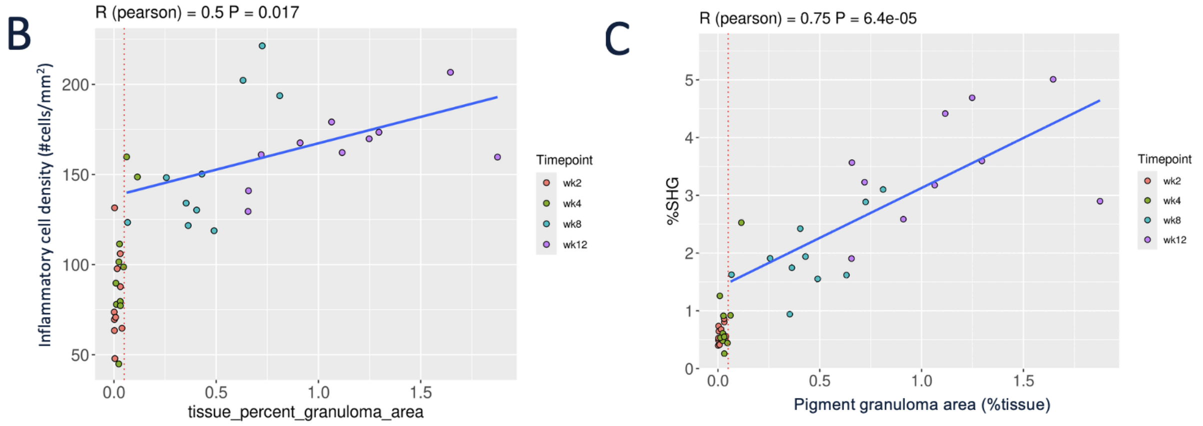

Figure S6. (B) Correlation between AI-derived pigment granuloma area and inflammation, (C) Correlation between AI-derived pigment granuloma area and fibrosis. Data are from CDAHFD-TA young; N=40, correlation was performed for data points with pigmented granuloma area larger than 0.05% tissue (generally data point after week 4).

Additionally, we also added the following paragraph in the Discussion section :

"These reflect lipid-trafficking dysfunction and macrophage activation (predominantly CD44+/CD68+), and their emergence correlated with fibrosis progression. Prior reports suggest lipogranulomas may promote liver cancer even without cirrhosis [26], but their prognostic significance is context-dependent—shaped by diet, injury duration, iron load or oxidative stress, and the fibrogenic response—and granulomas can regress if the insult is removed. Given their temporal heterogeneity, granulomas are unsuitable as standalone staging metrics"

"To strengthen causal inference, therapeutic relevance, and prognostic utility, future work should integrate AI-based segmentation with spatial omics or laser-capture microdissection, and conduct longitudinal intervention studies to define the role of granulomas in tissue remodeling and disease etiology, and determine whether interventions differentially reverse micro- versus macrosteatosis and their associated inflammatory and fibrogenic pathways."

6. The significant increase in non-colocalized fibrosis with Resmetirom treatment (Fig 7L, Lines 337-340) is interpreted as potentially reflecting "tissue remodelling as part of resmetirom's efficacy." This interpretation is highly speculative and potentially misleading. An increase in fibrosis, even if non-colocalized, is generally considered detrimental in MASH. Proposing it as part of efficacy requires robust supporting evidence not provided here (e.g., markers of collagen degradation/remodeling, functional improvement independent of histology, comparison to known remodeling phases). Without this, the data could equally indicate a lack of efficacy or even a pro-fibrotic effect under certain conditions. This finding warrants a more cautious and critical interpretation.

Answer:

Please refer to response to Reviewer # 1 Q.5 above.

7. The interpretation of microsteatosis plateauing while macrosteatosis increases due to "fusion" (Lines 217-218) is speculative. The data presented (size distribution) supports droplet growth but not fusion. While fusion is a potential mechanism, alternative explanations, such as preferential expansion of existing large droplets or differential susceptibility of hepatocyte zones, are not adequately considered.

Answer:

We thank reviewer for pointing this out. Indeed, the size distribution suggests the growth in lipid-droplet, and indeed there are a few potential explanations. The increase in lipid-droplets may be explained by coalescence/fusion of lipid-droplets or diffusion of neutral lipids from one lipid-droplet to another [29] or expansion as a result of lipid synthesis occurring directly on the surface of lipid-droplets or at newly formed lipid-droplets-ER membrane bridges [30]. Indeed, there may be differential susceptibility of the different zone to the mechanisms of the lipid-droplet growth ((i.e., fusion or expansion) as the metabolic gradient for TG synthesis is highest in CV region; Further experiments will need to be designed to address this and it is beyond the scope of this manuscript.

We edited the Results subsection: **“AI-DP reveals temporal and spatial localization of steatosis development in different diet induced models of MASH and liver fibrosis”**

“This growth in lipid-droplets may be explained by droplet coalescence/fusion, diffusion of neutral lipid between lipid-droplets[29] , or direct lipid synthesis on droplet surface[30]. These mechanisms of lipid growth may show differential zonal susceptibility along liver’s metabolic gradient”

8. The interpretation of steatosis progression following a "biochemical pathway" gradient from Zone 3 to Zone 1 and its implied similarity to human liver zonation requires caution. While human liver has metabolic zonation, the specific patterns and drivers in murine models under these diets may not perfectly recapitulate the human situation. The discussion overstates the direct clinical relevance of the murine zonal patterns observed.

Answer:

In human, despite the metabolic zonation with the gradient for TG synthesis being higher in pericentral and lower in periportal[31], the predominant accumulation of fat in hepatocytes can be categorized in four distributions: panacinar, zone 1 (periportal), zone 3 (centrilobular or pericentral), and azonal[32] – see Figure R2 below.

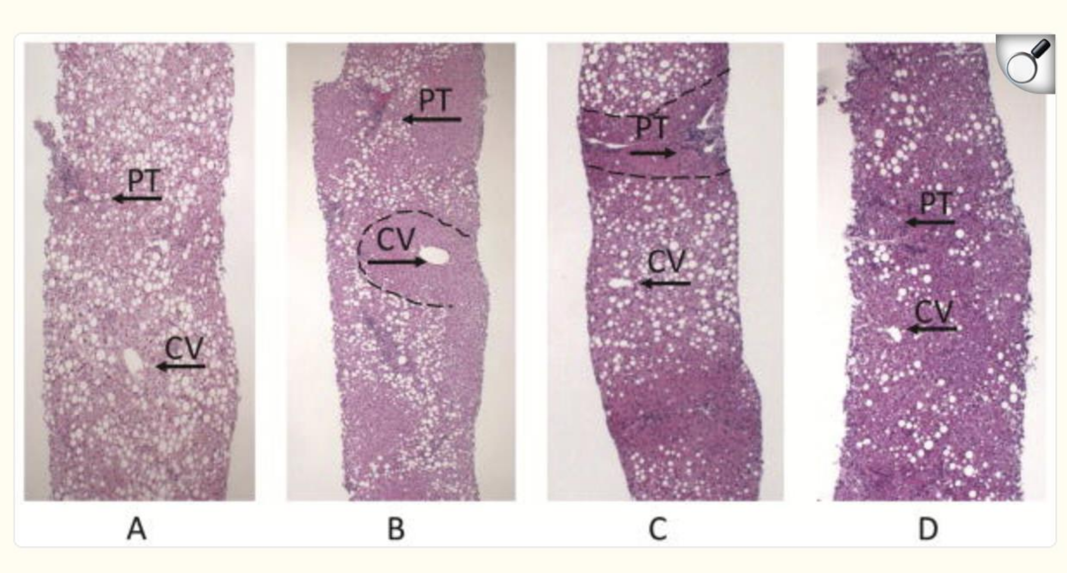

Figure R2. Pattern of steatosis distribution in human liver: (A) panacinar, (B) zone 1/periportal, (C) zone 3/pericentral, (D) azonal [32]

The pattern of steatosis development in both GAN and CDAHFD appears to be panacinar in both GAN and CDAHFD; however, there is a clear distinction of the distribution of microsteatosis vs. macrosteatosis in GAN vs. CDAHFD. In GAN, there is a clear distinct pattern of predominant microsteatosis in pericentral and macrosteatosis in periportal region, the distribution that is maintained even as disease progresses at 32-week timepoint (Supplementary Figure S2A-B). In CDAHFD, lipid-droplets are predominantly macrosteatosis, and this was observed as early as 4 week timepoint (Supplementary Figure S3A-B).

We agree with the reviewer that the specific patterns and drivers in the mouse models under these diets may not perfectly recapitulate the human situation. The steatosis development in mouse models seems to be more well-controlled due to the more controlled dietary intake. However, we argue that in GAN, the development of steatosis seems to be following the metabolic zonation system known for TG synthesis [31]. In addition to the pattern of predominant microsteatosis in pericentral/macrosteatosis in periportal in GAN, the appearance of microsteatosis occurs at earlier timepoint compared to the macrosteatosis in periportal. However, this was not observed in CDAHFD, which may be explained by the deficiency of choline in the diet that most likely results in the deficiency of phosphocholine required to maintain surface tension of the lipid-droplets, leading to lipid-droplet fusion to form macrosteatosis [29], even at the early timepoints.

We edited the paragraph in Discussion as follow:

"AI-enabled DP clarified zonal patterns of steatosis. In human liver, triglyceride synthesis pathways are enriched pericentrally (Zone-3) and least periportally (Zone-1)[31]. Further, in human MASH, accumulation of fat in hepatocytes follows four distribution patterns: panacinar, zone-1 (periportal), zone-3 (centrilobular or pericentral), and azonal [32]. In both GAN and CDAHFD models, the distribution appears to be panacinar with distinct distribution of microsteatosis vs. macrosteatosis in GAN and CDAHFD. In GAN, microsteatosis emerged earlier and predominated pericentrally and macrosteatosis periportally, a pattern evident early and maintained through 32-weeks (Fig. S2), consistent with metabolic zonation [31]. In

CDAHFD, macrosteatosis dominated all zones as early as 4-weeks, likely due to choline deficiency reducing phosphocholine related lipid-droplet surface-tension leading to lipid-droplet coalescence to form macrosteatosis [29]. These zone-resolved differences show how model-specific drivers shape steatosis and may diverge from human disease."

Reviewer #3 (Remarks to the Author):

The submitted article „AI digital pathology as a key tool providing in-depth understanding of the progression and regression of MASH and fibrosis in mice" by Dr Talukdar and colleagues provides and summarizes a broad variety of data on MASH and liver fibrosis in mouse models for human steatotic liver disease. As this work analyses pathological, computational, and pharmaceutical data, it is very comprehensive and forward-focused.

Especially regarding the pathological aspects of the study, we would like to raise concerns mainly from a methodological point of view.

1. Were there any (liver-associated) background lesions in the examined mouse models detected and taken into account? Please state, how a reliable delineation of background lesions vs. experimentally induced pathologies was ensured (e.g., Fig. 3 inflammation vs extramedullary hematopoesis)

Answer:

We thank the reviewer for this important point. Background (spontaneous) liver lesions and their distinction from diet- or treatment-induced pathology were explicitly considered during histopathologic evaluation. All slides were reviewed by board-certified veterinary pathologists who compared findings across experimental groups and chow-fed controls; lesions present at similar frequency and distribution in controls and treated animals were classified as background.

Small foci of extramedullary hematopoiesis (EMH), typically limited in extent in chow controls, were considered background for this colony/strain. EMH was identified by its cell composition (erythroid precursors, megakaryocytes and variable myeloid precursors), architecture and location in sinusoids or portal tracts, and by the absence of associated hepatocellular necrosis or portal expansion. In contrast, inflammatory infiltrates showed a predominance of mononuclear cells (lymphocytes, macrophages) and/or neutrophils, different topography (diffuse or portal-centered), and were often accompanied by hepatocellular injury such as ballooning.

We acknowledge that myeloid-predominant foci can be difficult to distinguish from inflammation on H&E alone. In such ambiguous cases we used lesion context (occurrence in controls, co-existing injury patterns and distribution) and conservative attribution to avoid misclassification. Definitive lineage confirmation can be obtained by targeted IHC (e.g., Ter119, CD41, MPO).

Similarly, for the AI model detecting inflammatory cells, we provided image patches for pathologist review, and pathologists annotated individual cells. The revised manuscript now

reports the number of inflammatory foci (as opposed to individual inflammatory cells) as the quantitative metric, consistent with lobular inflammation in the NAS system. Because EMH typically forms larger clusters (see Figure R3), its contribution is expected to be minimal relative to the total clusters identified across the tissue.

Overall, pathologist review, comparison to controls, morphologic criteria and conservative classification provided a reliable delineation between background EMH and experimentally induced inflammation in these studies.

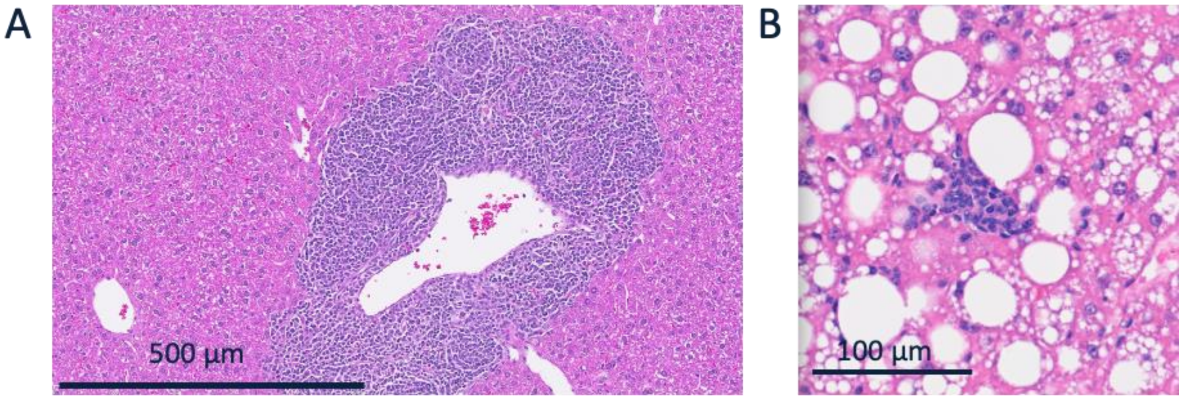

Figure R3. Examples of (A) EMH cluster in chow and,(B) a representative lobular inflammatory focus in GAN. Note that the cluster size differs.

2. With regard to feeding / 5 hour postprandial fasting period prior to experiment termination and mouse necropsy we would like to know if intra-hepatocyte glycogen storage was still present in the harvested liver tissues and how a reliable differentiation between cytoplasmic clearing due to glycogen vs. lipid storage was carried out.

Answer:

Thank you for this important question. 4-6 hours of fasting before termination allows standardization of metabolic readouts from blood while minimizing confounding effects of prolonged fasting. A short fast (4-6h) ensures mice are in post-absorptive, steady metabolic phase where lipids reflect hepatic lipid metabolism rather than reflecting post prandial variability in plasma triglycerides, blood glucose and insulin levels owing to nutrient absorption and hepatic chylomicron/VLDL handling.

Based on the histopathologist's review and annotations, intra-hepatocyte glycogen was unremarkable to only mild/moderate in the GAN and CDAHFD murine MASH models at the time of necropsy (5-hour postprandial fasting). Differentiation between glycogen and lipid (steatosis) was performed by routine light-microscopy assessment of H&E-stained sections and by established morphological criteria. Lipid appears as clear, round, non-membranous vacuoles that displace the nucleus (macrovesicular) or form multiple smaller droplets (microvesicular) with sharp, well-defined borders. Glycogen causes a more homogenous, pale cytoplasmic clearing that is often finely granular and does not produce the sharply

demarcated, spherical vacuoles typical of lipid. The morphological nuance has been captured in the image below for reference.

The following sentence was added to Materials & Methods

Subsection "Animals"

"After 5 hours of fasting, intra-hepatocyte glycogen was unremarkable to only mild/moderate level, minimizing ambiguity of cytoplasmic clearing due to glycogen vs. lipid-droplets."

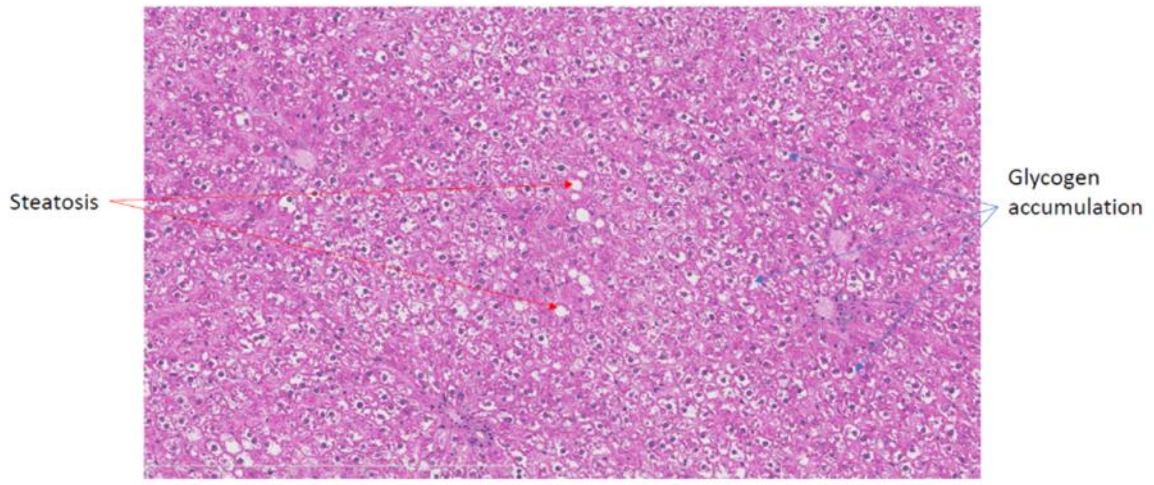

Figure R4. Example of glycogen accumulation (moderate) in tissue sample.

3. NASH-CRN scoring was developed as a histological scoring system for human liver biopsies. The methodology of NASH-CRN scores needs to be described in more detail incl. the individual scoring parameters. It needs to be explained how this human scoring system can be translated to the murine situation. According to our experience, comparative hepatocyte ballooning cannot be seen in mouse livers. Please provide a convincing histological example of hepatocyte ballooning, ideally species-specific images (mouse vs. human) in high magnification. And please explain in detail how the scoring was applied and comparison to the AI derived data was performed - also taking expert control into account.

Answer:

Thank you for this suggestion. We have now added the methodology of NASH-CRN scoring in the Materials & Methods.

We followed the NASH-CRN scoring system for evaluation in human MASH[33], and no modification in the criteria was applied for evaluating the mouse liver, i.e., the pathologists applied the scoring to the mouse livers in the same way as they would in human cases.

Regarding hepatocyte ballooning, while the pathologists evaluated hepatocyte ballooning in our study, our AI-DP pipeline does not include AI quantification for ballooning. Hepatocyte

ballooning is a complex feature– a previous study showed that ballooning is a spectrum, and even in human liver specimens, pathologists' variability is quite high. In the previous study, 9 expert hepatopathologists were asked to annotate ballooned hepatocytes in 10 human liver biopsy slides. Collectively ~2400 hepatocytes were annotated; however, there was only 1 cell agreed by all 9 pathologists and 8 cells agreed by 8 pathologists, to be ballooned hepatocytes[34]. With regards to the question whether MASH animal models exhibit hepatocyte ballooning, our pathologists did identify ballooned cells in the mouse liver (please see figure R5 below). In addition, another previous study has also developed an automated MASH scoring in CCl4 and CDAHFD mouse models, which includes a ballooned score[35].

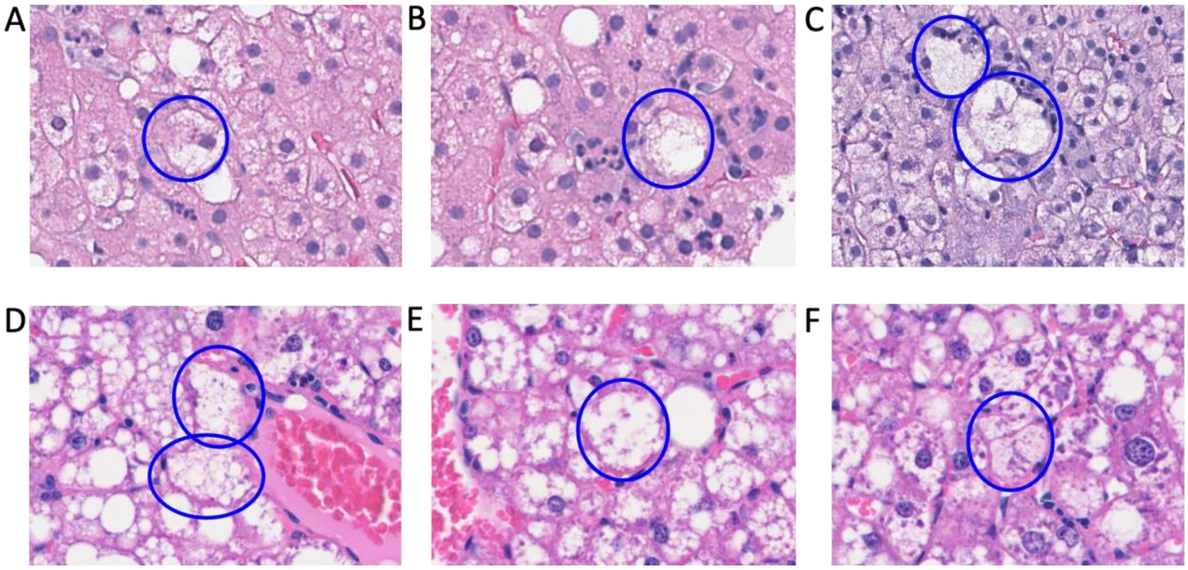

Figure R5. Pathologist annotation of ballooned hepatocytes in (A-C) human liver biopsy specimen, (D-F) MASH animal model.

Following the reviewer's suggestion, we added "Pathologist scoring" as a section in Materials & Methods:

"Pathologists evaluated steatosis (score: 0-3), hepatocyte ballooning (score: 0-2), lobular inflammation (score: 0-3), and fibrosis (score: 0-4), using H&E and PSR liver slides or WSIs, following the NASH-CRN scoring system, in the same paradigm as how the system is applied in human [33]. NASH activity score (NAS) was calculated as a sum of steatosis, hepatocyte ballooning, and lobular inflammation score. In addition, foamy pigment laden cells were also evaluated and assigned score 0-3 (0 : no foci, 1 : mild, 2 : moderate, 3 : severe). For validation of AI model, steatosis, inflammation, and fibrosis score were correlated with AI-DP-derived steatosis area, inflammatory cell density, and collagen intensity."

For validating our AI model development, we specifically looked at the correlation of our AI model with pathologists' score for steatosis, lobular inflammation, and fibrosis (refer to Table R1 below). To note, while we performed this correlation to serve as a means to validate AI model, the continuous nature of the AI quantification would allow for a more robust and precise quantification and larger window for evaluation of treatment effect, and the correlation alone will not necessarily be able to reflect this.

Table R1. The list of histopathology features correlated with AI-derived features.

Histopathology features	AI-derived features	Correlation (Spearman's R)	Figure in manuscript
Steatosis	Steatosis percent area	0.8	Added as a panel in Supplementary Fig. S10I
Lobular inflammation	Inflammatory cell cluster	0.84	Supplementary Fig. S4B
Fibrosis	SHG intensity	0.72	Supplementary Fig. S10H
PSR (%Area)	SHG intensity	0.92	Supplementary Fig. S10G

We have now added the detail of the correlation between the AI quantification and expert pathologist scoring, in Materials & Methods:

Subsection "SHG-TPE scanning & AI analysis of steatosis and fibrosis"

"The validation using the animal models in the current study (N=256 liver slides) showed that fibrosis signal generated by SHG correlate with the standard PSR area quantification (Pearson's R=0.92, Fig S10G) and pathologists' NASH-CRN fibrosis scores (Spearman's R=0.72; Fig S10H), while steatosis detection from the TPEF correlate with the pathologists' steatosis score (Spearman's R=0.80; Fig. S10I)."

Subsection "Inflammatory cell density"

"We also quantified the number of inflammatory cluster normalized to the tissue area via DBscan clustering (minimum number of cells = 5, maximum distance between neighbouring cells = 15 µm), similar to pathologist's evaluation of lobular inflammation. Inflammatory cell density and clusters showed a strong correlation with pathologist inflammation scores (Spearman's R = 0.82 for inflammatory cell density and Spearman's R = 0.84 for inflammatory cell foci, P<0.001; Fig. S4A-B)."

4. Shortly mention and explain the relationship of MASH and NASH, especially when referring to "NASH" scoring.
Answer:

MASH (Metabolic dysfunction-Associated Steatohepatitis) is a new nomenclature replacing NASH (Non-alcoholic steatohepatitis) [36], announced by the American Association for the Study of Liver Diseases (AASLD) in June 2023 (https://www.aasld.org/message-aasld-president-nomenclature-implementation). As such, we used MASH for all terms referring to the disease. However, when the terms used are related to an established nomenclature, such as "NASH-CRN", "NAS" score, we kept the term as is. Based on EASL and AASLD guidelines, NAS nomenclatures continue to be used for evaluating MASLD/MASH associated histopathology and the histologic diagnosis of steatohepatitis remains unchanged [37].

5. As we know that mouse and human liver tissues and pathologies harbor species-specific differences (e.g., amount of physiologic fibrous connective tissue) we strongly recommend to show AI applicability on human tissue as well to enable for a broader translatability.

Answer:

Indeed, mouse and human liver tissues and pathologies harbor species-specific differences. Our AI-DP pipeline was designed to detect various histopathological features of interests in MASH specimens such as the presence of lipid-droplets, inflammatory cells, fibrosis, as well as liver zonation based on central vein and portal vein landmarks, in mouse models. Our pipeline is applicable to human tissue; however, finetuning is needed to ensure optimal accuracy in these samples.

The two main modalities in our pipeline are SHG/TPEF image and H&E-stained image. The SHG/TPEF approach for MASH has been reported in multiple human studies including clinical trials (Matsuzaki et al. [12] and Akbary et al. [13]). The same pipeline as implemented here by HistoIndex has been applied to human samples; therefore, the application on human samples is relatively straight-forward. Our H&E-based approaches included the detection of lipid-droplets and inflammatory cells. Automated lipid-droplet detection has been explored in human samples for liver [14-17] and similar approach for inflammatory cell detection has been explored in human cancer samples [18].

The following paragraph has been added to Discussion:

"In our workflow, we combined HistoIndex SHG-based AI for sensitive fibrosis detection with an in-house H&E-based AI pipeline for features better resolved on H&E (e.g., inflammation, granulomas, and liver zonation). We then registered SHG and H&E modalities to enable zone-specific and colocalization analyses. Although optimized for mouse models, our approach is translatable to human-specimens or other experimental-models, with appropriate finetuning to account for species-specific histopathologic differences."

6. According to line 447ff, two different slide/sections were assessed by the pathologist and AI. In order to validate SHG, it has to be shown how classic histopathological evaluation and results of SHG applied AI correlate on the same slide.

Also SHG should be explained in more detail.

Answer:

SHG technology for evaluation of fibrosis has been validated in human studies [38]. We have also applied the technology (SHG-based AI DP platform) to assist pathologists in scoring fibrosis in MASH [39]. The technology has also been recognized as one of the emerging trends for fibrosis scoring in MASH, as reviewed by KOLs in Hepatology (Tetri-Neuschwander [40], Vlad Ratziu [41] and Arun Sanyal [42]).

SHG and PSR used for fibrosis scoring was obtained from adjacent sections of two different slides. However, we observed that the impression of fibrosis from the adjacent section is congruent. Please see the representative images below, which we have now added as panels

A-D in Supplementary Fig. S10. Moreover, we observed a strong correlation (r = 0.92) between SHG intensity and the pathologist's fibrosis score from PSR (Supplementary Fig. S10G) in our mouse model application. Please note that SHG imaging enhances visualization of fine, diffuse fibrosis, as demonstrated by MT and SHG images from the same section (MT performed post-SHG; now presented as panels E–F in Supplementary Fig. S10).

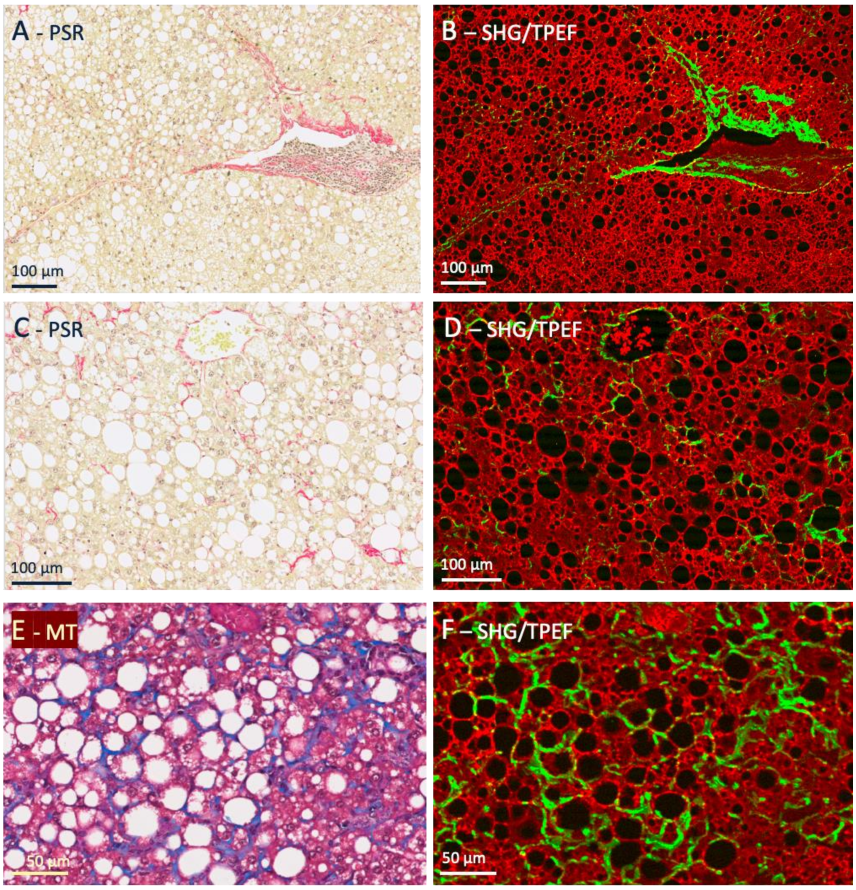

Supplementary Fig. S10A-F. (A-D) Comparisons of PSR with adjacent SHG, showing similar impression of fibrosis burden, (E-F) comparisons of MT and SHG on the same section shows enhanced visibility in SHG.

The explanation of SHG is included in the Materials & Methods Subsection **"SHG-TPE scanning & AI analysis of steatosis and fibrosis"**. We have now added two references covering the Physics of the technology [43,44].

"Unstained formalin-fixed paraffin-embedded (FFPE) tissue slides (section thickness: 5 µm) were scanned using the Genesis® 200 system (HistoIndex), which uses an ultrafast femtosecond laser, emitting photons to excite the unstained tissue sample at 780 nm. Second Harmonic Generation (SHG) signals at 390 nm and Two-Photon Excited Fluorescence (TPEF) signals at 550 nm were then collected at two photomultiplier tubes (PMT) [43,44].

7. Line 209ff: Differentiation between macro- and micro-steatosis and the liver zones does not contrast with conventional histopathology, as a pathologist can also differentiate zonal regions morphologically. The statement "This contrasts with conventional histopathology…" (line 210) is somehow misleading.

Answer:

Thank you for pointing this out. We meant to contrast the fact that AI-DP could provide a precise continuous scoring and the scoring can be applied specifically to the different liver zone, while in the NASH-CRN system for pathologist scoring in steatosis, the scoring is only semi-quantitative and categorical (score 1: steatosis <33% area, score 2: steatosis between 33-66% area, score 3: steatosis >66% area). We agree that pathologists are able to differentiate zonal regions morphologically, but quantifying the percentage of steatosis area robustly (either in the whole region or specific zone) could be challenging.

For clarity, we edited the text in the **Results** section as below:

"AI-DP can differentiate between macro- and micro-steatosis, and provide continuous scoring to evaluate steatotic burden across the liver's zonal microarchitecture. This contrasts with conventional histopathology, which, under the NASH-CRN framework, yields only ordinal, semi-quantitative categorical scores for whole-liver steatosis."

8. State and discuss the major advantages and benefits of AI-pathology over a pathologist. What about time needed to establish and conduct the AI digital pathology workflow versus classic microscopic histopathology? How easily can this workflow be transferred to other experimental setups/pathological conditions?

Answer:

We thank the reviewer for this thoughtful and important comment. Conventional microscopic histopathology remains the gold standard for liver disease assessment; however, AI-based DP offers several major advantages that complement expert evaluation. First, it enables objective, quantitative, and fully reproducible scoring of steatosis, inflammation, ballooning, and fibrosis, thereby reducing inter- and intra-observer variability. Second, once trained, the algorithm can analyze whole-slide images within minutes, allowing efficient, high-throughput evaluation of large experimental cohorts that would otherwise require extensive pathologist time. Third, AI models can detect subtle and spatially heterogeneous histological changes that may escape semi-quantitative manual scoring, thus improving sensitivity for early or partial treatment effects.

We acknowledge that the initial setup phase involving data curation, annotation, and model training requires a greater time investment than classical microscopy. However, once established, the workflow is highly efficient and scalable, enabling rapid analysis of new datasets with minimal additional effort. Importantly, the modular architecture of our pipeline allows straightforward transferability to other experimental setups or pathological conditions through limited retraining on representative annotated images. This flexibility makes the approach broadly applicable to diverse murine models of steatohepatitis, fibrosis, or metabolic injury.

We added the following into the **Discussion**:

"AI-enabled DP offers several major advantages that complement expert evaluation. First, it enables objective, quantitative, and fully reproducible scoring, thereby reducing inter- and

intra-observer variability. Second, once trained, the algorithm can analyze whole-slide images within minutes, allowing efficient, high-throughput evaluation of large experimental cohorts that would otherwise require extensive pathologist time. Third, AI models can detect subtle and spatially heterogeneous histological changes that may escape semi-quantitative manual scoring, thus improving sensitivity for early or partial treatment effects."

"… Although optimized for mouse models, our approach is translatable to human specimens or other experimental models, with appropriate finetuning to account for species-specific histopathologic differences."

Material and Methods

9. Line 448: It is described that fixation was achieved by formalin fixation for 24 hours followed by ethanol at room temperature. Is this to be regarded as representative fixation standard (for diagnostic human samples)? How about the influence of different fixation protocols (e.g., formalin only, shorter or longer fixation) on liver tissue histomorphology and subsequent influence on AI digital pathology analysis?

Answer:

Formalin fixation for 24 hours is a representative fixation method for routine histopathology [45]. Some lab might use 48 hours fixation. The fixation duration needs to be the same for samples from different batches to ensure comparability. After the fixation, tissue samples are processed through 70% to 100% ethanol gradient followed by clearing with xylene to remove water, ethanol and enable paraffin penetration.

Insufficient fixation can compromise tissue morphology due to incomplete preservation, whereas prolonged fixation may mask epitopes and reduce staining sensitivity (e.g., F4/80), impair special stains such as Masson's trichrome, and introduce artifacts that can interfere with both, conventional and AI-digital histopathology evaluation.

We added the reference in to the Materials & Methods:

"Livers from the euthanized mice were placed in 10% Neutral-Buffered Formalin for 24 hours [45] followed by transferring them to 70% EtOH at room temperature."

10. Line 454: Provide and specify used slide scanners and respective scanning resolutions

Answer:

For pathologist evaluation, all slides were digitally scanned at 20×, bright-field, using a NanoZoomer 2.0HT slide scanner (Hamamatsu Photonics). For AI model development, the slides were scanned at 40x using Vectra Polaris (Akoya Biosciences).

This detail has now been added into the **Materials & Methods ("Histology")**:

“All stained slides were examined by light microscopy and digitally scanned at 20× using NanoZoomer 2.0HT slide scanner (Hamamatsu Photonics) for pathologist evaluation.

Another slide was prepared for Second Harmonic Generation/Two Photon Excitation (SHG-TPE) scanning (HistoIndex, Singapore), followed by H&E staining, on which the multi-modal integrated AI DP pipeline was built. The H&E slides were then scanned at 40x using Vectra Polaris (Akoya Biosciences) to obtain whole slide images (WSIs).”

11. Provide additional information on murine liver sampling and trimming, especially specify which liver lobes/localizations were examined. Where there any anatomical predilection sites/lobes and any macroscopic findings?

Answer:

The entire left lateral liver lobe (the largest lobe) was submitted for histology. No macroscopic abnormalities were identified on gross examination during the study period reported in this manuscript.

This detail has also now been added into the Materials & Methods

Subsection “Histology”:

“The left lateral lobes of the livers were embedded in paraffin blocks and 5 μm thickness liver slides per sample were prepared.”

Figures

12. Figure 3. Inflammation quantification using AI digital pathology

- Add scale bars to the HE images (A-C)
- A: According to the image detail shown, the focally distributed immune cells seem to be intra-parenchymal. How was this lesion characterized and differentiated from common background findings in mouse liver (e.g., extramedullary hematopoiesis)?

  Answer:
  Scale bars have been added to the H&E images.

  Regarding extramedullary haematopoiesis, please refer to response to Reviewer 3 Q.1

13. Figure 4. Fibrosis quantification using AI digital pathology
o Please align maximum values on y-axis (C+D)

Answer:

Figure 4C-D has been edited so that the y-axis have the same range.

Similarly, Figure 5C-D as well.

14. Figure 5 and Figure 6

The collagen deposition shown in Fig. 5 (H) and Fig. 6 (I) appears very mild/not verifiable. Can MT/PSR be provided to confirm and show fibrosis, in addition to HE, SHG/TPE? (Fig. 5 H and Fig. 6 I)

Answer:

It has been established that SHG has a higher sensitivity compared to MT/PSR in visualizing fibrosis[40]. Figure 5R and Figure 6J are provided as an illustration for the colocalization methodology. We have also included a few images in Supplementary Figure S10 comparing SHG, MT and PSR stained images.

Reviewer #4 (Remarks to the Author):

I co-reviewed this manuscript with one of the reviewers who provided the listed reports. This is part of the Nature Communications initiative to facilitate training in peer review and to provide appropriate recognition for Early Career Researchers who co-review manuscripts.

Reviewer #5 (Remarks to the Author):

This study investigates murine models of metabolic dysfunction-associated steatohepatitis (MASH) and liver fibrosis using a multi-modal artificial intelligence (AI)-driven digital pathology pipeline. The study analyzes histology, blood chemistry, transcriptomics, lipidomics, and metabolomics to compare diet-induced models (e.g., Gubra Amylin NASH (GAN) and Choline Deficient Amino Acid-defined High Fat Diet (CDAHFD)) under different housing conditions (e.g., standard ambient (TA) or thermoneutral (TN)). The AI method quantifies zone-specific features such as micro- and macrosteatosis, inflammation, fibrosis, and granulomas, enabling colocalization analyses. Distinct model behaviors are described, with thermoneutral housing accelerating disease progression and certain diets producing more severe fibrosis. The manuscript further validates the AI approach in experiments with semaglutide, resmetirom, and an acetyl-CoA carboxylase inhibitor, highlighting differential treatment effects.

Specific comments:

1. Lines 122 and 124 describe that "housing animals at TN exacerbated disease progression and overall burden" and that "GAN-fed animals at TN reached Grade 3 steatosis in just 4 weeks, while those at TA took 8 weeks. By 16 weeks on GAN, 100% of TN-housed mice achieved the maximal score, compared to 28 weeks for TA-housed mice." In contrast, Line 148 states that "the GAN diet induced fewer transcriptional changes at TN than at TA by week 12" and that "transcriptional changes worsened between weeks 16 and 32 at TN, with the most significant difference at week 23." Based on this information, the pronounced gene expression transitions in GAN-fed animals under TN relative to TA appear to occur after 16 weeks, whereas TN-related disease progression is evident much earlier. This temporal mismatch warrants further explanation to reconcile the early phenotypic exacerbation with the delayed transcriptional divergence.

Answer:

Thank you - this is an important point. The apparent temporal mismatch reflects differences in sampling frequency between conventional histology and transcriptomics rather than a true biological contradiction. We performed temporal histopathology at weeks 0, 4, 8, 12, 16, 23 and 32, which enabled detection of the early phenotypic exacerbation of steatosis in GAN-fed mice housed at TN (Grade 3 by 4 weeks at TN vs 8 weeks at TA, and 100% TN at Grade 3 by 16 weeks). By contrast, bulk RNA-seq was only performed at selected later time points (weeks 12, 16, 23 and 32) and therefore, do not provide direct transcriptional readout for the earliest histologic changes (weeks 4–8).

Additionally, animals housed at TA experience mild cold stress relative to TN, which shifts baseline gene expression. TA housing is associated with modulation of pathways involved in metabolic flux, thyroid hormone activation, lipid mobilization, circadian rhythm, mitochondrial function and adrenergic signalling [46-50]. As also supported in our transcriptomic analysis, this chronic sympathetic/adrenergic GPCR activation in the liver (a canonical molecular response to below-thermoneutral housing-induced cold stress) is reflected through upregulation of *Adrb2/Adra1a* with downstream G-protein *(Gnai1/2, Gnaq)*, cAMP generation *(Adcy3)*, PKA activation *(Prkacb)*, and CREB transcriptional signaling *(Creb1)*. These TA-related transcriptional adaptations amplify the differential transcriptional background and can obscure early diet-induced changes at TN. Thus, the apparently smaller number of diet-driven transcriptional changes at TN versus TA at week 12 likely reflects a combination of absence of RNA-seq sampling during the earliest histologic changes and persistent TA-specific transcriptional differences driven by thermal stress that affect the baseline against which diet effects are measured.

While resolving the precise temporal relationship between early histologic worsening and early transcriptional events will require additional molecular sampling at earlier time points (e.g., weeks 4–8 or before) and/or spatially resolved molecular assays co-registered to histology, we showed that the early phenotypic exacerbation at TN is real and captured by our histology time course; and the transcriptional changes align with histopathological findings at later timepoints as the impact of disease aetiology becomes dominant. The comparison at 23 weeks, where the transcriptomic changes peaked for TN, showed a higher number of differentially expressed (DE) genes at TN (1305 DE genes) compared to TA (836 DE genes).

We also observed that metabolically relevant signatures were exclusive to TN, highlighting the TN model being the more metabolically relevant model compared to TA.

We added the following points to the Results subsection ***"Key insights from histopathology on environmental influences in MASLD/MASH progression"***:

"Bulk RNA seq corroborated these observations. GAN diet induced transcriptional changes at TN which worsened from week 16 to week 32, peaked at week 23 with 1305 Differentially expressed (DE) genes at TN vs. 836 DE genes at TA, followed by deceleration in changes thereafter (Fig. S1A). Additionally, KEGG and Gene Ontology (GO) enrichment analyses highlighted unique signatures for each housing conditions. While cardiac hypertrophy, fatty acid degradation, and inflammatory bowel disease were linked to TA, metabolically relevant signatures for Type II Diabetes, inflammasome activity, and fatty acid elongation were exclusive to TN (Fig. S1B)."

2. Lines 239 – 240: "The models achieved ROC AUC/PR AUC scores of 0.82/0.55 and 0.92/0.93, respectively." Please provide further clarification on the gold standards used to identify inflammatory cells in normal hepatocyte areas and steatotic regions.

Answer:

The AI model for the inflammatory cell detection was developed based on pathologist's annotation of inflammatory cells as mentioned in response to Reviewer 1 Q7. The pathologists were provided with image patches and were asked to annotate all the different cells in the patches. For the model developed for the 40x WSI, the model was trained on 161 patches of size 526 x 526 at 40x magnification (equivalent to 131.5 µm x 131.5 µm), comprising 10176 cells, in which 4398 are inflammatory cells. The patches originated from GAN as well as CDAHFD models, comprising both steatotic and non-steatotic area. The model was tested on 42 independent patches containing 1123 non-inflammatory cells and 1084 inflammatory cells. The models achieve ROC AUC value of 0.94 and PR AUC value of 0.93.

For clarity, we added the following details in the Materials & Methods, subsection **"Inflammatory cell detection":**

"In total, we trained our model on 161 patches of size 526 x 526 at 40x magnification comprising 10176 cells, for which 4398 are inflammatory cells, on both steatotic as well as non-steatotic areas."

We would also like to point out that two models were built – one for the images scanned at 40x and one for 20x, as some of the datasets (GAN model in Figure 3) were only available at 20x. The performance for the 20x is lower (ROC AUC/PR AUC 0.82/0.55) compared to the 40x (ROC AUC/PR AUC 0.92/0.93), as the 20x model was trained on less number of annotation (at 40x: ~4000 cells, at 20x: ~1000 cells) and the quality annotation at 20x was also not as high as the 40x because the pathologist could not reliably identify the cells at 20x, so we had a higher number of cells annotated as "uncertain". Following the comment by Reviewer 2 Q4, we re-cut, stained, and scanned these images at 40x, using the same setup

as that for the 40x model, and run the high performance 40x model instead. In the revised manuscript, we have removed the 20x model and adhered to the 40x model for consistency.

To further validate our model, we have also included additional experiments using multiplexed-IF for an orthogonal validation. The multiplexed-IF (mIF) was performed on two different sections: one section with T-cell panel (i.e., CD3, CD4, CD8) and another section with macrophage panel (i.e., F4/80, CD68, CD11b, and CD44). H&E was performed on the same sections post-mIF. We assume that the sum of all markers (T-cells and macrophages) to be closer to the total inflammatory cell population that we can expect in the liver tissue. Indeed, we showed that the inflammatory cells being picked up by our AI model are highly correlated (r=0.83) with orthogonal validation via mIF (added as panel B in Supplementary Figure S4).

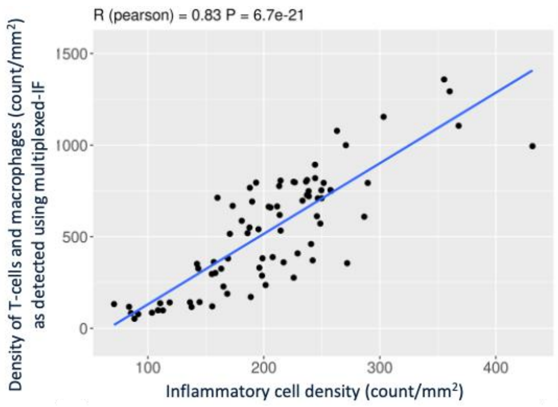

Supplementary Figure S4B. The correlation between inflammatory cell density as detected by AI model on H&E sections and the density of all T-cells (CD3+, CD4+, or CD8+) and macrophages (F4/80+, CD11b+, CD68+, or CD44+) as detected on multiplexed-IF. The T-cell panel and macrophage panel were performed on a different section; H&E was performed on the same section post-mIF. The H&E-based inflammatory cell density (x-axis) was the average of the inflammatory cells detected on the two corresponding H&E sections.

3. Lines 279 – 280: "To assess whether liver fibrosis development in MASH models is metabolically driven, we quantified the colocalization of steatosis and fibrosis using SHG/TPEF images".
Please provide a clear definition of "colocalization" of steatosis and fibrosis and how it is implemented in the AI model training process.

Answer:

The colocalization of steatosis and fibrosis specifically refers to the spatial association between lipid-droplets in liver and collagen fibres within a specific surrounding range. In this study, it focuses on quantifying fibrotic changes in the region adjacent to fat vacuoles in the tissue samples. Followed the range mentioned in a previous study [24], fibrosis within 14 μm around fat vacuoles are considered as colocalized with steatosis in our analysis. This colocalization analysis itself is an image processing operation downstream to the detection of steatosis and fibrosis by AI/ML algorithm, so no AI model training was involved.

For clarity, we have included this information in the Materials & Methods for clarity, in the subsection **"SHG/TPEF scanning & AI analysis of steatosis and fibrosis"**:

"Image analysis was then applied to quantify zone-specific features comprising a total of 100 fibrosis features and 45 steatosis features (Supplementary Table S3-S4)[51], as well as the colocalization between fibrosis and steatosis defined by fibrosis present within 14 µm around the fat vacuoles [52]."

4. Lines 312 – 314: "Colocalization analysis indicated a correlation between pigmented granulomas and fibrosis development (Fig. 6I-J), suggesting a potential link to disease etiology."
Please elaborate on this statement by providing supporting evidence on the potential causal links in etiology beyond the observed association.

Answer:

The pigmented granulomas shown in Fig 6 were one of the two distinct types of hepatic granulomas observed in our murine MASH models (GAN diet–induced and CDAHFD-induced). The pigmented granulomas were specifically observed in the CDAHFD-induced models, where macrophages clustered as slightly pale, foamy, pigmented aggregates. In the GAN model, mononuclear cells and macrophages arranged around steatotic hepatocytes in a crown-like pattern (known as lipogranuloma).

When hepatocellular lipid release exceeds the clearance capacity of resident phagocytes, excess lipid is sequestered by macrophages, producing lipogranulomas [6]. Pigmented granulomas arise by a similar mechanism but show additional pigment accumulation (ceroid/lipofuscin) and often iron deposition [7]. These granulomas therefore reflect ongoing lipid handling/stress and macrophage activation (CD44+, CD68+) within the liver [6,8]. Such lesions are associated with chronic inflammation and have been linked to increased risk of progression to hepatocellular carcinoma (HCC) in chronic steatohepatitis models and human disease [9]. The anatomical location of granulomas can provide etiologic clues (for example, portal granulomas are more typical of primary biliary cholangitis, whereas viral-related granulomas tend to be parenchymal), but substantial overlap exists and location alone is not diagnostic [6]. Granulomas may regress if the underlying cause is treated; however, their fate depends on disease context, duration of injury and extent of fibrosis.

Notably, in our dataset, we observed that pigmented granuloma starts to correlate after inflammation reached a certain level – in this case after week 4 in the CDAHFD model (added as Panel B in Supplementary Figure S6), supporting the association with chronic inflammation. Pigmented granuloma prevalence correlated strongly with liver fibrosis in the CDAHFD model (added as Panel C in Supplementary Figure S6), supporting a link between pigment-laden macrophage aggregates and fibrogenic progression in this dietary model of MASH. Additionally, we also performed multiplexed-IF experiments using macrophage markers CD44, CD68, CD11b, and F4/80. Our data showed that CD44+ cells are the main infiltrates of pigmented granuloma, with 10-fold higher presence compared to the second majority which is CD68+ cells (added as Panel E in Figure6).

We added the biological explanation of pigmented granuloma and additional data in the Results subsection **"AI-DP for evaluation of pigmented granulomas and lipogranulomas**

in CDAHFD and GAN models of MASH and liver fibrosis", Figure 6, and Supplementary Figure S6:

"We temporally evaluated pigmented granulomas using AI-DP (Fig. 6F-H), noting an increase in lesion area over time. In the CDAHFD model, factors such as age (2.1-fold higher in aged vs. young mice at week 8, P=0.015; Fig 6F and 6H) and housing conditions (3-fold higher at TN vs. TA at week 8, P<0.001; Fig 6F-G) influenced granuloma growth. Pigment granuloma starts to correlate with inflammation after week 4 (Pearson's R=0.51, P=0.016, Supplementary Fig. S6B), supporting the association with chronic inflammation [7]. We observed an increasing time-dependent prevalence of CD44+ cells in the pigmented granuloma, which was about 8-fold at week 8 compared to CD68+ cells as the second highest population within the pigmented granuloma (Fig. 6E, I). This supports previous reports that granulomas reflect ongoing lipid handling/stress and macrophage activation (specifically CD44+ and CD68+) within the liver [6,8]. Notably, pigmented granuloma prevalence correlated strongly with liver fibrosis (Pearson's R=0.75, P<0.001, Supplementary Fig. S6C), which is supported by the colocalization analysis between pigmented granuloma and fibrosis (Fig. 6J-M)."

Addition to Figure 6:

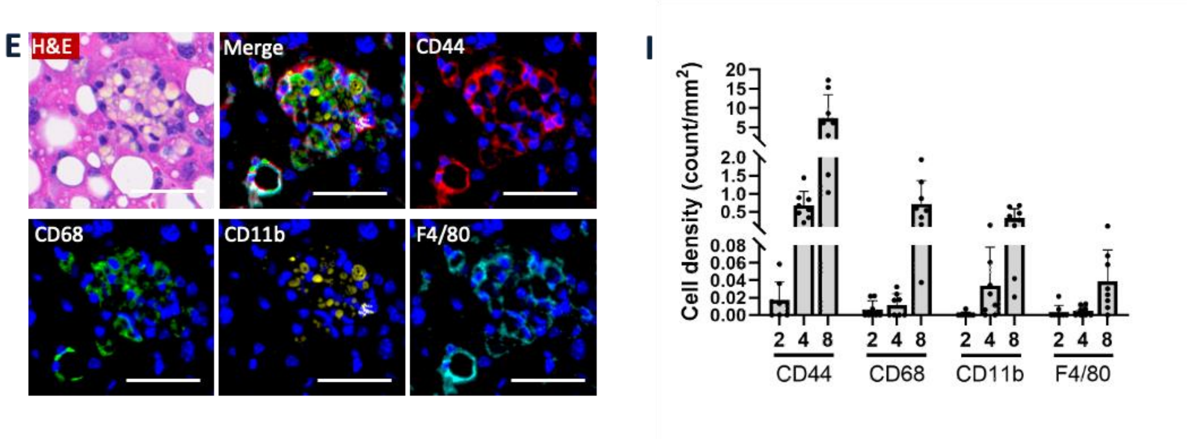

Figure 6. (E) Multiplexed-IF for macrophage panel (CD44,CD68, CD11b, F4/80), along with the H&E on the same section, further demonstrates the presence of macrophages in the pigmented granuloma. (I) Contribution of the different macrophages to the pigmented granuloma.

Addition to Supplementary Figure S6:

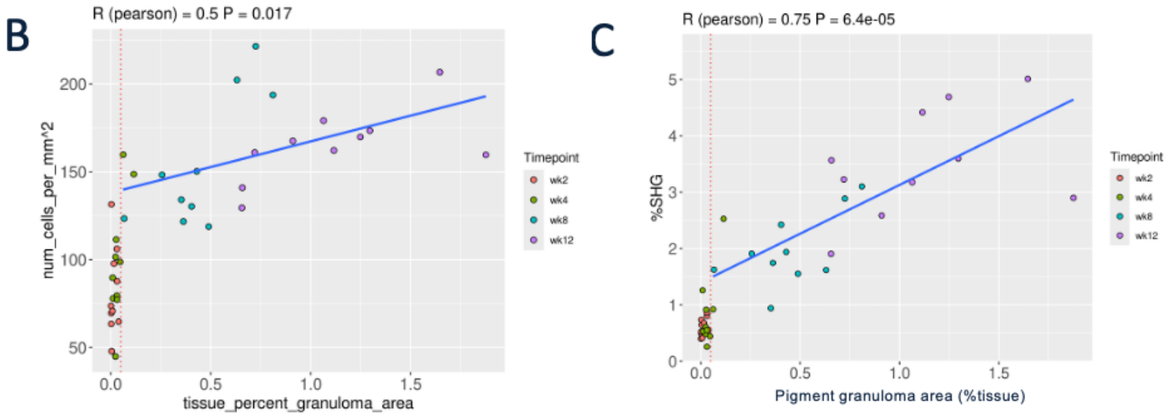

Figure S6. (B) Correlation between AI-derived pigment granuloma area and inflammation, (C) Correlation between AI-derived pigment granuloma area and fibrosis. Data are from CDAHFD-TA young; N=40, correlation was performed for data points with pigmented granuloma area larger than 0.05% tissue (generally data point after week 4).

5. Lines 488 – 489: "All Omics data was analyzed using R. Transcriptomics was analyzed with DESeq2, lipidomics was with package of lipidr while metabolomics utilized omu and MetaboAnalystR." Please elaborate on the parameters used in these analyses, including sensitivity tests and multiple test correction conducted.

Answer:

For transcriptomics data, differential express analysis was performed with DESeq2, using the cutoff of log2(FC)>1 or log2(FC)<-1, and Padj<0.05. For lipidomics data, differential altered lipids were identified with R package of lipidr, using the cutoff of log2(FC)>2 or log2(FC)<-2, and Padj<0.05. For metabolomics data, differential altered metabolites were identified with R packages of omu and MetaboAnalystR, using the cutoff of log2(FC)>2 or log2(FC)<-2, and Padj<0.05. All the 3 multiple test corrections were using Benjamini-Hochberg (BH) approach.

We have now included the parameters in the Materials & Methods subsection **"Murine transcriptomics datasets"** :

"All Omics data was analyzed using R. For transcriptomics data, differential express analysis was performed with DESeq2, using the cutoff of log2(FC)>1 or log2(FC)<-1, and Padj<0.05. For lipidomics data, differential altered lipids were identified with R package of lipidr, using the cutoff of log2(FC)>2 or log2(FC)<-2, and Padj<0.05. For metabolomics data, differential altered metabolites were identified with R packages of omu and MetaboAnalystR, using the cutoff of log2(FC)>2 or log2(FC)<-2, and Padj<0.05. All the 3 multiple test corrections were using Benjamini-Hochberg (BH) approach."

6. Lines 504 – 506: "AI-based image analysis was performed to identify liver zone-specific (Zone 3/CV, Zone 2/PS, Zone 1/PT) features comprising a total of 100 fibrosis features and 45 steatosis features, as well as the colocalization between fibrosis and steatosis." Please provide further information on the AI model training process, including slide amount, internal or external validation, and categorization of the fibrosis/ steatosis features. Please include performance evaluation metrics of the AI model.

Answer:

The validation of the AI model for the SHG/TPEF image in human has been reported in Liu et al. Hepatology 2020 [38]. For the application on mouse model, the AI/ML algorithm as implemented and validated in human samples was applied for mouse samples. In short, the AI/ML algorithm was mainly used to classify lumen structures within liver tissue, with structure identification relying on second-harmonic generation/two-photon excited fluorescence (SHG/TPEF) microscopic imaging technology, as lumens appeared as black holes in TPEF channel without any fluorescence signal and surrounded by SHG signal from structure collagen in lumen wall. This method achieves full-process automation from signal capture to structure classification. For the detection of liver zone, the Classification and Regression Tree (CART) algorithm was used to distinguish the two lobule landmarks: CV and PT. For mouse application, the evaluation for this CV/PT classification model was performed on 1081 lumen structures from multiple mice MASH samples, resulted an accuracy of 0.81 (CV-Precision 0.82, CV-Recall 0.92, PT-Precision 0.81 and PT-Recall 0.61).

The CART was also applied for steatosis detection based on the lumen in TPEF channel, while the detection of fibrosis was based on Otsu thresholding in the SHG channel [38]. Image analysis was applied to quantify 100 features in fibrosis and 45 features in steatosis, with the following categorizations (added as Supplementary Table S3 and S4).

Supplementary Table S3. Categorization of steatosis features derived from SHG/TPEF images

Categories	Measurements	Regions
Steatosis	Percentage of steatosis area	Overall, CV, PS, PT
Macrosteatosis	Percentage of macrosteatosis area	
Fat vacuoles (FV)	Number of FV<br>Percentage of hepatocytes with FV<br>Average distance between one FV and the closest FV<br>Variance distance between one FV and the closest FV<br>Average number of FVs within 100 µm of a FV<br>Variance number of FVs within 100 µm of a FV	
Macro FV	Number of macro FV	

Supplementary Table S4. Categorization of fibrosis features derived from SHG/TPEF images

Categories	Measurements	Regions
Total collagen	Percentage of total collagen	Overall, CV, PS, PT
Aggregated collagen	Percentage of aggregated collagen	
Distributed collagen	Percentage of distributed collagen	
Total strings, aggregated strings, distributed strings	Number of strings<br>Number of short strings<br>Number of long strings<br>Number of thin strings<br>Number of thick strings<br>String area<br>String length<br>String width	Overall, CV, PS, PT
Total strings	String eccentricity<br>String solidity<br>String perimeter<br>String orientation	Overall
	Number of intersections	Overall, CV, PS, PT

The validation using animal models showed that fibrosis signal generated by SHG correlate with the standard PSR area quantification (Pearson's R=0.92, Supplementary Fig. S10G) and pathologists' MASH-CRN fibrosis scores (Spearman's R=0.71, Supplementary Fig. S10H),

while steatosis detection from the TPEF correlate with the pathologists' steatosis score (Spearman's R=0.80; Supplementary Fig. S10I).

We have added the following detail in the **Materials & Methods ("SHG-TPE scanning & AI analysis of steatosis and fibrosis"** and added **Supplementary Table S3 and S4**:

"AI/ML algorithm as implemented and validated in human samples [38] was applied for mouse samples for identification of liver zone (Zone 3/CV, Zone 2/PS, Zone 1/PT) based on CV and PT landmarks, along with steatosis and fibrosis. Image analysis was then applied to quantify zone-specific features comprising a total of 100 fibrosis features and 45 steatosis features (Supplementary Table S4-S5)[51], as well as the colocalization between fibrosis and steatosis defined by fibrosis present within 14 µm around the fat vacuoles [52]. For mouse application, the CV/PT classification model was performed on 1081 lumen structures from 9 animal liver slides, resulted an accuracy of 0.81 (CV-Precision 0.82, CV-Recall 0.92, PT-Precision 0.81 and PT-Recall 0.61). The validation using the animal models in the current study (N=256 liver slides) showed that fibrosis signal generated by SHG correlate with the standard PSR area quantification (Pearson's R=0.92, Fig. S10G) and pathologists' NASH-CRN fibrosis scores (Spearman's R=0.72, Fig. S10H), while steatosis detection from the TPEF correlate with the pathologists' steatosis score (Spearman's R=0.80; Fig. S10I)."

7. It would be helpful to compare the pros and cons of the multi-modal digital pathology in detecting liver zones in this study, compared to other existing AI models such as AIM-MASH, FibroNest, MorphoQuant, or Histoindex.

Answer:

The existing AI models for a study in the liver as mentioned by the reviewers, AIM-MASH (by PathAI), FibroNest (by PharmaNest), MorphoQuant, or HistoHepa (by HistoIndex), have their own pros and cons, which have been reviewed thoroughly in Vlad et al. J Hep 2024 [41]. In relation to our approach, we would like to highlight two points of consideration when evaluating vendors' approaches or algorithms: (1) modality used, (2) analysis type.

Modality used: *Do the algorithms utilize stained-based images or unstained sections?* Most vendors/algorithms typically utilize stained-based images (H&E, MT, PSR), while HistoIndex leverages the high sensitivity of fibrosis detection by SHG technique[40,43] on an unstained tissue section.

Analysis type: *What types of analysis do the algorithms provide – scores, feature annotations, colocalization analysis, and liver zone-specific analysis?* While all vendors provide analysis of all standard readouts in MASH (i.e., steatosis, ballooning, inflammation, and fibrosis), not all vendors have the readouts specifically for zone analysis. As spatial localization is important in MASH, having specific analysis for each zone will allow more investigation disease biology or drug's mechanisms of actions.

Our approach: our multi-modal pipeline combines the highly sensitive SHG/TPEF approach for fibrosis via HistoIndex platform and combined it with in-house AI pipeline for the H&E (or IHC images) on the same section for a more robust quantification of inflammation, including liver zone-detection based on H&E. Steatosis analysis can be done either via HistoIndex

platform or the in-house pipeline; however, for the evaluation of inflammation (and ballooning), stained-images like H&E will provide a more reliable readout as the model will be able to evaluate the features in a greater detail as in the SHG/TPEF images this appears black. In the downstream analysis, our pipeline includes registration of the two modalities (SHG/TPEF and stained images) to allow colocalization analysis between features (e.g., fibrosis/steatosis, fibrosis/inflammation). Another point to note is that most of available models are currently focus on clinical applications, while our in-house models are specifically finetuned for preclinical applications in mouse models.

We added the following paragraph in the **Discussion**, and also included the comparisons for the different AI models as **Supplementary Table S3**.

"AI-enabled DP has also increasingly been applied to human MASH, where liver biopsy remains the diagnostic and treatment evaluation gold standard. Platforms such as AIM-MASH[53], FibroNest[54], and HistoIndex[38] provide continuous, quantitative scores of histopathologic features, addressing limitations of conventional semiquantitative scoring, with each platform having distinct capabilities [41]. In our workflow, we combined HistoIndex SHG-based AI for sensitive fibrosis detection with an in-house H&E-based AI pipeline for features better resolved on H&E (e.g., inflammation, granulomas, and liver zonation). We then registered SHG and H&E modalities to enable zone-specific and colocalization analyses. Although optimized for mouse models, our approach is translatable to human specimens or other experimental models, with appropriate finetuning to account for species-specific histopathologic differences."

Table S3. Categorization of steatosis features derived from SHG/TPEF images

Categories	Measurements	Regions
Steatosis	Percentage of steatosis area	
Macrosteatosis	Percentage of macrosteatosis area	
Fat vacuoles (FV)	Number of FV	
	Percentage of hepatocytes with FV	
	Average distance between one FV and the closest FV	Overall, CV, PS, PT
	Variance distance between one FV and the closest FV	
	Average number of FVs within 100 μm of a FV	
	Variance number of FVs within 100 μm of a FV	
Macro FV	Number of macro FV	

8. Could the authors expand on the potential applications of their murine-based AI digital liver pathology tool for assessing liver pathology in humans and other organisms? It would be helpful to incorporate this perspective in the Discussion section.

Answer:

Our AI-driven DP pipeline detects key histopathological features in MASH specimens, including lipid droplets, inflammatory cells, fibrosis, and liver zonation based on central and portal vein landmarks. The pipeline is optimized for mouse models, and direct application to human samples requires fine-tuning to ensure optimal accuracy.

The pipeline integrates two primary modalities: SHG/TPEF imaging and H&E-stained imaging. SHG/TPEF for MASH has been validated in multiple human studies, including clinical trials (Matsuzaki et al. [12] and Akbary et al. [13] among many others). The same HistoIndex-based implementation as used in this study, has been applied to human samples, facilitating straightforward translation. Our H&E-based methods include automated detection of lipid droplets and inflammatory cells. Automated lipid-droplet detection has been reported in human liver studies [14,15,17] and analogous approaches for inflammatory cell detection have been demonstrated in human cancer samples [18].

We have added the following to provide perspective in the **Discussion**:

"In our pipeline, we leveraged HistoIndex AI-DP for a sensitive detection of fibrosis by SHG technology, combined with in-house AI pipeline for detection of features that can be more reliably identified on H&E images such as inflammation or granuloma. While our pipeline was optimized for the application in mouse models, the approaches can also be translated for application in human specimens, with finetuning to ensure an optimal accuracy as there are species-specific differences in the appearance of the histopathological features in the tissue."

Reviewer #5 (Remarks on code availability):

The code provides a README file with instructions for installing and running the application.

Answer:

We thank the reviewer for taking the time to review our code repository. Apart from the readme file with the instruction for installing and running the application, we have now also uploaded representative WSI as well as intermediate files so that the implementation will become easier.

Reviewer #6 (Remarks to the Author):

I co-reviewed this manuscript with one of the reviewers who provided the listed reports. This is part of the Nature Communications initiative to facilitate training in peer review and to provide appropriate recognition for Early Career Researchers who co-review manuscripts.

References

1. Ganz, M., Csak, T. & Szabo, G. High fat diet feeding results in gender specific steatohepatitis and inflammasome activation. *World Journal of Gastroenterology* **20**, 8525–8534 (2014).
2. Meyer, J., *et al.* Sex differences in diet-induced MASLD – are female mice naturally protected? *Frontiers in Endocrinology* **Volume 16 - 2025**(2025).

3. Hla, T. & Dannenberg, Andrew J. Sphingolipid Signaling in Metabolic Disorders. *Cell Metabolism* **16**, 420–434 (2012).
4. Rom, O., *et al.* Glycine-based treatment ameliorates NAFLD by modulating fatty acid oxidation, glutathione synthesis, and the gut microbiome. *Science Translational Medicine* **12**, eaaz2841 (2020).
5. Shen, J., *et al.* Essentiality of SLC7A11-mediated nonessential amino acids in MASLD. *Science Bulletin* **69**, 3700–3716 (2024).
6. Almadi, M.A., *et al.* New insights into gastrointestinal and hepatic granulomatous disorders. *Nature Reviews Gastroenterology & Hepatology* **8**, 455–466 (2011).
7. Sobczak-Filipiak, M., *et al.* Lipogranulomas and pigment granulomas in livers of dogs with portosystemic shunt. *Polish Journal of Veterinary Sciences* **vol. 21**, 265–272 (2018).
8. Zaki, M.Y.W., *et al.* Key features of the environment promoting liver cancer in the absence of cirrhosis. *Scientific Reports* **11**, 16727 (2021).
9. Zhu, H., Jr, H.C., Clain, D., Min, A. & Theise, N. Hepatic lipogranulomas in patients with chronic liver disease: Association with hepatitis C and fatty liver disease. *World Journal of Gastroenterology* **16**, 5065–5069 (2010).
10. Kim, C.-W., *et al.* Acetyl CoA Carboxylase Inhibition Reduces Hepatic Steatosis but Elevates Plasma Triglycerides in Mice and Humans: A Bedside to Bench Investigation. *Cell Metabolism* **26**, 394–406.e396 (2017).
11. pocai, a. Differential Effects of De Novo Lipogenesis Inhibitors on plasma lipids and liver PNPLA3. *The FASEB Journal* **33**, 487.417–487.417 (2019).
12. Matsuzaki, S., *et al.* Quantification of collagen fiber properties in alcoholic liver fibrosis using polarization-resolved second harmonic generation microscopy. *Scientific Reports* **13**, 22100 (2023).
13. Akbary, K., Noureddin, M., Yayun, R., Tai, D. & Boudes, P. Development of AI Based Fibrosis Detection Algorithm by SHG/TPEF Microscopy for Fully Quantified Liver Fibrosis Assessment in MASH. *Liver International* **45**, e70258 (2025).
14. Nativ, N.I., *et al.* Automated image analysis method for detecting and quantifying macrovesicular steatosis in hematoxylin and eosin–stained histology images of human livers. *Liver Transplantation* **20**, 228–236 (2014).
15. Roy, M., *et al.* Deep-learning-based accurate hepatic steatosis quantification for histological assessment of liver biopsies. *Laboratory Investigation* **100**, 1367–1383 (2020).
16. Guo, X., Wang, F., Teodoro, G., Farris, A.B. & Kong, J. LIVER STEATOSIS SEGMENTATION WITH DEEP LEARNING METHODS. *Proc IEEE Int Symp Biomed Imaging* **2019**, 24–27 (2019).
17. Mizuochi, S., *et al.* Deep-Learning-Aided Quantification of Steatohepatitis-Associated Pathological Findings in Liver Specimens. *Hepatology Research* **55**, 1462–1470 (2025).
18. Gamper, J., *et al.* Pannuke dataset extension, insights and baselines. *arXiv preprint arXiv:2003.10778* (2020).
19. Yersiz, H., *et al.* Assessment of hepatic steatosis by transplant surgeon and expert pathologist: A prospective, double-blind evaluation of 201 donor livers. *Liver Transplantation* **19**, 437–449 (2013).
20. Gambella, A., *et al.* Improved assessment of donor liver steatosis using Banff consensus recommendations and deep learning algorithms. *Journal of Hepatology* **80**, 495–504 (2024).
21. Takahashi, Y. & Fukusato, T. Histopathology of nonalcoholic fatty liver disease/nonalcoholic steatohepatitis. *World Journal of Gastroenterology* **20**, 15539–15548 (2014).
22. Murphy, S., Martin, S. & Parton, R.G. Quantitative Analysis of Lipid Droplet Fusion: Inefficient Steady State Fusion but Rapid Stimulation by Chemical Fusogens. *PLOS ONE* **5**, e15030 (2010).

23. Liu, C. & Crawford, J.M. Liver, Anatomy. in *Encyclopedia of Gastroenterology* (ed. Johnson, L.R.) 510–515 (Elsevier, New York, 2004).
24. Wang, X.-X.*, et al.* Collagen co-localized with macrovesicular steatosis better differentiates fibrosis progression in non-alcoholic fatty liver disease mouse models. *Frontiers in Medicine* **Volume 10 - 2023**(2023).
25. Zhang, Z.*, et al.* Gubra Amylin-NASH Diet Induced Nonalcoholic Fatty Liver Disease Associated with Histological Damage, Oxidative Stress, Immune Disorders, Gut Microbiota, and Its Metabolic Dysbiosis in Colon. *Molecular Nutrition & Food Research* **68**, 2300845 (2024).
26. Yamada, A.*, et al.* Longitudinal Analysis of Mitochondrial Function in a Choline-Deficient L-Amino Acid-Defined High-Fat Diet-Induced Metabolic Dysfunction-Associated Steatohepatitis Mouse Model. *Int J Mol Sci* **25**(2024).
27. Sakaguchi, T.*, et al.* Novel Choline-Deficient and 0.1%-Methionine-Added High-Fat Diet Induces Burned-Out Metabolic-Dysfunction-Associated Steatohepatitis with Inflammation by Rapid Immune Cell Infiltration on Male Mice. *Nutrients* **16**, 4151 (2024).
28. Sugasawa, T.*, et al.* One Week of CDAHFD Induces Steatohepatitis and Mitochondrial Dysfunction with Oxidative Stress in Liver. *International Journal of Molecular Sciences* **22**, 5851 (2021).
29. Gluchowski, N.L., Becuwe, M., Walther, T.C. & Farese, R.V. Lipid droplets and liver disease: from basic biology to clinical implications. *Nature Reviews Gastroenterology & Hepatology* **14**, 343–355 (2017).
30. Mashek, D.G. Hepatic lipid droplets: A balancing act between energy storage and metabolic dysfunction in NAFLD. *Molecular Metabolism* **50**, 101115 (2021).
31. Kusminski, C.M. & Scherer, P.E. New zoning laws enforced by glucagon. *Proceedings of the National Academy of Sciences* **115**, 4308–4310 (2018).
32. Africa, J.A.*, et al.* In Children With Nonalcoholic Fatty Liver Disease, Zone 1 Steatosis Is Associated With Advanced Fibrosis. *Clinical Gastroenterology and Hepatology* **16**, 438–446.e431 (2018).
33. Kleiner, D.E.*, et al.* Design and validation of a histological scoring system for nonalcoholic fatty liver disease†. *Hepatology* **41**, 1313–1321 (2005).
34. Brunt, E.M.*, et al.* Complexity of ballooned hepatocyte feature recognition: Defining a training atlas for artificial intelligence-based imaging in NAFLD. *Journal of Hepatology* **76**, 1030–1041 (2022).
35. Heinemann, F., Birk, G. & Stierstorfer, B. Deep learning enables pathologist-like scoring of NASH models. *Scientific Reports* **9**, 18454 (2019).
36. Rinella, M.E. & Sookoian, S. From NAFLD to MASLD: updated naming and diagnosis criteria for fatty liver disease. *J Lipid Res* **65**, 100485 (2024).
37. Tacke, F.*, et al.* EASL–EASD–EASO Clinical Practice Guidelines on the management of metabolic dysfunction-associated steatotic liver disease (MASLD). *Journal of Hepatology* **81**, 492–542 (2024).
38. Liu, F.*, et al.* qFIBS: An Automated Technique for Quantitative Evaluation of Fibrosis, Inflammation, Ballooning, and Steatosis in Patients With Nonalcoholic Steatohepatitis. *Hepatology* **71**, 1953–1966 (2020).
39. Abdurrachim, D.*, et al.* Utility of AI digital pathology as an aid for pathologists scoring fibrosis in MASH. *Journal of Hepatology* **82**, 898–908 (2025).
40. Neuschwander-Tetri, B.A., Akbary, K., Carpenter, D.H., Noureddin, M. & Alkhouri, N. The emerging role of second harmonic generation/two photon excitation for precision digital analysis of liver fibrosis in MASH clinical trials. *Journal of Hepatology* **83**, 790–799 (2025).
41. Ratziu, V.*, et al.* Artificial intelligence-assisted digital pathology for non-alcoholic steatohepatitis: current status and future directions. *Journal of Hepatology* **80**, 335–351 (2024).
42. Sanyal, A.J., Jha, P. & Kleiner, D.E. Digital pathology for nonalcoholic steatohepatitis assessment. *Nature Reviews Gastroenterology & Hepatology* **21**, 57–69 (2024).

43. Chang, P.E., *et al.* Second harmonic generation microscopy provides accurate automated staging of liver fibrosis in patients with non-alcoholic fatty liver disease. *PLoS One* **13**, e0199166 (2018).
44. Sun, W., *et al.* Nonlinear optical microscopy: use of second harmonic generation and two-photon microscopy for automated quantitative liver fibrosis studies. *Journal of Biomedical Optics* **13**, 064010 (2008).
45. Ramatchandirin, B., *et al.* Protocol for measuring mitochondrial size in mouse and human liver tissues. *STAR Protoc* **5**, 102842 (2024).
46. Vialard, F. & Olivier, M. Thermoneutrality and Immunity: How Does Cold Stress Affect Disease? *Frontiers in Immunology* **Volume 11 - 2020**(2020).
47. Rahbani, J.F., *et al.* Parallel control of cold-triggered adipocyte thermogenesis by UCP1 and CKB. *Cell Metab* **36**, 526–540.e527 (2024).
48. Skop, V., *et al.* Mouse thermoregulation: Introducing the concept of the thermoneutral point. *The FASEB Journal* **34**, 1–1 (2020).
49. Chau, P.K.T., Ryan, E., Dalen, K.T. & Haugen, F. Timing of acute cold exposure determines UCP1 and FGF21 expression - Possible interactions between the thermal environment, thermoregulatory responses, and peripheral clocks. *Journal of Thermal Biology* **124**, 103938 (2024).
50. Zhang, Z., Cheng, L., Ma, J., Wang, X. & Zhao, Y. Chronic Cold Exposure Leads to Daytime Preference in the Circadian Expression of Hepatic Metabolic Genes. *Frontiers in Physiology* **Volume 13 - 2022**(2022).
51. (!!! INVALID CITATION !!! 27).
52. Naoumov, N.V., *et al.* Digital pathology with artificial intelligence analyses provides greater insights into treatment-induced fibrosis regression in NASH. *Journal of Hepatology* **77**, 1399–1409 (2022).
53. Taylor-Weiner, A., *et al.* A Machine Learning Approach Enables Quantitative Measurement of Liver Histology and Disease Monitoring in NASH. *Hepatology* **74**, 133–147 (2021).
54. Watson, A., Petitjean, L., Petitjean, M. & Pavlides, M. Liver fibrosis phenotyping and severity scoring by quantitative image analysis of biopsy slides. *Liver International* **44**, 399–410 (2024).

"AI digital pathology as a key tool providing in-depth understanding of the progression and regression of MASH and fibrosis in male mouse models"

Nature Communications manuscript NCOMMS-25-40823B

Point-by-point answers to the reviewers' questions

REVIEWER COMMENTS

Reviewer #1 (Remarks to the Author):

I appreciate the authors' efforts in addressing my previous concerns. However, I still have major concerns regarding the accuracy of newly developed AI model as specified below.

1. In response to my comment 3, the authors updated the inflammatory cell detection model using higher-resolution images. However, the validation of this model relies on a common conflation of correlation and accuracy. While they demonstrated a high correlation between the AI-based counts and the multiplexed immunofluorescence (mIF) gold standard (Supplementary Figure S4C), high correlation does not equate to absolute accuracy. Specifically, the AI model estimated only 300 immune cells in a section where mIF identified approximately 1,000. Because the model significantly underestimates the true cell count, it cannot yet be considered a reliable tool for accurate quantification.

Response:

We thank the reviewer for this important observation. We agree that high correlation does not equate to absolute accuracy, and that systematic underestimation must be carefully considered when interpreting AI-based quantification models. We clarify the intended use and rationale of the analysis below:

- **Different modalities measure related but distinct signals:** Our intent was not to present the inflammatory cell detection model as a direct replacement for absolute immune cell counting by mIF, but rather as a scalable and reproducible approach for relative immune burden assessment across large histological datasets. The observed underestimation likely reflects the inherent differences between H&E-based morphological detection and marker-based mIF, particularly in regions with closely apposed or phenotypically ambiguous immune cells. mIF detects cells by specific protein markers (biomarker-based, high sensitivity for target phenotypes), whereas H&E-based detection relies on morphology and contrast. Morphologically ambiguous cells (closely apposed cells, low contrast, or cells lacking clear morphological features) can be under-detected on H&E but captured by marker-based mIF; conversely, some H&E detections can be false positives if morphology alone is ambiguous. Therefore, absolute counts from these two approaches are not directly interchangeable.
- **Conservative parameter selection to avoid over-estimation:** To prioritize specificity and avoid inflating immune burden estimates, we intentionally selected stringent probability thresholds and required pathologist annotation of only unambiguous cells during training. As the reviewer noted, this leads to underestimation in some cases but minimizes false positives that would bias comparative analyses across samples.
- **Evidence of trade-offs:** We assessed the model across 60 (526 x 526 size) pathologist-annotated patches and an independent set of 80 mIF-validated images. As shown in sensitivity analysis in Supplementary Table S5 (Table R1 in the response), relaxing thresholds increases the number of

detected cells (up to ~4× in our most lenient tested setting) but is accompanied by a marked rise in false-positive rate (up to ~7×). In the mIF-validated set, using a more lenient parameter set increased counts by ~50% (Figure R1). Despite the differences in the absolute number of cells detected between the inflammatory cell detection model and mIF, preservation of strong correlation between different stringency thresholds (Figure R1) and between the different methodologies (Fig. S4C), indicate that rank order across samples is conserved even when absolute counts shift.

Given these modality differences and the trade-offs, we explicitly state the same in **Materials & Methods (AI-enabled multi-modal DP → Inflammatory cell detection)**:

"The model should be interpreted as a relative, not absolute, quantification tool."

"The StarDist and SAM thresholds were selected to balance the number of cells detected and false positive rate (Table S5)."

This conservative strategy is appropriate for the comparative and associative analyses presented in this work, where preservation of rank/order across samples (rather than exact per-sample counts) is the primary requirement.

We have included Table R1 as Table S5 in the Supplementary materials, while also clarifying that the model parameters were selected to balance the number of cells detected and the FPR.

Table R1. The relationship between the stringency of the parameter set for the inflammatory cell detection model and the number of detection as well as false positive, simulated on 60 test patches (526-pixel x 526-pixel) with pathologist ground truth. The model was run by varying the probability thresholds on the two stages of the AI inflammatory cell detection pipeline: (1) cell detection by StarDist, followed by (2) inflammation classification by Segment Anything Model (SAM). StarDist Non-Maximum Suppression (NMS) for determining overlapping cells was set constant at 0.1.

AI parameter threshold set		Average number of inflammatory cells detected per patch	Increase in the number of inflammatory cells detected*	FPR	Increase in FPR*
StarDist	SAM				
0.5	0.3	29.3	4.1	13.4%	7.2
0.7	0.3	24.0	3.3	10.7%	5.8
0.8	0.3	13.7	1.9	5.5%	3.0
0.5	0.5	14.9	2.1	4.6%	2.5
0.7	0.5	12.3	1.7	3.6%	1.9
0.8	0.5	7.2	1	1.9%	1

*The increase is calculated with respect to the value for the most stringent parameter set. Grey highlight indicates the current model parameter set.

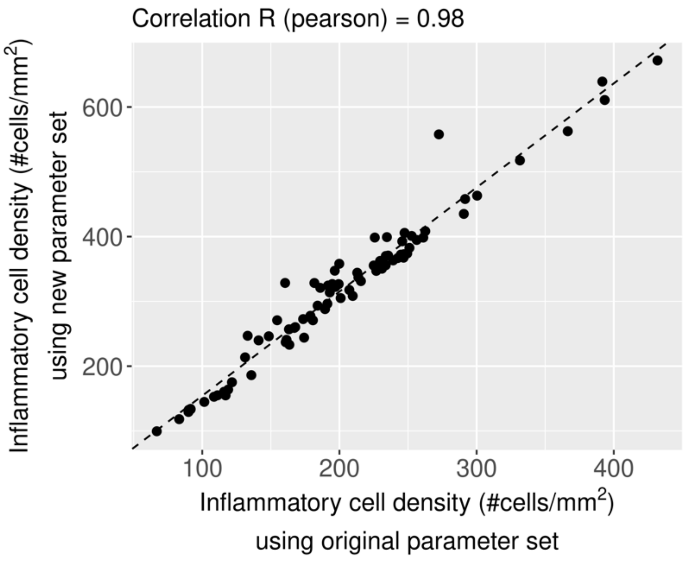

Figure R1. Correlation between the number of cells detected on the H&E section using a more lenient model parameter set (StarDist probability threshold: 0.5, SAM probability threshold: 0.3) vs. current model parameter (N=80 WSIs)

2. The biological significance of pigmented granulomas remains speculative. To justify the inclusion of this feature in the model, the authors should provide data regarding its association with patient outcomes or therapeutic responses. Without establishing a clear clinico-pathological correlation, this AI-based detection tool lacks a defined purpose in a diagnostic or prognostic setting.

Response:

We thank the reviewer for raising the important issue of biological and clinical interpretation. We agree that the prognostic and therapeutic significance of pigmented (lipo)granulomas is context-dependent and not yet fully established.

Pigmented granulomas are a recognized histopathological feature observed in chronic liver injury and treatment-associated tissue remodeling, but they are typically described qualitatively and inconsistently across studies (see references in bullets). Their inclusion in the model was therefore motivated by a methodological objective: to enable systematic, reproducible detection of such features and to support future hypothesis-generating analyses.

As already discussed in the manuscript (Discussion, "A notable morphometric…unsuitable standalone staging metric"), we explicitly state that granulomas are unsuitable as standalone staging or prognostic metrics due to their temporal heterogeneity and potential reversibility. We further note that their biological impact is shaped by diet, injury duration, iron load, oxidative stress, and the fibrogenic response, underscoring the absence of a single, generalizable clinico-pathological association.

The inclusion of pigmented granulomas in the AI framework was therefore not intended to imply immediate diagnostic or prognostic utility, but rather to demonstrate the capacity of the model to systematically and reproducibly detect histological features whose significance may be temporally dynamic and context-specific. As outlined in the manuscript, a key purpose of this approach is to enable future hypothesis-driven studies such as integration with spatial omics, laser-capture microdissection, and longitudinal intervention models to establish causal, therapeutic, or outcome-linked relevance. To further minimize potential misinterpretation, we have reviewed the Results and Discussion sections to ensure that no claims of clinical utility are implied and that the exploratory nature of granuloma detection is clearly emphasized.

- Almadi, M.A., et al. New insights into gastrointestinal and hepatic granulomatous disorders. Nature Reviews Gastroenterology & Hepatology 8, 455–466 (2011).
- Sobczak-Filipiak, M., et al. Lipogranulomas and pigment granulomas in livers of dogs with portosystemic shunt. Polish Journal of Veterinary Sciences vol. 21, 265–272 (2018).
- Zaki, M.Y.W., et al. Key features of the environment promoting liver cancer in the absence of cirrhosis. Scientific Reports 11, 16727 (2021).
- Zhu, H., Jr, H.C., Clain, D., Min, A. & Theise, N. Hepatic lipogranulomas in patients with chronic liver disease: Association with hepatitis C and fatty liver disease. World Journal of Gastroenterology 16, 5065–5069 (2010).

3. As noted by other reviewers, it is essential for the authors to demonstrate the applicability of this model to human MASH samples. At a minimum, providing preliminary data from a small cohort of human MASH cases would significantly strengthen the study by confirming that the model's performance translates from experimental models to clinical pathology.

Response:

We thank the reviewer for highlighting the importance of demonstrating applicability to human MASH pathology. We agree that human validation is essential for clinical translation.

We note that the AI digital pathology framework used in the present study has already been applied and validated in human MASH biopsies for fibrosis quantification, as reported in "Utility of AI digital pathology as an aid for pathologists scoring fibrosis in MASH. https://doi.org/10.1016/j.jhep.2024.11.032". That study demonstrated concordance between AI-derived fibrosis measures and expert pathologist scoring in human samples, supporting the translational applicability of the core model architecture and analytical approach. The current work builds on this established human validation by extending the framework to additional histological features and mechanistic readouts. These extensions were intentionally developed and rigorously benchmarked in experimental models, where controlled perturbations, standardized tissue processing, and ground-truth annotations are feasible. Direct application of these newly developed features to human biopsies would require dedicated calibration and appropriately powered cohorts to avoid overinterpretation.

Accordingly, we position the present study as a foundational extension of an AI pathology framework with demonstrated human applicability for fibrosis.

Additionally, we have revised the discussion section to clarify that clinical decision making represents a future application contingent on human validation.

The revised sentence in the **Discussion section** is:

"Collectively, these advances position AI to accelerate translational research and with appropriate human validation, inform clinical decision-making in liver disease, with the ultimate goal of improving patient outcomes."

Reviewer #2 (Remarks to the Author):

The authors have responded appropriately to my review comments and made changes to the revised paper. I have no additional comments.

Reviewer #3 (Remarks to the Author):

Thank you to the authors for their well-written and comprehensive rebuttal letter incl. explanatory figures and the revised version of the manuscript. Responses are detailed, clear and all comments were addressed appropriately.

One major remaining point concerns the appropriate acknowledgement of the substantial scientific expert contribution of involved human and / or veterinary pathologists. Please add the initials in the M&M and author contribution sections.

Response :

We thank the reviewer for highlighting the importance of appropriately acknowledging expert pathological contributions. We have revised the manuscript to reflect the same in the following sections (in addition to the acknowledgement section) :

Material & Methods → Histopathology sub-section:

"Histopathological evaluation including assessment of cellular and sub-cellular features from tissue sections was performed by board-certified pathologists Y.Z. (from Histobridge) and R.R. (from AMPL)"

Acknowledgement section:

"We are thankful for the expertise of Yonghua Zhu (Histobridge) and Ravisankar Rajarhetinam (AMPL) for their expert histopathological assessment and interpretation."

Reviewer #4 (Remarks to the Author):

I co-reviewed this manuscript with one of the reviewers who provided the listed reports. This is part of the Nature Communications initiative to facilitate training in peer review and to provide appropriate recognition for Early Career Researchers who co-review manuscripts.

Reviewer #5 (Remarks to the Author):

The authors have addressed the points raised previously. Thank you.

Reviewer #5 (Remarks on code availability):

The code provides instructions for running the application.

Reviewer #6 (Remarks to the Author):

I co-reviewed this manuscript with one of the reviewers who provided the listed reports. This is part of the Nature Communications initiative to facilitate training in peer review and to provide appropriate recognition for Early Career Researchers who co-review manuscripts.
